# The caterpillar fungus, *Ophiocordyceps sinensis*, genome provides insights into highland adaptation of fungal pathogenicity

**DOI:** 10.1038/s41598-017-01869-z

**Published:** 2017-05-11

**Authors:** En-Hua Xia, Da-Rong Yang, Jian-Jun Jiang, Qun-Jie Zhang, Yuan Liu, Yun-Long Liu, Yun Zhang, Hai-Bin Zhang, Cong Shi, Yan Tong, Changhoon Kim, Hua Chen, Yan-Qiong Peng, Yue Yu, Wei Zhang, Evan E. Eichler, Li-Zhi Gao

**Affiliations:** 1 Plant Germplasm and Genomics Center, Germplasm Bank of Wild Species in Southwestern China, Kunming Institute of Botany, the Chinese Academy of Sciences, Kunming, 650204 China; 2 Institution of Genomics and Bioinformatics, South China Agricultural University, Guangzhou, 510642 China; 3 University of the Chinese Academy of Sciences, Beijing, 100039 China; 4 Xishuangbanna Tropical Botanical Garden, The Chinese Academy of Sciences, Menglun, 666303 China; 5 Agrobiological Gene Research Center, Guangdong Academy of Agricultural Sciences, Guangzhou, 510640 China; 6 Marcogene Inc., Seoul, 08511 South Korea; 7 Center for Computational Genomics, Beijing Institute of Genomics, The Chinese Academy of Sciences, Beijing, 100101 China; 8 Department of Genome Sciences, University of Washington School of Medicine, Seattle, WA 98195 USA; 9 Howard Hughes Medical Institute, University of Washington, Seattle, WA 98195 USA

## Abstract

To understand the potential genetic basis of highland adaptation of fungal pathogenicity, we present here the ~116 Mb *de novo* assembled high-quality genome of *Ophiocordyceps sinensis* endemic to the Qinghai-Tibetan Plateau. Compared with other plain-dwelling fungi, we find about 3.4-fold inflation of the *O*. *sinensis* genome due to a rapid amplification of long terminal repeat retrotransposons that occurred ~38 million years ago in concert with the uplift of the plateau. We also observe massive removal of thousands of genes related to the transport process and energy metabolism. *O*. *sinensis* displays considerable lineage-specific expansion of gene families functionally enriched in the adaptability of low-temperature of cold tolerance, fungal pathogenicity and specialized host infection. We detect signals of positive selection for genes involved in peroxidase and hypoxia to enable its highland adaptation. Resequencing and analyzing 31 whole genomes of *O*. *sinensis*, representing nearly all of its geographic range, exhibits latitude-based population divergence and nature selection for population inhabitation towards higher altitudes on the Qinghai-Tibetan Plateau.

## Introduction

Entomopathogenic fungi are attracting attention as potential biological control agents of insect pests, which pose significant challenges for jeopardizing world food security and protecting economically and ecologically important host populations in nature^1, 2^. Over the past decade, there have been many attempts to exploit exceptional evolutionary mechanisms in animals and humans to adapt to extremely inhospitable habitats on the Qinghai-Tibetan Plateau^3–7^; however, very little is known about the genomic basis underlying the fungal pathogenicity and potential highland adaptation during interaction between fungal pathogens and host insects.

The Chinese caterpillar fungus, *Ophiocordyceps sinensis* (also known as *Cordyceps sinensis*), the so-called “winter worm, summer grass” in Chinese literature^8^, is one of the most outstandingly valued traditional Chinese medicinal fungi^9^. This fungus exhibits extremely high host specificity and colonizes ghost moth caterpillars (*Thitarodes* spp.), making a parasitic complex that comprises the remains of the caterpillar and fungal sexual stroma. It exclusively inhabits the harsh alpine environments of the Qinghai-Tibetan Plateau with an average altitude of over 4,000 m above sea level. Although *O*. *sinensis* has an optimum growth temperature at 18 °C, it is able to suffer temperatures lower than −40 °C in winter as a psychrophile^9^. First documented in the 15th century^10^, this parasitic complex has been highly prized for its treasured medical benefits over the past centuries. Former studies have isolated diverse bioactive ingredients and found their extensive medicinal effects in *O*. *sinensis* ^9, 11^, which has been applied to treat a variety of ailments including cancer, impotence and fatigue^12, 13^. In recent decades, huge world market demands have led to overexploitation, severely endangering numerous natural populations of *O*. *sinensis* towards its extinction in nature and damaging its habitats in the alpine ecosystem associated with the birthplace of most rivers comprising the Asian river system, which serves most of the Asian populations^14^. Although *O*. *sinensis* grows slowly on artificial media and unceasing efforts to cultivate the fungus have failed to generate fruiting bodies so far, the locally sampled materials with newly initiated fruiting bodies could successfully complete its sexual development under experimental conditions^15^. This suggests that the induction of sexual processes may be associated with a kind of puzzling ecological factors specific to the alpine ecosystem of the Qinghai-Tibetan Plateau. In contrast to *O*. *sinensis*, however, the closely related and out-crossing fungal species *C*. *militaris*, which thrives at low altitude regions, has been commercially cultivated. Although the interaction between the caterpillar and fungus of *O*. *sinensis* can be establish under natural conditions over 4,000 m above sea level, many biological aspects surrounding the cultivation of *O*. *sinensis* remain mysterious largely owing to difficulties in observing fungal development and infection processes in the wild. Thus, the genome sequence of *O*. *sinensis* and comparative analyses with the previously available high-quality genome of *C*. *militaris* can shed light on the genetic basis of fungal pathogenicity and genomic components that are shaped by high-altitude adaptation in the Qinghai-Tibetan Plateau.

The first draft genome sequence of *O*. *sinensis* was formerly released with a particular emphasis on studies of the sexuality and lifestyle in this caterpillar fungus^15^. However, the low quality of the generated genome assembly (a contig N50 size of 5,253 bp; total length of scaffolds of 78.52 Mb with an estimation of 65.43% completeness of the genome) has largely restricted an accurate genome annotation that is the key to understand the mechanisms of fungal pathogenicity and high-altitude adaptation in fungi as well as its future applications to germplasm conservation, successful cultivation and breeding programs of *O*. *sinensis*.

Here, we report a high-quality reference genome assembly of *O*. *sinensis* from Nyingchi of Tibet, China, based on sequence data from whole-genome shotgun (WGS) sequencing platforms of Roche 454 and Illumina HiSeq 2000 technologies. This assembly contains 156 scaffolds (>2 Kb; N50 = ~3.0 Mb), has a length of ~116.4 Mb and covers ~97% of the predicted genome size (~120 Mb). Together with the data analyses from re-sequencing samples of the 31 representative natural populations, comprehensive transcriptomic surveys of the three major developmental stages and comparative genomic analyses with *C*. *militaris*, we aim to obtain new insights into molecular mechanisms of fungal pathogenicity, mating system evolution of the fungal species and the adaptation to living at high altitudes on the Qinghai-Tibetan Plateau.

## Results

### Genome sequencing, assembly and annotation

We sequenced *O*. *sinensis* from the Nyingchi District of Tibet, China. We performed a WGS analysis with the next-generation sequencing Roche 454 and Illumina HiSeq 2000 platforms. This generated clean sequence data sets of ~5.4 Gb, thus yielding approximately 45.1-fold genome coverage, respectively (Supplemental Table S1). We estimated that the genome size is ~124.08 Mb and ~119.8 Mb based on flow cytometry and 17-mer depth distribution of sequenced reads, respectively (Supplemental Figures S1–2 and Supplemental Table S2). The *O*. *sinensis* genome was first assembled from Roche 454 long reads using Newbler^16^, followed by scaffolding pre-assembled contigs with Illumina mate pair sequencing reads using SSPACE^17^. This finally yielded a ~116.4 Mb genome assembly that covers ~97% of the estimated genome size, and contains 156 scaffolds (>2 Kb) with a ScafN50 value of ~3 Mb and 9,141 contigs (N50 = 21,423 bp) (Table 1 and Supplemental Tables S3–4). To validate quality of the genome assembly, we first aligned all DNA and expressed sequence tags (ESTs) of *O*. *sinensis* available in the public databases and obtained mapping rates of 98.85% and 95.33%, respectively (Supplemental Table S5). Second, we mapped all clean Roche 454 long reads (~1.84 Gb) to the assembled genome sequences, showing an almost perfect alignment with a mapping rate of 99.01% (Supplemental Table S5). Third, the transcripts we assembled showed a good alignment to the assembled genome; of 11,742 transcripts, 91.29% were mapped (transcript coverage ≥80% and identity ≥90%; Supplemental Table S5). Finally, we evaluated the completeness of our *O*. *sinensis* assembly using BUSCO^18^; 94.0%, 4.0% and 1.8% of the 1,315 expected Ascomycota BUSCO conserved genes were identified as complete, fragmented and missing in our *O*. *sinensis* assembly, respectively (Supplemental Table S5).

**Table 1:** Comparison of genome features between *O*. *sinensis* and *C*. *militaris*.

Genome features	*O*. *sinensis*	*C*. *militaris* ^a^
Assembled genome size (Mb)	116.42	32.27
Contig N50 (bp)^b^	21,423	105,531
Scaffold N50 (bp)^b^	2,999,605	4,551,492
Content of repeat sequences (%)	74.67	3.04
Predicted protein-coding genes (#)	7,939	9,684
Average gene length (bp)	1,693	1,743
Average CDS length (bp)	1,504	1,517
Average intron length (bp)	103	113
Average exon number per gene	2.8	3.0

^a^From Zheng *et al*. *Genome Biology* 2011, 12:R116; ^b^N50 values of the genome assemblies were calculated using the fragments ≥1 kb.

We generated ~15.05 Gb of RNA sequencing (RNA-Seq) data obtained from a total of six libraries representing the three major developmental stages to aid gene prediction (Supplemental Figure S4 and Supplemental Tables S6,7). In combination with *ab initio* prediction, protein and EST alignments, EvidenceModeler combing and further filtering, we defined 7,939 protein-coding genes (Table 1 and Supplemental Table S8). Of these predicted genes, approximate 97.0% and 71.51% could be functionally classified and supported by RNA-Seq data, respectively (Supplemental Tables S9–11). Using the BUSCO from Ascomycota lineage, we further found that 94.4%, 3.6%, 1.8% and 0.2% of the genes were complete, fragmented, missing and duplicated, respectively, indicating a good quality of our gene annotation (Supplemental Table S11). We also performed homologue searches and annotated noncoding RNA (ncRNA) genes, yielding 146 transfer RNA (tRNA) genes, 33 ribosomal RNA (rRNA) genes, 70 small nucleolar RNA (snoRNAs) genes, and 15 small nuclear RNA (snRNA) genes (Supplemental Figure S6 and Supplemental Table S12). The annotation of repeat sequences presented that transposable elements (TEs) accounted for approximately 74.67% of the assembled genome and 80.07% of the raw reads, indicating that ~5.45% of the unassembled genome consists of TEs (Supplemental Tables S13–14). The GC content was 43.09% across the genome and 61.49% in coding sequences (Supplemental Figure S3; Supplemental Tables S4 and S8). We annotated 8,918 simple sequence repeats that will provide valuable genetic markers to assist future breeding programs of Chinese caterpillar fungus (Supplemental Tables S15–16 and Supplemental Figure S7).

### Retrotransposon-driven genome expansion and massive removal of non-collinear genes

Comparison of genome sizes showed that the *O*. *sinensis* genome was nearly 3.4-fold larger than other entomopathogenic fungi in the Hypocreales family (Supplemental Table S17 and Supplemental Figure S8A). Repeat sequence analysis revealed that this expansion was primarily due to a rapid proliferation of transposable elements. Approximately 74.67% of the *O*. *sinensis* genome assembly was composed of repeat sequences (Supplemental Tables S13–14), exceptionally larger than those reported in *Metarhizium anisopliae* (~0.98%)^19^, *Metarhizium acridum* (~1.52%)^19^, *Cordyceps militaris* (~3.04%)^20^ and *Beauveria bassiana* (~2.03%)^21^ (*P* < 4.822e-07) (Supplemental Figure S8B). The MULE elements were notably the most abundant, accounting for ~1.6% (~1.9 Mb) of the *O*. *sinensis* genome and more than 59% of DNA transposons in *O*. *sinensis*. Retrotransposons, mostly long-terminal repeat (LTR) retrotransposons, comprised ~59.76% of the *O*. *sinensis* genome and large-scale proliferation of which occurred approximately at ~38 million years ago (MYA) (Supplemental Figure S9).

**Figure 1:**
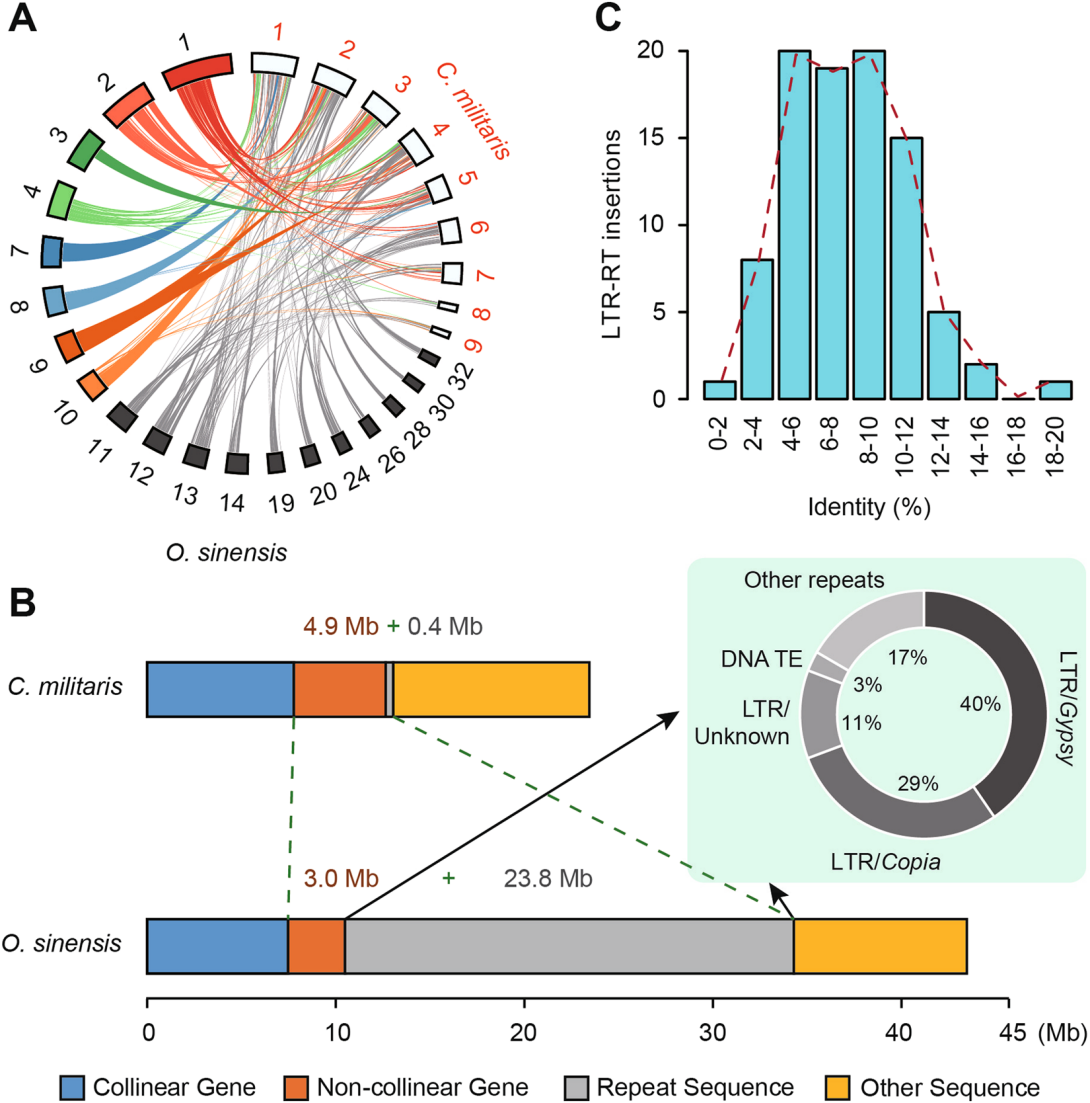
Genome size variation. (**A**) Collinear blocks between *O*. *sinensis* and *C*. *militaris*. The largest nine scaffolds of *C. militaris* are highlighted with red numbers. Whole scaffolds are depicted in Supplemental Figure S10. Collinear blocks are identified using MCScanX package with default parameters. (**B**) Differences in genomic composition. Approximate 23.4 Mb (72.7% of the total genome) and 43.5 Mb (37.5%) of the *C*. *militaris* and *O*. *sinensis* genomes are mapped to 308 syntenic blocks. Significant expansion of LTR retrotransposons and the loss of non-collinear genes are observed. (**C**) LTR retrotransposon expansion in collinear blocks of *O*. *sinensis*. X-axis indicates percent identity of LTRs, whereas y-axis represents the number of LTR retrotransposon insertions.

In contrast to rapid amplification of LTR retrotransposons driving the expansion of the *O*. *sinensis* genome, another remarkable feature is the dramatic loss of protein-coding genes in the *O*. *sinensis* lineage in comparison to other entomopathogenic fungi. Compared to a total of 7,939 protein-coding genes in *O*. *sinensis* there were more than 10,095 genes on average in other entomopathogenic fungi, e.g., *Metarhizium anisopliae* (10,582)^19^, *Metarhizium acridum* (9,849)^19^, *Cordyceps militaris* (9,684)^20^, *Beauveria bassiana* (10,366)^21^ and *Tolypocladium inflatum* (9,998)^22^ (Table 1). Such a reduction of gene number was further evidenced by the identification of non-collinear genes and comparative analysis of synteny blocks between the *O*. *sinensis* and *C*. *militaris* genomes. We identified a total of 308 syntenic blocks that span nearly 72.7% (~23.4 Mb in *C*. *militaris* vs. ~43.5 Mb in *O*. *sinensis*) of the *C*. *militaris* genome (Fig. 1A; Supplemental Figure S10 and Supplemental Tables S18–19). Of these syntenic genomic regions, there was a decrease of non-collinear genes in *O*. *sinensis* (2,127) compared to *C*. *militaris* (3,259) but an increase of repeat sequences (23.8 Mb in *O*. *sinensis* vs. 0.40 Mb in *C*. *militaris*) (Fig. 1B and Supplemental Table S19). Functional annotation of the 2,468 genes that were lost in *O*. *sinensis* showed they were mainly involved in amino acid metabolism, such as biosynthesis of amino acids (ko01230), arginine and proline metabolism (ko00330), and tyrosine metabolism (ko00350) (Supplemental Figure S11 and Supplemental Table S20). Notably, nearly 81% of repeat sequences in these 308 syntenic blocks were LTR retrotransposons, 40.4% of which were *Gypsy* retroelements (Fig. 1B and Supplemental Table S19). Molecular dating estimated that this particular class of LTR retrotransposons amplified ~38 Mya, which is consistent with the uplift of the Qinghai-Tibetan Plateau (Fig. 1C).

### Rapid evolution of gene families related to fungal pathogenicity

**Figure 2:**
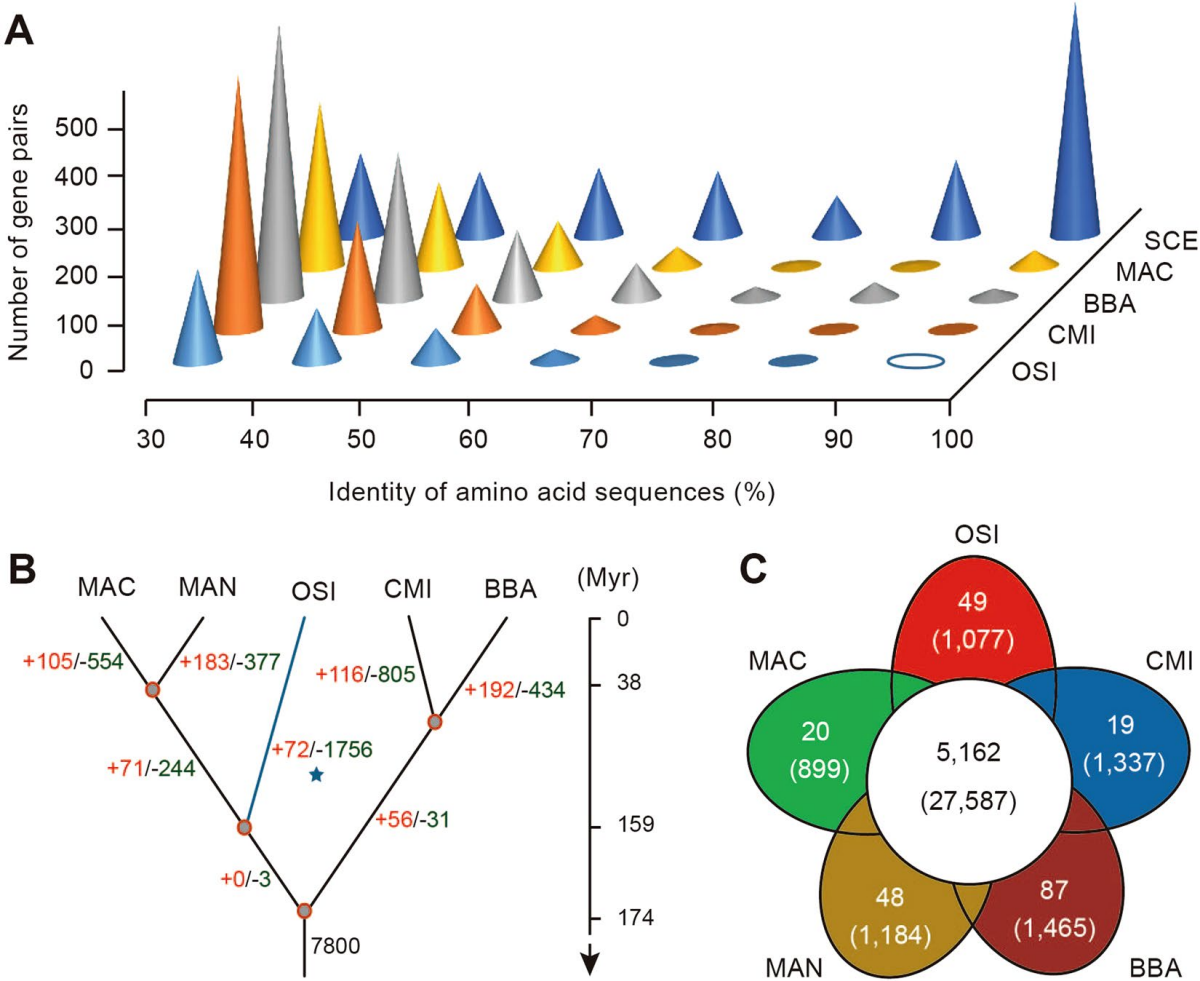
Gene family evolution. (**A**) Characterization of the paralogous genes among five entomopathogenic fungi. Abbreviations: OSI, *O*. *sinensis*; MAN, *M*. *anisopliae*; MAC, *M*. *acridum*; CMI, *C*. *militaris*; BBA, *B*. *bassiana; SCE, ﻿S. ce﻿rev﻿isiae*. The x-axis shows the amino acid identity for each paralogous pair, while z-axis indicates the total number of paralogous genes within identity groups. Paralogous gene pairs are detected based on all-*vs*-all comparisons within the same species using Blastall program (version 2.2.26). (**B**) Gene expansion and contraction in the *O*. *sinensis* genome. The numbers of gene families showing expansion (red) or contraction (green) for each lineage after speciation are indicated on each branch of the phylogenetic tree with the position of *O*. *sinensis* highlighted (blue asterisk). (**C**) Venn diagram depicting unique and shared gene families among the five fungal genomes. Actual gene numbers are indicated (parentheses).

One of the most striking characteristics of the *O*. *sinensis* genome is the dearth of highly homologous gene pairs. Of the predicted 7,939 protein-coding genes, no pairs shared >90% amino acid identity in coding sequences and there was only one pair that shared >80% amino acid identity (Fig. 2A and Supplemental Table S21). This feature was also observed in the closely related *C*. *militaris* and ectomycorrhizal fungus *Tuber melanosporum* ^23^. Compared with other entomopathogenic fungi like *B*. *bassiana* and *C*. *militaris*, multigene families in *O*. *sinensis* were limited in number and comprised only 8.7% of the predicted proteome; most gene families had only two members (Supplemental Figure S12). The rate of gene gain was strikingly lower than that of gene loss, and among the 7,800 gene families found in the most recent common ancestor (MRCA) of Hypocreales, 1,756 were seemingly lost in *O*. *sinensis* (Fig. 2B). Such a compact gene coding space of the *O*. *sinensis* genome suggests the nature of this highly specialized fungus with a low capacity to adapt to multiple environmental cues.

To understand the evolution of gene families that relate to fungal pathogenicity and highland adaptation to harsh environments, we investigated the functional properties of gene families that have undergone expansions or contractions in *O*. *sinensis*. The *O*. *sinensis* genome showed a considerable expansion of gene families that are mainly involved in fungal pathogenicity, including peroxidase activity (PF01328; *P* < 0.01), serine hydrolase (PF03959; *P* < 0.01), deuterolysin metalloprotease (M35) peptidase (PF02102; *P* < 0.01), and cytochrome P450 (PF00067; *P* < 0.01) (Supplemental Table S23). Interestingly, we found that the expanded gene families are also functionally enriched in the Pfam category of glucose-methanol-choline (GMC) oxidoreductase involved in the ecdysteroid metabolism of molting in insects (Supplemental Table S23). In comparisons with other entomopathogenic fungi, the gene family expansion in the *O*. *sinensis* lineage was also observed with over-representing Pfam terms putatively related to the adaptation of low temperature (PF06772; *P* < 0.01) (Supplemental Table S23).

In contrast, gene families exhibiting contraction status were mainly involved in the transport process and energy metabolism, such as ABC transporters (PF00005; *P* < 0.01), amino acid permease (PF00324; *P* < 0.01), and ATP synthase (PF00306; *P* < 0.05) (Supplemental Table S24). Apart from dynamic evolution of these gene families, we further detected 1,077 (~13.57%) species-specific genes in *O*. *sinensis* (Fig. 2C). Of them, 318 (~29.53%) genes could be functionally annotated and were significantly enriched in GO categories associated with starch binding (GO: 2001070; *P* < 0.01), pathogenesis (GO: 0009405; *P* < 0.01), and cell wall macromolecule catabolic process (GO: 0016998; *P* < 0.01) (Supplemental Table S25).

**Figure 3:**
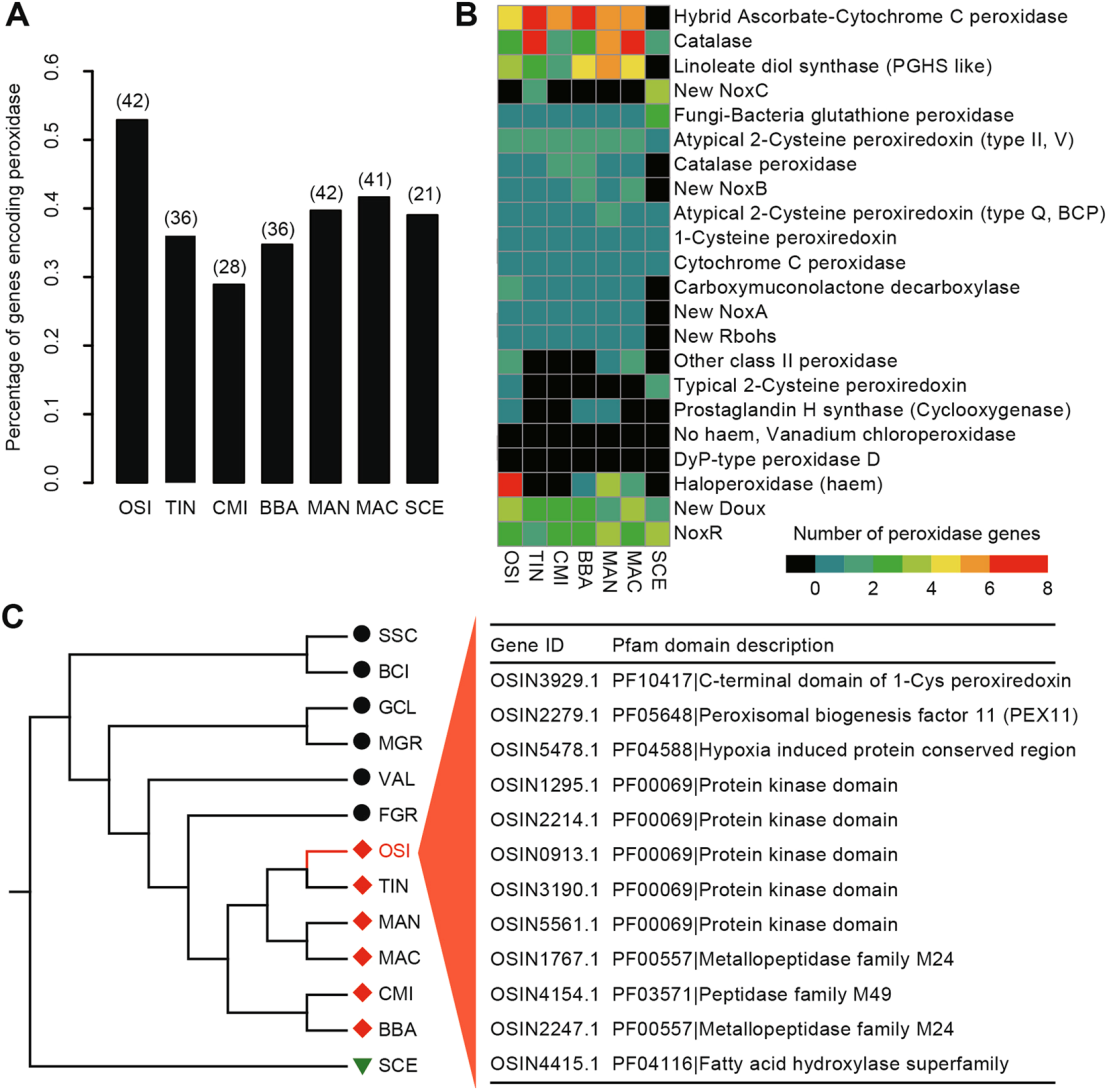
Analysis of peroxidase genes and positive selection. (**A**) Comparisons of the percent and number (parentheses) of peroxidase genes among the five entomopathogenic fungi and yeast (*S*. *cerevisiae*). Abbreviations: TIN, *T*. *inflatum*; SCE, *S*. *cerevisiae*. (**B**) Peroxidase gene subtypes are defined by fPoxDB (http://peroxidase.riceblast.snu.ac.kr). The number of genes within each peroxidase class are presented using “*pheatmap*” package implemented in R program (version 3.0.1). (**C**) Positively selected genes (PSGs) detected in *O*. *sinensis* lineage. 12 PSGs potentially associated with high-altitude adaptation and host infection are shown (right panel). Phylogenic relationships among 13 fungal species (left panel). Plant pathogens are labeled with black solid circles, whereas insect pathogens are colored in red diamonds. *S*. *cerevisiae* was selected as outgroup and depicted in a green triangle.

To avoid the infection of fungal pathogens, insect hosts often rapidly produce plenty of reactive oxygen species (ROS) to directly kill pathogens. As a response, pathogens evolved the ROS antioxidant defense system during the evolution, of which peroxidases, acting as ROS-scavenging enzymes, are regarded as one of the most prominent and integral components^24, 25^. Among the expanded genes in *O*. *sinensis*, peroxidase activity was one of the highly enriched functional categories (Supplemental Table S23). Hidden Markov model (HMM) searches revealed 42 (~0.53%) peroxidase genes in *O*. *sinensis*, the number of which was remarkably larger than that of *C*. *militaris* (28) and yeast (21), suggesting that a two-fold expansion of peroxidase genes might potentially result in a strong capacity to aid ROS detoxification in *O*. *sinensis* (Fig. 3A and Supplemental Table S26). Among these 42 peroxidase genes, haloperoxidase (haem) is the most abundant, accounting for ~16.67% of the total peroxidases detected (Fig. 3B). In contrast to other closely related fungal species that completely lack the typical 2-Cysteine peroxiredoxin, *O*. *sinensis* still retains one copy (Fig. 3B). 2-Cys peroxiredoxin was previously shown to play a role in responding to different levels of oxidative stress in *Vibrio vulnificus* ^26^. A comparative analysis revealed that the retained gene in *O*. *sinensis* belongs to the *Prx1* type, which was reported to be functionally conserved^27^ and expressed only when the cells are exposed to high levels of H_2_O_2_ generated exogenously^26^.

Unlike the infection mechanism of plant pathogens (PPs), which requires carbohydrate-active enzymes (CAZymes) to degrade the complex plant cell wall^28^, insect pathogens (IPs) typically infect their hosts by penetrating the cuticle^29^. To test this, we compared *O*. *sinensis* and four more insect pathogens (*M*. *anisopliae*, *M*. *acridum*, *C*. *militaris* and *B*. *bassiana*) with the four plant pathogens (*Fusarium graminearum*, *Magnaporthe grisea*, *Grosmannia clavigera* and *Botrytis cinerea*) (Supplemental Table S17). Our results demonstrated that insect pathogens had more proteases (on average, 362 in IPs *vs*. 342 in PPs; *P* < 0.43) and protein kinases (on average, 151 in IPs *vs*. 119 in PPs; *P* < 0.0014) to degrade the insect cuticle compared with plant pathogens (Supplemental Tables S27–29). In contrast, plant pathogens harbored more CAZymes than insect pathogens for plant cell wall degradation (on average, 161 in IPs *vs*. 231 in PPs) (Supplemental Tables S30–32). Excluding plant pathogens, *O*. *sinensis* remarkably had fewer genes encoding proteases (260) than other insect pathogens, such as *M*. *anisopliae* (437), *M*. *acridum* (361), *C*. *militaris* (355), and *B*. *bassiana* (396). However, ~35% of these proteases characterized in *O*. *sinensis* contain a signal peptide that is more likely to have been involved in pathogen-host interactions (Supplemental Tables S10 and S34), which is larger than that in other entomopathogenic fungi (on average, 20%). Similar to the other insect pathogens, several cellulase families, including GH7, GH45, and GH51, also declined or were absent in *O*. *sinensis* (Supplemental Table S30).

We also examined gene expression profiles across the three developmental stages of *O*. *sinensis* with length ratios of fungus *vs*. insect reaching ~1.20×, ~1.75× and ~2.20×. The results show that a total of 411 genes were differentially expressed (DEG) among the three developmental stages (Supplemental Figure S14). Functional annotation of these 411 DEGs found that they were mainly involved in fungal pathogenicity, such as glycosyl hydrolases family 16 (PF00722; FDR < 0.01), cytochrome P450 (PF00067; FDR < 0.01) and major facilitator superfamily (PF07690; FDR < 0.05). Besides, genes encoding enzymes associated with mitochondrial respiratory chain were also functionally enriched, such as NAD dependent epimerase/dehydratase family (PF01370; FDR < 0.01) and BCS1 N-terminal domain (PF08740; FDR < 0.01) (Supplementary Table S33).

### Positive Darwinian selection serves as driving forces for fungal pathogenicity

Positive selection has undoubtedly played a critical role in the evolution of diverse organisms living in high-altitude environments of the Qinghai-Tibetan Plateau, and many of the phenotypic traits are likely to show such selection signatures^3–5^. Of the 1,499 high-confidence single-copy orthologues shared between *O*. *sinensis* and the other 12 fungal species, 163 positively selected genes (PSGs) were identified in *O*. *sinensis* by using the branch-site likelihood ratio test (LRT; *P* < 0.05) (Supplemental Table S35). Of them, one gene (*OSIN3929*; here named *OsPRX1*) has been functionally implicated in peroxidase activity (Fig. 3C). This gene is a member of the peroxiredoxin family with 1-cysteine and highly homologous to *PRX1* (YBL064C) in *S*. *cerevisiae* ^30^. *PRX1* genes in *S*. *cerevisiae* and two human pathogenic fungi, *A*. *fumigatus* and *C*. *albicans*, are functionally conserved and required for detoxification of the oxidative burst within host cells^31, 32^. In particular, the deletion of *PRX1* in the well-known rice pathogen, *Magnaporthe oryzae*, resulted in an almost complete loss of pathogenicity, suggesting that this peroxidase is key to host-pathogen interactions^27^. Strikingly, several genes involved in host-pathogen interactions, including peroxisomal biogenesis, protein kinase and metallopeptidases, were also detected to be under positive selection (Fig. 3C).

### Mating system evolution

In ascomycetous fungi, the mating system is usually controlled by the mating-type (*MAT*) locus^33^. Our genome sequencing analysis found that *O*. *sinensis* not only possessed the *MAT1-2-1* mating type gene within the *MAT1-2* idiomorph but also had three mating-type genes (i.e., *MAT1-1-1*, *MAT1-1-2*, and *MAT1-1-3*) within the *MAT1-1* idiomorph (Supplemental Figure S15B). This feature was verified using whole-genome resequencing of 31 natural populations across nearly the entire geographic range, indicating that *O*. *sinensis* is indeed homothallic (Supplemental Figure S15A and Supplemental Table S36). The characteristic is extremely different from its closely related fungal pathogens, such as *Tolypocladium inflatum* (MAT1-2)^22^, *C*. *militaris* (MAT1-1)^20^, *B*. *bassiana* (MAT1-1)^21^, *M*. *anisopliae* (MAT1-1)^19^, and *M*. *acridum* (MAT1-2)^19^, which are heterothallic and possess only a single mating-type locus. Similar to the well-known homothallic plant pathogen *Fusarium graminearum* ^34^, the organization of these two *MAT* loci in *O*. *sinensis* revealed the fusion status within the idiomorphic genomic region, which was particularly enriched in LTR retrotransponsons. The split between the homothallic *O*. *sinensis* and heterothallic *C*. *militaris* was estimated to occur nearly 174.2 MYA (Supplemental Figure S13C), and was subjected to multiple conversions of the mating system from self-incompatible to self-compatible during the course of its evolutionary history (Supplemental Figure S15C), resembling the filamentous ascomycete genus of *Neurospora* ^35^.

### Population divergence based on latitudes on the Qinghai-Tibetan Plateau

**Figure 4:**
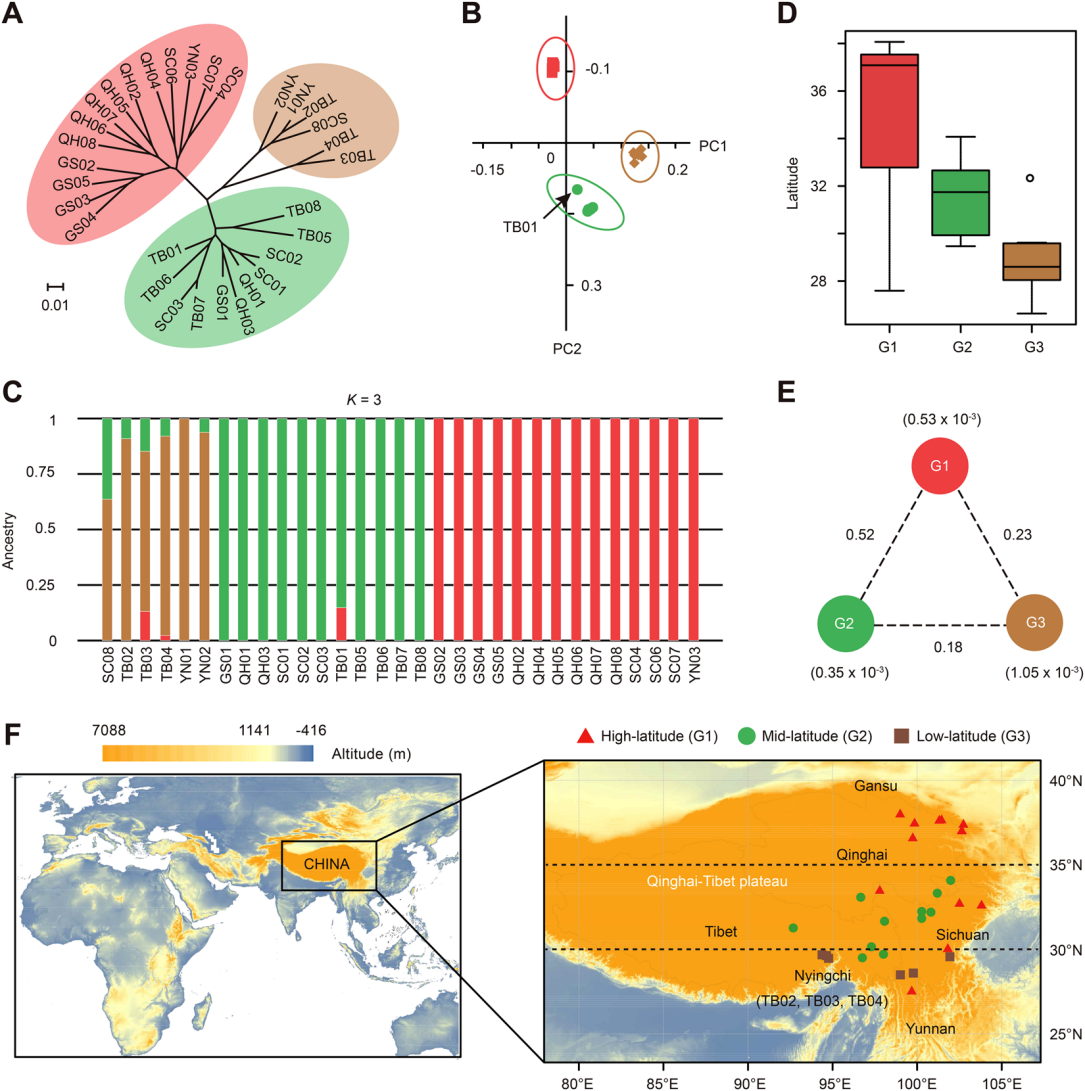
Latitude-based population divergence of *O*. *sinensis*. (**A**) Neighbor-Joining (NJ) phylogenetic tree of 31 *O*. *sinensis* accessions constructed using SNP data. The scale bar represents the evolutionary distances measured by *p*-distance. (**B**) Two-way principal components analysis (PCA) using identified SNPs. The leading five eigenvectors explain 71.4% variance with 34.5% by eigenvector 1 and 20.1% for eigenvector 2 (Supplemental Figure S20A). (**C**) Population structure of *O*. *sinensis*. Each color and vertical bar represents one population and one accession, respectively. The y-axis shows the proportion of each accession contributed from ancestral populations. (**D**) Latitude distribution for the three inferred groups (G1, G2 and G3). The longitude, latitude and altitude for each population were determined by GPS when collecting samples in the field (Supplemental Table S37). (**E**) Nucleotide diversity and population divergence across the three groups. Values in parentheses indicate the nucleotide diversity (π) for groups, while values between pairs represent the population divergence measured by *F*_*ST*_. (**F**) Geographic distribution of the 31*O*. *sinensis* populations and their latitude-based divergence. The maps are generated using ArcGIS software (version 10.1; https://www.arcgis.com/features/index.html).

To examine the genome-wide relationships and population divergence, we collected and resequenced 31 accessions of *O*. *sinensis* across its known distribution range, including the Qinghai, Sichuan, Yunnan and Gansu provinces and the Tibet Autonomous Region on the Qinghai-Tibetan Plateau (Supplemental Figure S16 and Supplemental Table S37). We generated a total of 183 million paired-end reads (~36.68 Gb of sequences) with an average depth of ~10.1× (raw data) (Supplemental Table S38). From these data, we generated a set of 816,960 single-nucleotide polymorphisms (SNPs) and 48,092 strict indels (insertions and deletions) to assess relatedness among populations of *O*. *sinensis* (Supplemental Figures S18–19 and Supplemental Table S39). The majority of genomic variants (71.1%) were mapped to intergenic regions with a subset mapping to the coding regions (23.3% consisting of 101,997 synonymous and 88,224 nonsynonymous SNPs with the substitution ration of 0.86) (Supplemental Figure S19 and Supplemental Table S39). The phylogenetic tree constructed using the SNP datasets divided the 31 accessions into three geographically separate groups ranging from low-latitude to high-latitude regions (Fig. 4A)—a finding that was reinforced by PCA using the first and second eigenvectors (Fig. 4B and Supplemental Figure S20A). Varying the number of presumed ancestral populations (*K*) showed that when *K* = 3, the three distinct groups are consistent with the PCA and phylogenetic reconstruction (Fig. 4C and Supplemental Figure S20B). Some accessions from the low-latitude group exhibit strong evidence of the admixture and are more dispersed compared with other two groups, indicating greater genetic diversity possibly due to the shared ancestral polymorphisms and/or recent introgression events (Fig. 4D,E). The estimated population-differentiation statistic (*F*_*ST*_) among these three latitude-based groups further revealed the basal nature of the low-latitude region, particularly populations from the Nyingchi District of Tibet, which was further evidenced by its substantially elevated nucleotide diversity (π) within the group and lowered population differentiation with the other two high-latitude groups. (Fig. 4C–F).

We further investigated the genes affected by different levels of SNP contents and non-synonymous mutations (Supplemental Table S40). Functional enrichment analysis of the top 100 genes with the highest SNP content and/or non-synonymous mutations shows that they are mainly involved in the metabolism of fungal secondary metabolites, such as polyketide synthase dehydratase (PF14765; FDR < 0.01), KR domain (PF08659; FDR < 0.01) and condensation domain (PF00668; FDR < 0.01). The functional categories associated with fatty acid biosynthesis were also enriched, such as acyl transferase domain (PF00698; FDR < 0.01) and beta-ketoacyl synthase (PF00109 and PF02801; FDR < 0.01) (Supplementary Tables S41–42).

## Discussion

We report here the ~116 Mb repeat-rich (80.07%) high-quality genome of *O*. *sinensis*–a fungal species endemic to the Qinghai-Tibetan Plateau. Compared with other related entomopathogenic fungal species in the Hypocreales family, we amazingly found that rapid amplification of LTR retrotransposons with one estimated peak of retrotransposion activities approximately ~38 MYA, which have greatly contributed to about 3.4-fold inflation of the *O*. *sinensis* genome. Resequencing and assembling the GS05 genome of *O*. *sinensis* from the populations that reside at a low altitude also annotated a high proportion of LTR retrotransposons (Supplementary Tables S43–44). Interestingly, the timing of the proliferation of LTR retrotransposons in *O*. *sinensis* coincides with the uplift of the Qinghai-Tibetan Plateau^36, 37^. However, it is unclear whether these two events occurred coincidentally or not, and further efforts are needed to ensure this finding. Comparative genomic analyses between this species and other entomopathogenic fungi further revealed an immense loss of protein-coding genes, of which non-collinear genes that mainly relate to amino acid metabolism were massively removed in *O*. *sinensis* in comparison with *C*. *militaris*, which is native to low altitudes. Native fungi such as *O*. *sinensis* on the plateau that have survived over thousands of years must have reduced genomic structural complexity and thus genetically developed adaptive mechanisms to address harsh environmental stresses during the formation of the higher mountains of the Himalayas. Nevertheless, we still lack molecular and evolutionary evidence and need clues about how the massive removal of non-collinear genes and many rounds of retrotransposition occurred, responding to high-altitude environmental stresses.

The analysis of the *O*. *sinensis* genome revealed that gene families related to fungal pathogenicity have experienced rapid evolution and undergone natural selective pressures. Compared with other entomopathogenic fungi, we apparently observed a global contraction of gene families in this highland fungal species, evidenced by the removal of 2,468 non-collinear genes and a total loss of 1,756 gene families from the most recent common ancestor. It is apparent that the adaptation to an extremely specialized environment and/or narrow host range of *O*. *sinensis* may have driven this gene loss as observed in the ectomycorrhizal symbiont *T*. *melanosporum* ^23^. Functional properties of these gene families exhibiting contraction status are enriched in the transport process and energy metabolism, suggesting that *O*. *sinensis* might reduce the transportation of materials outside membrane to depress energy consumption to adapt to extremely harsh environments. In contrast, the *O*. *sinensis* genome displayed considerable expansion of gene families that are mainly involved in fungal pathogenicity, including peroxidase activity^27, 38^, serine hydrolase^39^, deuterolysin metalloprotease (M35) peptidase^40, 41^ and cytochrome P450^42, 43^. We also found that gene families functionally related to the adaptability of low-temperature were over-represented, probably enabling *O*. *sinensis* to gain an enhanced cold- tolerance to adapt to extremely low- temperature environmental challenges at high altitudes. Genes specific to *O*. *sinensis* were significantly enriched in GO categories associated with starch binding, pathogenesis and cell wall macromolecule catabolic process. Such an expansion of lineage-specific gene families may serve as candidates for adaptive evolution of fungal pathogenicity in *O*. *sinensis*. It was worth noting that, besides extraordinary expansion of a large number of genes associated with fungal pathogenicity, positive selection also acts on these genes in *O*. *sinensis* and may have contributed to its specialized host infection mechanism.

*O*. *sinensis* provides an unprecedented opportunity to study adaptive evolution of gene families that are associated with specific physical characteristics or adaptive traits in the context of convergent evolution of fungi with high-altitude insects. Insect molting is one of the most important developmental stages, which is mainly determined by steroidal molting hormones (ecdysteroids). It was previously reported that the host insect of *O*. *sinensis* is vulnerable to be infected by fungi during late summer when the host insect sheds its cuticles^44^. The expanded genes enriched in the Pfam category of GMC oxidoreductase involved in the ecdysteroid metabolism of insect molting may have potential relationship with the specific fungal pathogenicity mechanism in *O*. *sinensis* ^45, 46^. To successfully complete the infection of host cells, pathogens must be equipped with means to effectively incapacitate production of host-driven ROS or detoxify ROS. Several recent studies proposed that peroxidases, acting as ROS-scavenging enzymes, are integral components of the antioxidant defense system in diverse pathogens^27, 38, 47^. Our analysis revealed a remarkable expansion of peroxidase genes in *O*. *sinensis* compared with other closely related fungal species that reside at low latitudes. *O*. *sinensis* often occurs at high altitudes of the Qinghai-Tibetan Plateau under intensive solar ultraviolet (UV) radiation, which may promote the generation of ROS and increase both the intracellular and extracellular ROS concentrations. Thus, the increase of peroxidase genes in *O*. *sinensis* may have greatly contributed to the strong ROS detoxification for host infection in *O*. *sinensis*. It is not surprising that *O*. *sinensis* and other insect pathogens generally had more proteases and protein kinases to degrade the insect cuticle in comparisons to plant pathogens. Although *O*. *sinensis* alone had remarkably fewer genes encoding proteases than other entomopathogenic fungi, approximately 35% of them detected in *O*. *sinensis* have a signal peptide, indicating a more efficient usage of secreted proteases in *O*. *sinensis* to adapt to more specific insect cuticles.

Fungi have long been regarded as an excellent model to understand the evolution of the mating system largely owing to their extensive ecological adaptation and evolutionary diversification. A previous study reported that *O*. *sinensis* harbored the *MAT1-2-1* mating-type gene within the *MAT1-2* idiomorph, but they unfortunately failed to detect any fragments in the *MAT1-1* idiomorph, making its mating system and sexual cycle incompletely understood^48^. Our analysis of the *O*. *sinensis* genome detected both *MAT1-1* and *MAT1-2*, which were further confirmed by using whole-genome resequencing of 31 representative populations. The results demonstrate that *O*. *sinensis* is indeed homothallic, which is in sharp contrast to the closely related heterothallic insect pathogens with a single mating-type locus^19–22^. Former phylogenetic analysis confirmed that multiple evolutionary transitions from out-crossing to inbreeding have occurred during the evolutionary history of insect pathogens, of which homothallic *O*. *sinensis* diverged from the heterothallic *Cordyceps* spp.^15^. Such a specialized self-fertility life cycle of *O*. *sinensis* is likely to be a niche adaptation for the survival of small effective populations under extreme environmental conditions on the Qinghai-Tibetan Plateau.

Resequencing multiple individuals from natural populations representing the range of *O*. *sinensis* provided in-depth insights into evolutionary scenarios occurring under natural selection for success at high latitudes on the Qinghai-Tibetan Plateau. We found that the genomes from these populations are genetically distinguishable, which form the three latitude-based distinct groups ranging from low-latitude to high-latitude regions. Low-latitude populations exhibited two-fold higher levels of genomic diversity and admixture with populations from the other two high-latitude groups, suggesting that they may act as source populations to disperse and populate at elevated habitats in the course of population evolution. This study thus highlights geographically demographic history based on latitudes and nature selection in *O*. *sinensis* for adaptation to high altitudes. However, further studies are needed to answer how selected traits have contributed to facilitating rapid population adaptation, how the mating system varies and evolves among populations, and particularly how this fungal pathogen coevolves with insects at different latitude regimes on the Qinghai-Tibetan Plateau.

To conclude, the high-quality reference genome sequence together with other data from this study pave the way for understanding genetic basis underlying fungal pathogenicity and coevolution of fungi- insects at high- altitudes, enhancing germplasm conservation and sustainable utilization and accelerating commercial cultivation and genetic improvement of *O*. *sinensis* to meet increasing global demand for this precious natural traditional Chinese medicine.

## Methods

### Genome sequencing, assembly, and validation

We sequenced the *O*. *sinensis* genome by using a combination of Illumina HiSeq 2000 and Roche 454 sequencing technologies. The obtained reads were assembled using Newbler^16^ and SSPACE^17^, yielding a high-quality genome assembly. We assessed the quality of the assembled genome by using four evaluation methods. Firstly, we aligned all DNA and EST sequences of *O*. *sinensis* available in public databases to the final genome assembly and calculated mapping rates. Secondly, we mapped all clean sequencing reads to the assembled genome sequences and statistically examined mapping rates of these reads to the whole genome. Third, we assembled RNA sequencing data of *O*. *sinensis* generated in this study into transcripts using Trinity, and then further evaluated the assembly quality by aligning these assembled transcripts against the sequences from the final assembly of *O*. *sinensis* genome. Finally, we mapped the﻿ BUSCO conserved genes from Ascomycota lineage to our genome assembly and then analyzed the mapping result.

### Genome annotation

Putative protein-coding genes of *O*. *sinensis* were predicted by combining several different *ab initio* gene predictors and sequence evidences that includes protein sequences from closely related species and ESTs assembled in this study. Quality validation of gene models was evaluated by aligning transcriptome, Ascomycota BUSCOs, and homologous peptide to our gene predictions. Annotation of TEs were performed by integrating RepeatMasker (www.repeatmasker.org), LTR_STRUCT^49^, RECON^50^, and LTR_Finder^51^. The four different types of noncoding RNA genes, including tRNA, rRNA, snRNA and snoRNA, were predicted using *de novo* and homology search methods.

### Data access

The sequencing reads have been deposited in the National Center for Biotechnology Information database (www.ncbi.nlm.nih.gov), and can be freely accessed under the BioProject number of PRJNA382001. Genome assembly, gene prediction, gene functional annotations, and transcriptomic﻿ data may be accessed via the web site at: http://www.plantkingdomgdb.com/Ophiocordyceps_sinensis/.

## Electronic supplementary material

Supplementary Information

Supplementary Large Tables
