# Supplementary material for: The caterpillar fungus, Ophiocordyceps sinensis, genome provides insights into highland adaptation of fungal pathogenicity
Source: Sci Rep. 2017 May 11;7:1806. doi: 10.1038/s41598-017-01869-z (PMC5432009; doi:10.1038/s41598-017-01869-z)
Supplement: Supplementary file 1 — Supplementary Information [file 41598_2017_1869_MOESM1_ESM.pdf]

Supporting Information Appendix

The caterpillar fungus, *Ophiocordyceps sinensis*, genome provides insights into highland adaptation of fungal pathogenicity

En-Hua Xia[1, 3*], Da-Rong Yang[2*], Jian-Jun Jiang[1*], Qun-Jie Zhang[4], Yuan Liu[1], Yun-Long Liu[1], Yun Zhang[1], Hai-Bin Zhang[1], Cong Shi[1, 3], Yan Tong[1], Changhoon Kim[5], Hua Chen[6], Yan-Qiong Peng[2], Yue Yu[5], Wei Zhang[5], Evan E. Eichler[7, 8] and Li-Zhi Gao[1]†

[1] Institution of Genomics and Bioinformatics, South China Agricultural University, Guangzhou 510642, China

[2] Xishuangbanna Tropical Botanical Garden, the Chinese Academy of Sciences, Menglun 666303, China

[3] University of the Chinese Academy of Sciences, Beijing 100039, China

[4] Agrobiological Gene Research Center, Guangdong Academy of Agricultural Sciences, Guangzhou 510640, China

[5] Marcogene Inc., Seoul 08511, South Korea

[6] Center for Computational Genomics, Beijing Institute of Genomics, the Chinese Academy of Sciences, Beijing 100101, China

[7] Department of Genome Sciences, University of Washington School of Medicine, Seattle, WA 98195, USA

[8] Howard Hughes Medical Institute, University of Washington, Seattle, WA 98195, USA

*These authors contributed equally to this work;

†To whom correspondence should be addressed: Tel/Fax: +86-871-65223277; Email: Lgao@mail.kib.ac.cn

Table of contents

Supplementary section S1 - Genome sequencing, assembly and validation

1.1 Fungal materials

1.2 DNA library construction and genome sequencing

1.3 Reads quality check and filtering

1.4 Estimation of genome size

1.4.1 Estimation of genome size using *k*-mer method

1.4.2 Estimation of genome size using flow cytometry

1.5 Genome assembly

1.6 Assessment of assembly quality

Supplementary section S2 - Genome Annotation

2.1 Transcriptome sequencing

2.1.1 Collection of fungal materials at different developmental stages

2.1.2 RNA isolation, sequencing and assembly

2.2 Annotation of repeat sequences

2.2.1 Identification of known transposable elements (TEs)

2.2.2 Identification of *de novo* repeats

2.2.3 Tandem repeats prediction

2.2.4 TE insertion timing

2.3 Annotation of protein-coding genes and quality validation

2.3.1 Protein-coding gene prediction

2.3.2 Gene function annotation

2.3.3 Quality validation of gene models

2.4 Annotation of non-coding RNA genes

2.5 Identification of simple sequence repeat (SSR)

Supplementary section S3 - Comparative analysis of *O. sinensis* and *C. militaris* genomes

3.1 Identification of collinear blocks between *O. sinensis* and *C. militaris*

3.2 Classification of non-collinear genes within blocks

Supplementary section S4 – Dynamic evolution of gene families

4.1 Identification of gene families

4.2 Expansion and contraction of gene families

4.3 Species-specific gene families

4.4 Identification of peroxidase gene family

4.5 Identification of proteases, kinases and transporters

4.6 Identification of Carbohydrate-Active Enzymes (CAZymes)

4.7 Differential expression genes among the three developmental stages

Supplementary section S5 – Phylogenetic analysis and positive selection test

5.1 Resolution of phylogenetic relationships among the thirteen fungi with fully sequenced genomes

5.2 Speciation timing

5.3 Identification of positively selected genes

Supplementary section S6 – Analysis of mating type genes

6.1 Identification of mating-type (MAT) genes

6.2 Verification using resequencing data

6.2 Syntenic analysis of MAT loci between *O. sinensis* and *C. militaris*

Supplementary section S7 –Genome resequencing and population genetic analysis

7.1 Sample collection and genome resequencing

7.2 Reads filtering, mapping and SNP calling

7.3 Population genetic analyses

7.4 Assessment of nucleotide diversity and population differentiation

Supplementary URLs

References

Supplementary Figures

Supplementary Tables

Supplementary section S1 - Genome sequencing, assembly and validation

1.1 Fungal materials

A wild *O. sinensis* individual was harvested from Nyingchi of Tibet Autonomous Region, China with an altitude above 4000 m. The material was taxonomically identified by Prof. Da-Rong Yang, who is expert in studies on *O. sinensis* at Xishuangbanna Tropical Botanical Garden, the Chinese Academy of Sciences. After collection, the sample was immediately frozen in liquid nitrogen and then stored at -80°C in the laboratory prior to DNA extraction.

1.2 DNA library construction and genome sequencing

High-quality genomic DNA was extracted from the fruiting body using a modified CTAB method (Porebski et al. 1997). RNase A and proteinase K were separately used to remove RNA and protein contamination. The quantity and quality of the isolated DNA were separately checked by electrophoresis on a 0.8% agarose gel and a Nanodrop D-1000 spectrophotometer (NanoDrop Technologies, Wilmington, Delaware). Four 454 and two Illumina mate-pair genomic sequencing libraries were separately constructed following the Roche and Illumina instructions, and subsequently sequenced using Roche 454 GS FLX and Illumina Hiseq2000 platforms, respectively. In total, we generated approximate 8.96 Gb of raw sequencing read data with length varying from 101 to 703 bp (**Table S1**).

1.3 Reads quality check and filtering

To remove the potential sequencing errors and adapter contamination, we filtered out the following types of reads: 1) Reads with unidentified nucleotides (Ns) ≥ 10% of its length; 2) Reads with ≥ 10 bp from the adapter sequence (allowing ≤ 10% mismatches); 3) Reads with ≥ 40% low quality bases (Phred score ≤ 5); 4) Reads caused by PCR duplications (i.e. Read 1 and Read 2 of two paired-end reads that were

completely identical); 5) Reads with the *k*-mer frequency ≤ 3 (aiming to minimize the influences of sequencing errors). Finally, a total of ~5.41 Gb (~45.1× coverage) high-quality data was retained and used for subsequent analysis (**Table S1**).

1.4 Estimation of genome size

The genome size of *O. sinensis* was investigated by two widely used methods including *k*-mer frequency distribution and flow cytometric analysis.

1.4.1 Estimation of genome size using *k*-mer method

Estimation of genome size using *k*-mer frequencies of sequencing reads has been broadly and successfully applied in diverse organisms (Li et al. 2013; Zheng et al. 2013; Zhang et al. 2014). To estimate the genome size of *O. sinensis*, we here generated the 17-mer occurrence distribution of *O. sinensis* using jellyfish (version 2.1.3) (Marcais and Kingsford 2011) and then calculated the genome size based on the formula of $G = \frac{N \times (L - K + 1)}{L \times D}$. Here, G represents the genome size; N is the total number of reads used; L is the average length of reads; *K* equals to 17 as used by jellyfish; D indicates the depth of sequencing data. According to this formula, the genome size of *O. sinensis* was finally estimated to be ~119.8 Mb (**Fig. S1 and Table S2**).

1.4.2 Estimation of genome size using flow cytometry

The genome size of *O. sinensis* was further estimated and validated using flow cytometry analysis. We used approximate 40-50 mg of fruiting body for sample preparation. The nuclei suspensions was organized in Otto buffer and analyzed using a BD FACSCalibur (USA) flow cytometer. Results were acquired using CellQuest software (version 5.1).

We used rice (*Oryza sativa* ssp. *japonica* cv. Nipponbare) as inner standard and estimated the genome size of *O. sinensis* based on the linear relationship of 2C peaks. As the equation of 1 pg DNA = 978 Mb, the *O. sinensis* genome was approximately

estimated to be 124.08 Mb, which is quite consistent to the *k*-mer analysis above (**Fig. S2 and Table S3**).

1.5 Genome assembly

We assembled the genome sequences of *O. sinensis* using a combined methods of Newbler (Margulies et al. 2005) and SSPACE (Boetzer et al. 2011).

Firstly, the high-quality 454 reads were used to construct contig sequences by Newbler (version 2.8) (Margulies et al. 2005). This step resulted in a total of 9,619 contigs with a CtgN50 value of 21,393 bp and total length of 112,056,267 bp. Secondly, all the mate-pair reads from long-insert libraries were aligned onto the pre-assembled contigs. Based on the distance information, the order, distance and orientation of the contigs were assessed and eventually combined into scaffolds using SSPACE (Boetzer et al. 2011). To close the gaps that might be repeat sequences masked during the construction of scaffolds, we used the aligned paired-end information to retrieve the read pairs with one read well-aligned on the contigs and another located in the gap region, and then performed a local assembly for these collected reads using the package GapCloser (version 1.12; **Supplementary URLs**).

Consequently, we generated a total of ~116.42 Mb sequences for the final *O. sinensis* genome assembly. The CtgN50 and ScfN50 were 21,423 bp and 2,999,605 bp, respectively. Totally 156 scaffolds have its length > 2 Kb, and among them, the largest scaffold had a length of 9,561,753 bp (**Table S2**).

1.6 Assessment of assembly quality

To assess the quality of genome assembly, we adopted four evaluated approaches. First, a total of 10,603 DNA and 16,676 EST sequences were separately downloaded from National Center for Biotechnology Information (NCBI) database (as of March 2015) using the key words of “txid72228 [Organism: exp]”. We then mapped all downloaded DNA and EST sequences to our genome assembly using GMAP (version 2014-10-22). Results showed that nearly 10,481 and 15,897 of them could be well aligned, showing mapping rates of 98.85% and 95.33%, respectively (**Table S5**).

Second, all clean 454 sequencing reads were mapped back to our genome assembly using Newbler (version 2.8). Results revealed that nearly 99.01% of them have a good alignment with our genome assembly (**Table S5**). Third, we assembled the RNA sequencing reads of *O. sinensis* generated in this study into transcripts using Trinity (version v2.0.6), and then aligned them back to our genome assembly using GMAP (version 2014-10-22). Of the 11,742 transcripts assembled, we observed that approximately 11,064 (~94.23%) had matches with our genome assembly (**Table S5**). Fourth, we further evaluated the completeness of our *O. sinensis* assembly using BUSCO (Simao et al. 2015). Our results show that 94 and 4 % of the 1,315 expected Ascomycota BUSCO conserved genes were identified as complete and fragmented, respectively, and only 1.8 % were considered as missing in the genome assembly (**Table S5**). Taken together, our DNA, EST, transcriptome, reads mapping and BUSCOs alignments verified a high-quality of the genome assembly, ensuring subsequent comparative genomic analyses in this study.

Supplementary section S2 - Genome Annotation

2.1 Transcriptome sequencing

2.1.1 Collection of fungal materials at different developmental stages

A total of six individuals representing three developmental stages of *O. sinensis* were harvested from Deqin County of Yunnan Province, China. The average length ratio for fungus/insect of them ranged from ~ 1.2 to 2.2 × (**Table S6 and Fig. S4**). After collection, these samples were immediately frozen in liquid nitrogen and stored in laboratory at -80℃ refrigerator until RNA extraction.

2.1.2 RNA isolation, sequencing and assembly

RNA was extracted from the fruiting bodies by using RNeasy Plant Mini Kit (QIAGEN). RNase-free DNAse I (Takara) was used to remove residual DNA. Quality and integrity of the RNA samples were separately surveyed using NanoDrop-1000 UV-VIS spectrophotometer (NanoDrop) and Agilent 2100 Bioanalyzer (Agilent Technologies, Palo Alto, CA, USA). For each sample, at least 20 μg of the total RNA with a concentration of ≥ 400 ng/μl was used for constructing cDNA library according to the manufacturer's recommendations (Illumina, USA). The resulting libraries were finally sequenced using Illumina Hiseq2000 platform. In total, approximately 15.05 Gb raw data with a read length of 101 bp were obtained (**Table S6**).

Raw reads were processed following a quality-control procedure that was used for the genome assembly. The duplicate reads caused by PCR amplification were also filtered. As a result, a total of ~14.72 Gb clean data were retained and used for subsequent assembly (**Table S6**). We assembled the transcriptome of *O. sinensis* using Trinity (version r20140717) (Grabherr et al. 2011) with "*--jaccard_clip*" option due to the high gene density of fungal species. Results showed that the assembled transcriptome of *O. sinensis* harbored a total of 11,742 unigenes with a N50 value of 3,793 bp and a total length of ~21.69 Mb (**Table S7**).

2.2 Annotation of repeat sequences

We systematically annotated repeat sequences for the *O. sinensis* genome using the following steps:

2.2.1 Identification of known transposable elements (TEs)

To identify the known TEs within *O. sinensis* genome, RepeatMakser (version 4.0.5) (**Supplementary URLs**) with the RepBase TE library (version 20140131) (Jurka 2000; Jurka et al. 2005) was used. We also performed RepeatProteinMask (**Supplementary URLs**) searches with the TE protein database as a query library.

2.2.2 Identification of *de novo* repeats

The *de novo* repeat library for *O. sinensis* was constructed using RepeatModeler (**Supplementary URLs**). This package can automatically execute two core *de novo* repeat finding programs, including RECON (version 1.08) (Bao and Eddy 2002) and RepeatScout (version 1.0.5) (Price et al. 2005), to finally build, refine and classify consensus models of putative interspersed repeats for the *O. sinensis* genome.

2.2.3 Tandem repeats prediction

We identified tandem repeats within the *O. sinensis* genome using Tandem Repeat Finder (TRF) package (version 4.04) (Benson 1999) with the parameters of "Match=2, Mismatch=7, Delta=7, PM=80, PI=10, Minscore=50, and MaxPeriod=12". The non-interspersed repeat sequences, including low complexity repeat, satellites and simple repeats, were also identified using RepeatMasker (version 4.0.5) (**Supplementary URLs**) with parameter of "-noint".

2.2.4 TE insertion timing

For the conversion of the nucleotide sequence distance to putative genome insertion age, a substitution rate of 1.05×10^{-9} mutations per site per year was used (Kasuga et al. 2002; Berbee and Taylor 2010; Rouxel et al. 2011). The insertion time was counted

by T = *K*/2r. T: element insertion time; r: synonymous mutation/site/year; *K*: the divergence between the LTRs and consensus sequence in the TE library.

2.3 Annotation of protein-coding genes and quality validation

2.3.1 Protein-coding gene prediction

We predicted the protein-coding genes of *O. sinensis* using three methods including *ab initio* gene prediction, homology-based gene prediction and RNA-Seq aided gene prediction. Prior to gene prediction, the assembled *O. sinensis* genome was hardly and softly masked by RepeatMasker (version 4.0.5), respectively.

2.3.1.1 *ab initio* gene prediction

The *ab initio* gene prediction was performed using Augustus (version 3.0.3) (Stanke et al. 2004; Stanke and Morgenstern 2005; Stanke et al. 2006) and SNAP (version 2006-07-28) (Korf 2004) package. Models used for gene annotation were trained from a set of high-quality proteins generated using RNA-Seq data (**Supplementary section 2.1.2**).

2.3.1.2 Homology-based gene prediction

For homology-based gene prediction, the peptides from the five closely related fungal species including *T. inflatum* (Bushley et al. 2013), *C. militaris* (Zheng et al. 2011), *B. bassiana* (Xiao et al. 2012), *M. anisopliae* (Gao et al. 2011) and *M. acridum* (Gao et al. 2011) were downloaded from DOE Joint Genome Institute's (JGI) Genome Portal (Grigoriev et al. 2012; Grigoriev et al. 2014; Nordberg et al. 2014) (**Supplementary URLs**) and then aligned to the *O. sinensis* genome assembly using exonerate (version 2.2.0) (Slater and Birney 2005) (**Table S17**). Given the fact that fungal species usually possessed a short intron length distribution, here the allowed minimum and maximum intron lengths of *O. sinensis* to make alignment were set to 10 bp and 1,000 bp, respectively.

We also mapped the protein sequences of *T. inflatum*, *C. militaris*, *B. bassiana*, *M. anisopliae* and *M. acridum* to the *O. sinensis* genome assembly using genBlastA

(version 1.0.1) (She et al. 2009) with the parameters of "-e 1e-2 -d 1000". Then homologous genomic fragments of the target genes together with 2000 bp at both sides were extracted and aligned against matching proteins using GeneWise (version 2.2.0) (Birney and Durbin 2000; Birney et al. 2004) to determine the gene structure.

2.3.1.3 RNA-Seq aided prediction

For RNA-Seq aided gene prediction, the RNA-seq sequencing reads were first assembled into 11,742 transcripts using Trinity (version r20140717) (Grabherr et al. 2011) (**Table S7 and Supplementary section 2.1.2**), and then the PASA (Program to Assemble Spliced Alignments; **Supplementary URLs**) (Haas et al. 2003) alignment was performed. The output included a set of consistent and non-overlapping sequence assemblies to describe gene structures.

2.3.1.4 EvidenceModeler merging and further filtering

All gene structures from the above three predictions, including *ab intio* gene predictions and protein and transcript alignments, were finally combined into weighted consensus gene set of *O. sinensis* using EVidenceModeler (EVM; **Supplementary URLs**) (version 1.1.1) (Haas et al. 2008). Genes with their peptide lengths shorter than 50 amino acids (aa) and/or harboring inner stop codons were removed. Consequently, we identified a total of 7,939 protein-coding genes in *O. sinensis* (**Table S8**).

2.3.2 Gene function annotation

Gene functions were assigned according to the best match of the alignment to the Swiss-Prot and TrEMBL databases using BLASTP (version 2.2.26) (Altschul et al. 1997; Camacho et al. 2009) with the e-value threshold of $1e^{-5}$. The motifs and domains within gene models were identified by InterProScan (version 5.3.46) (Zdobnov and Apweiler 2001; Quevillon et al. 2005; Jones et al. 2014) against multiple publicly available databases, and the Gene Ontology IDs for each gene were directly obtained from the corresponding InterPro entry. In total, approximately 97%

of the *O. sinensis* genome could be functionally annotated with known genes, conserved domains, or Gene Ontology (GO) terms (**Table S9**).

2.3.3 Quality validation of gene models

To validate the quality of gene predictions, we first compared the length distribution of protein-coding genes, coding sequences (CDS), exons and introns between *O. sinensis* and closely related fungal species including *C. militaris*, *B. bassiana*, *M. anisopliae* and *M. acridum*. Results showed that there were no obvious differences of gene features, indicating high quality of gene structure prediction for *O. sinensis* (**Fig. S5**).

We also examined the proportion of *O. sinensis* gene set supported by RNA-Seq and/or homologous proteins. Firstly, RNA-Seq data of *O. siensis* were assembled into transcripts using Trinity (**Supplementary section 2.1.2**), and then BLAST (version 2.2.26) was used to align them to the gene models predicted. Hits with identity ≥ 90% and coverage of gene model ≥ 80% were retained. In total, nearly 71.51% (5,677) of predicted genes could be supported by RNA-Seq data (**Table S11**). Secondly, protein sequences from the four closely related fungal species including *C. militaris*, *B. bassiana*, *M. anisopliae* and *M. acridum* were download from DOE Joint Genome Institute's (JGI) Genome Portal (**Table S17 and Supplementary URLs**), and then aligned to the predicted *O. sinensis* gene models using BLAST (version 2.2.26). We filtered those hits with an identity < 30% or coverage of gene models < 80%, and found 6,441 gene models, accounting for approximately 81.13% of the total genes, could be supported with the evidence of homologous proteins (**Table S11**). Taken together, we showed that a total of 7,173 genes, representing ~90.35% of *O. sinensis* gene set, could be supported by RNA-Seq or homologous proteins from its closely related fungal species (**Table S11**). Using the BUSCO from Ascomycota lineage, we further showed that 94.4 % of the genes as complete, 3.6 % fragmented, 1.8 % missing, and 0.2% duplicated, indicating a good quality of our gene prediction (**Table S11**).

2.4 Annotation of non-coding RNA genes

The four different types of non-coding RNA genes, namely transfer RNA (tRNA) genes, ribosomal RNA (rRNA) genes, small nucleolar RNA (snoRNAs) genes and small nuclear RNA (snRNAs) genes, were predicted using *de novo* and homology search methods. We used tRNAscan-SE algorithms (version 1.23) with default parameters (Lowe and Eddy 1997) to identify the tRNA genes, which is an adaptor molecule composed of RNA used in biology to bridge the three-letter genetic code in messenger RNA (mRNA) with the twenty-letter code of amino acides in proteins. We totally annotated 146 tRNA genes in the *O. sinensis* genome (**Table S12**). The rRNA genes (8S, 18S, and 28S), which is the RNA component of the ribosome, the enzyme that is the site of protein synthesis in all living cells, were predicted by using RNAmmer algorithms with default parameters (Lagesen, Hallin et al. 2007). Here we totally annotated 33 rRNA genes in the *O. sinensis* genome (**Table S12**). snoRNAs are a class of small RNA molecules that guide chemical modifications of other RNAs, mainly ribosomal RNAs, transfer RNAs and small nuclear RNAs. The snoRNA genes were annotated using snoScan with the yeast rRNA methylation sites and yeast rRNA sequences provided by the snoScan distribution (Lowe and Eddy 1999). In total, we annotated 70 snoRNA genes in the *O. sinensis* genome (**Table S12**). snRNA is a class of small RNA molecules that are found within the nucleus of eukaryotic cells. They are involved in a variety of important processes such as RNA splicing (removal of intron from hnRNA), regulation of transcription factors (7SK RNA) or RNA polymerase II (B2 RNA), and maintaining the telomeres. The snRNA genes were identified by INFERNAL software against the Rfam database (release 9.1) with default parameters (Griffiths-Jones, Moxon et al. 2005; Nawrocki, Kolbe et al. 2009). Totally, we annotated 15 snRNA genes in the *O. sinensis genome* (**Table S12**).

2.5 Identification of simple sequence repeat (SSR)

Six types of SSRs from mono- to hexa-nucleotides were identified using the MISA Perl script (http://pgrc.ipk-gatersleben.de/misa/). The minimum repeat unit size for mono- nucleotides was set at ten and six for di-, and at five for tri- to hexa-nucleotides.

As a result, a total of 8,918 SSRs from 199 assembled scaffolds were detected. Of these 8,918 SSRs, tri-nucleotide repeats were the most abundant type (3,157; 35.4%), followed by mono-nucleotides (2,777; 31.1%), di-nucleotides (2,590; 29.0%), hexa-nucleotides (187; 2.1%), penta-nucleotides (136; 1.5%), and tetra-nucleotides (71; 0.8%) (**Table S15-16 and Fig. S7**).

Supplementary section S3 – Comparative analysis of *O. sinensis* and *C. militaris* genomes

3.1 Identification of collinear blocks between *O. sinensis* and *C. militaris* genomes

We adopted the MCScanX package (Wang et al. 2012) to generate the collinear blocks between *O. sinensis* and *C. militaris*. Briefly, a total of 9,684 protein sequences of *C. militaris* together with its genomic annotation gff3 file were downloaded from JGI Genome Portal (**Table S17**). BLASTP was then used to align them to the gene models of *O. sinensis* with an e-value of $1e^{-10}$. To make MCScanX (Wang et al. 2012) produce reasonable results, the number of hits from BLASTP for each gene was set to 5 as suggested by MCScanX (Wang et al. 2012) manual. Based on the gene positions shown in the gff3 files, MCScanX (Wang et al. 2012) was finally used to detect the collinear blocks between *O. sinensis* and *C. militaris* following the criteria of a) the number of genes required to call a collinear block should be ≥ 5; b) number of non-syntenic genes to collapse the collinear blocks should be ≥ 5.

Consequently, a total of 308 collinear blocks consisting of 7,984 (45.39%) collinear genes were identified between *O. sinensis* and *C. militaris* (**Table S18 and Fig. S10**).

3.2 Classification of non-collinear genes within blocks

A total of 3,259 (33.8%) and 2,127 (26.8%) non-collinear genes for *C. militaris* and *O. sinensis* were identified in 308 collinear blocks, respectively (**Table S19**). To investigate their homology relationships, we performed a clustering analysis using OrthoMCL (version 2.0.9) (Li et al. 2003; Chen et al. 2006) based on all-*vs*-all BLAST searches. Results showed that there were 1,832 genes shared between *C. militaris* and *O. sinensis* (**Fig. S11**). Of them, 1,041 genes and 791 genes separately came from *O. sinensis* and *C. militaris*. We also detected 2,468 and 1,086 genes that specifically belong to *C. militaris* and *O. sinensis*, respectively. To functionally

annotate these genes, Kyoto Encyclopedia of Genes and Genomes (KEGG) terms for each gene were retrieved from InterProScan entry (**Table S20**) and then summarized.

Supplementary section S4 – Dynamic evolution of gene families

4.1 Identification of gene families

We identified gene families (clusters) among 13 fungal species including 5 insect infection fungi (*T. inflatum, M. anisopliae, M. acridum, C. militaris* and *B. bassiana*), 6 plant infection fungi including *Fusarium graminearum* (Cuomo et al. 2007), *Magnaporthe grisea* (Dean et al. 2005), *Grosmannia clavigera* (DiGuistini et al. 2011), *Sclerotinia sclerotiorum* (Amselem et al. 2011), *Botrytis cinerea* (Amselem et al. 2011) and *Verticillium alfalfae* (Klosterman et al. 2011), *Saccharomyces cerevisiae* (Goffeau et al. 1996) (**Supplementary URLs**) and *O. sinensis* using OrthoMCL package (version 2.0.9) (Li et al. 2003; Chen et al. 2006). Briefly, protein sequences from these 13 species except for *O. sinensis* were first downloaded from public databases and only the longest non-TE isoforms for each genes were retained (**Table S17**). Then, an all-*vs*-all comparison was performed using BLASTP (version 2.2.26) (Altschul et al. 1997; Camacho et al. 2009) with an e-value threshold of 1e-5. Finally, the clustering was conducted using Markov cluster algorithm (MCL) (Enright et al. 2002; van Dongen and Abreu-Goodger 2012) integrated in the OrthoMCL package (version 2.0.9). The inflation value (-I) of 1.5 for Markov cluster algorithm (MCL) was adopted. Consequently, we totally identified 14,706 gene families among these 13 fungal species. The family size for each species ranged from 1.07 (*M. acridum*) to 1.67 (*Botrytis cinerea*) with an average of 1.26 (**Table S22**).

4.2 Expansion and contraction of gene families

In order to identify gene families that had experienced expansion or contraction, an updated version of Café (version 3.1) (De Bie et al. 2006; Han et al. 2013) implemented with a probabilistic graphical model was used. This program regards the evolution of gene family as a stochastic birth and death process, where genes are gained and lost independently along each branch of a phylogenetic tree. The expansion and contraction events of gene families were computationally identified by

the comparisons of family (cluster) size differences between the most recent common ancestor (MRCA) and each of the current fungal species with the significant *P*-value of 0.05. Overall, we totally inferred 7,800 gene families presented in the MRCA of five entomopathogenic fungal species including *O. sinensis*, *C. militaris*, *B. bassiana*, *M. anisopliae* and *M. acridum* (**Fig. 2*B***). To facilitate the functional enrichment analysis, the PFAM terms for each gene within gene families were retrieved from InterProScan entry, following by Fisher's exact test (**Tables S23-24**).

4.3 Species-specific gene families

We classified the species-specific gene families according to the presence or absence of gene for a given species. In *O. sinensis*, a total of 49 families consisting of 1,077 genes were determined as species-specific. Among them, 318 (29.53%) could be functionally annotated. They were enriched in GO categories associated with starch binding (GO: 2001070; $P < 0.01$), pathogenesis (GO: 0009405; $P < 0.01$), and cell wall macromolecule catabolic process (GO: 0016998; $P < 0.01$) (**Table S25**), showing a species-specific adaptive evolution of infection mechanism in *O. sinensis*.

4.4 Identification of peroxidase gene family

To identify the peroxidase genes in five entomopathogenic fungi including *O. sinensis*, *T. inflatum*, *M. anisopliae, M. acridum*, *C. militaris*, *B. bassiana*, and *S. cerevisiae*, the protein sequence profiles for each peroxidase class were retrieved from the Fungal Peroxidase Database (fPoxDB; **Supplementary URLs**) (Choi et al. 2014). Based on these peroxidase profiles, we examined the peroxidase genes among species of interest using "*hmmsearch*" program implemented in Hmmer (version 3.1b1) (Eddy 2009; Finn et al. 2011; Finn et al. 2015) package with default parameters. This analysis resulted in a total of 42 (0.53%), 36 (0.36%), 28 (0.29%), 36 (0.35%), 42 (0.40%), 41 (0.42%), and 21 (0.39%) peroxidase genes in *O. sinensis*, *T. inflatum*, *C. militaris*, *B. bassiana*, *M. anisopliae*, *M. acridum*, and *S. cerevisiae*, respectively (**Fig. 3*A* and Table S26**). The classification of peroxidase genes was performed according to the corresponding descriptions in fPoxDB (Choi et al. 2014) (**Fig. 3*B***).

4.5 Identification of proteases, transporters and kinases

To detect the proteases in *O. sinensis* and other nine fungal species, we first downloaded all protein sequences of peptidase that are currently included in the MEROPS database (Release 10.0; http://merops.sanger.ac.uk/index.shtml) (Rawlings et al. 2016), which uses a hierarchical and structure-based method to classify the peptidases. The proteases in *O. sinensis* and other nine fungal species were then identified locally by using blastp searches against these MEROPS peptidase downloaded with a cutoff E-value of $1e^{-20}$. Finally, a total of 260 protease genes were identified in the *O. sinensis* genome (**Table S27**).

We identified and classified transporters in *O. sinensis* and other nine fungal genomes by using blastp searches against the Transport Classification Database (http://www.tcdb.org/) (Saier et al. 2006; Saier et al. 2009; Saier et al. 2014). Finally, a total of 494 membrane transport proteins were identified (**Table S34**).

To identify Kinases among *O. sinensis* and other nine fungal genomes, we performed Blastp searches against the KinBase database (http://kinase.com/) with a cutoff E value of $1e^{-10}$. As a result, a total of 133 kinases were identified in the *O. sinensis* genome (**Table S28-29**).

4.6 Identification of Carbohydrate-Active Enzymes (CAZymes)

Carbohydrate-Active enzymes (CAZymes) are responsible for the synthesis, degradation and modification of all the carbohydrates on Earth and thus are the most important class of enzymes particularly for bioenergy research (Yin et al. 2012). These enzymes are not only found in plants and bacteria, but also in fungi and animals, which are responsible for the synthesis, degradation and modification of all the glycoconjugates in nature including glycoproteins and glycolipids. We here detected CAZymes in *O. sinensis* and other nine fungal genomes by searching against dbCAN HMMs using HMMER3 (Eddy 2009; Finn et al. 2011; Finn et al. 2015). Briefly, the "*dbCAN-fam-HMMs.txt*" was first downloaded from dbCAN database (Yin et al. 2012) (http://csbl.bmb.uga.edu/dbCAN/index.php), and then HMM searches was performed

using “*hmmscan*”. The search results were finally parsed by the script of “*hmmscan-parser.sh*” that implemented in dbCAN database. The identified CAZymes for each fungal species were listed in **Table S30-32**.

4.7 Differential expression genes among three developmental stages

To investigate the expression profiles of genes during three developmental stages of *O. sinensis* with their length ratios of fungi *vs*. insect reaching ~ 1.20 ×, ~ 1.75 × and ~ 2.20 ×, we first mapped the RNA-Seq data from these three developmental stages of *O. sinensis* to the genome assembly using TopHat (version 2.1.0) (Trapnell et al. 2009) with default parameters. The expression level for each gene was then evaluated by Fragments Per Kilobase per Million mapped fragments (FPKM) and calculated using Cufflinks (version 2.1.1) (Trapnell et al. 2010) with default parameters. Gene expression levels were visualized and clustered using R package. We showed that a total of 411 genes were differentially expressed among these three developmental stages (**Fig. S14**). Functional annotation of these genes found that they were mainly involved in fungal pathogenicity, such as Glycosyl hydrolases family 16 (PF00722; FDR < 0.01), Cytochrome P450 (PF00067; FDR < 0.01) and Major Facilitator Superfamily (PF07690; FDR < 0.05). Besides, genes encoding enzymes associated with mitochondrial respiratory chain were also functionally enriched, such as NAD dependent epimerase / dehydratase family (PF01370; FDR < 0.01) and BCS1 N-terminal domain (PF08740; FDR < 0.01) (**Table S33**).

Supplementary section S5 – Phylogenetic analysis and positive selection test

5.1 Resolution of phylogenetic relationships among the thirteen fungi with fully sequenced genomes

To depict the phylogenetic relationships between *O. sinensis* and the other 12 closely related fungal species, we first identified a total of 1,499 one-to-one single-copy orthologous gene families using OrthoMCL. The coding sequences for each gene family were individually aligned using Muscle (version 3.8.31) (Edgar 2004a; Edgar 2004b), and eventually concatenated to a supergene for each species. The best substitution model of GTR+GAMMA was determined by ModelTest (version 2.1.7) (Guindon and Gascuel 2003; Darriba et al. 2012). Phylogenetic tree was finally constructed using RaxML package (version 8.1.13) (Stamatakis 2006; Stamatakis et al. 2008) with *S. cerevisiae* as outgroup. Bootstrap values were calculated from 1,000 iterations. The obtained trees were visualized using MEGA (version 5.05) (Kumar et al. 2001; Tamura et al. 2007; Tamura et al. 2011; Tamura et al. 2013) (**Fig. S13**).

5.2 Speciation timing

The divergence time between species was directly retrieved from TimeTree (Hedges et al. 2006; Kumar and Hedges 2011; Hedges et al. 2015), which looks for species in the TTOL to find their most recent common ancestor and produces the time estimate available.

5.3 Identification of positively selected genes

To detect genes that are evolving under positive selection in *O. sinensis* lineage, we computed the likelihood ratio test (LRT) *P*-values using the Codeml program with the optimized branch-site model implemented in the PAML package (Yang 2007). Genes with the *P*-value < 0.05 were retained and regarded as PSGs. As a result, a total of 163 PSGs were identified in *O. sinensis* (**Table S35**). Functional annotation of these

genes were carried out by InterProScan (version 5.3.46) (Zdobnov and Apweiler 2001; Quevillon et al. 2005; Jones et al. 2014) against PFAM database.

Supplementary section S6 – Analysis of mating type genes

6.1 Identification of mating-type (MAT) genes

We identified the mating-type genes of *O. sinensis* using BLAST (version 2.2.26) (Altschul et al. 1990) package. Firstly, protein sequences of mating-type genes from *Aspergillus nidulans* (Galagan et al. 2005) (AN2755 and AN4734), *Neurospora crassa* (Galagan et al. 2003) (NCU01958 and NCU01960), *C. militaris* (CCM_06523 and CCM_09679), *B. bassiana* (BBA_07733), *M. anisopliae* (MAA_03718, MAA_10977 and MAA_03719), and *M. acridum* (MAC_05350) were downloaded and searched against the *O. sinensis* genome using BLAST with an e-value of $1e^{-5}$. Hits with identity ≥ 30% and coverage ≥ 40% were retained and then manually checked. As a result, two types of mating-type genes, including *MAT1-1* (*OSIN7646*, *OSIN7647* and *OSIN7648*) and *MAT1-2* (*OSIN7649*), were identified in the *O. sinensis* genome (**Fig. S15*B***).

6.2 Verification using resequencing data

To verify the presence or absence of these two mating-type genes among different individuals, we collected 31 populations representing nearly all known range of *O. sinensis*, including Tibet Autonomous Region, Qinghai Province, Sichuan Province, Yunnan Province and Gansu Province (**Fig. S16 and Table S37**), and then sequenced them using Hiseq2000 platform (**Table S38**). After pre-procession, clean reads were individually mapped to the identified mating-type genes of *O. sinensis* using BWA (version 0.7.8) (Li and Durbin 2009). Reads mapping results were statistically converted into sequence coverage using samtools (version 0.1.19) (Li et al. 2009; Li 2011), and then displayed using R (version 3.0.1) (Ihaka and Gentleman 1996). Of the 31 examined individuals, we found 27 could be well confirmed to harbor these two types of mating-type genes with the exception of QH02, QH05, GS03 and SC08; the absence of *MAT1-1* or *MAT1-2* idiomorph may be caused by the heterogeneity and/or insufficient sequencing of those regions (**Table S36 and Fig. S15*A***).

6.3 Syntenic analysis of *MAT* loci between *O. sinensis* and *C. militaris*

We used MCScanX (Wang et al. 2012) to perform a syntenic analysis for *MAT* loci between *O. sinensis* and *C. militaris*. First, genomic positions and protein sequences of genes located within the flanking regions of *O. sinensis* and *C. militaris* were separately extracted from the gff3 annotation and fasta sequence files, respectively. Second, homology searches were conducted between the protein sequences using BLASTP with an e-value of $1e^{-5}$. Finally, based on gene positions, MCScanX was used to construct the syntenic map between *MAT* loci of *O. sinensis* and *C. militaris*. Results were visualized by SVG module (version 2.28; **Supplementary URLs**) with Perl (**Fig. S15*B***).

Supplementary section S7 – Genome resequencing and population genetic analysis

7.1 Sample collection and genome resequencing

A total of 31 wild individuals were harvested from the nearly known range of *O. sinensis* including Tibet Autonomous Region, Qinghai Province, Sichuan Province, Yunnan Province and Gansu Province (**Fig. S16 and Table S37**). Genomic DNA was extracted from fruiting bodies using CTAB method (Porebski et al. 1997). At least 5 μg of genomic DNA was used for each sample to construct sequencing libraries. According to the Illumina protocol, paired-end sequencing libraries with insert size of 300 bp were constructed and subsequently sequenced 100 bp at each end using Illumina Hiseq 2000 platform. Consequently, a total of ~36.68 Gb raw data was generated (**Table S38**).

7.2 Reads filtering, mapping and SNP calling

Raw sequencing reads were processed by following that used for the genome assembly (**Supplementary section 1.3**). This filtering resulted in a total of 31.25 Gb high-quality data for subsequent population genetic analyses. We aligned all clean reads from each sample to the *O. sinensis* genome assembly using BWA (version 0.7.8) (Li and Durbin 2009) package with default parameters. After alignment, the duplicated reads from PCR amplification were removed. Mapping result was then sorted by coordinates. We showed an average mapping rate of 82.17% for all sequenced individuals; the mapping rates varied among different populations, possibly resulting from genomic divergences between this assembled genome and the re-sequenced individuals (**Fig. S17**).

To perform SNP calling, the coordinate-sorted SAM mapping files were first converted into BAM format using samtools (version 0.1.19) (Li et al. 2009). And then SNP calling was conducted using a combination of methods that include a Bayesian approach as implemented in the package of samtools (version 0.1.19) and GATK

pipeline (version 3.1) (McKenna et al. 2010; DePristo et al. 2011; Van der Auwera et al. 2013) (**Fig. S18**). SNPs with depth (DP) < 4, RMS mapping quality (MQ) < 20, and proportion of missing data > 10% were defined as low quality and filtered out from final SNP data sets. Consequently, we totally identified 816,960 SNPs and 48,092 InDels (insertions and deletions) from 31 resequencing individuals of *O. sinensis* (**Fig. S18 and Table S39**).

7.3 Population genetic analyses

Only the bi-allelic SNPs without missing genotypes in all 31 individuals from the entire SNP dataset were used for subsequent analyses of population structure and levels of polymorphisms. File format conversions were conducted using VCFtools (version 0.1.12a) (Danecek et al. 2011). We used the SNPs to construct a neighbor-joining tree and performed a principal components analysis (PCA) to determine the population genetic structure of *O. sinensis*. The pairwise genetic distances between individuals were calculated and then used to construct the phylogenetic tree (NJ) using PhyML (version 3.0) (Guindon et al. 2010) with 1000 bootstrap replicates. The online server of EvolView (Zhang et al. 2012) (**Supplementary URLs**) was used for visualizing the phylogenetic tree. Principal component analysis (PCA) was performed by EIGENSOFT (version 6.0.1) (Patterson et al. 2006; Price et al. 2006), results of which were plotted using R (version 3.0.1). To investigate the population structure, we facilitated maximum likelihood clustering using frappe (version 1.1) (Tang et al. 2005) to infer the number of ancestral clusters (K) from 2 to 5 with 1000 iterations per run. The population structure of K = 3 was finally displayed by distruct (version 1.1) (Rosenberg 2004).

7.4 Assessment of nucleotide diversity and population differentiation

Nucleotide diversity (π) within a population was calculated using VCFtools (version 0.1.12a) (Danecek et al. 2011) with a fixed window size of 10 kb. The degree of differentiation among populations was measured by F_{ST} and computed using BioPerl (version 1.6.9; **Supplementary URLs**) with a 10 kb window along the genome.

Supplementary URLs

GapCloser: http://soap.genomics.org.cn/about.html;

RepeatMasker: http://repeatmasker.org/;

RepeatProteinMask: http://www.repeatmasker.org/RepeatProteinMask.html;

RepeatModeler: http://www.repeatmasker.org/RepeatModeler.html;

DOE Joint Genome Institute's (JGI) Genome Portal: http://genome.jgi.doe.gov/;

Program to Assemble Spliced Alignments (PASA): http://pasapipeline.github.io/;

EVidenceModeler (EVM): http://evidencemodeler.github.io/;

The Saccharomyces Genome Database (SGD): http://www.yeastgenome.org/;

Fungal Peroxidase Database: fPoxDB; http://peroxidase.riceblast.snu.ac.kr;

SVG module: http://search.cpan.org/~ronan/SVG-2.28/SVG/Manual.pm;

EvolView: http://www.evolgenius.info/evolview.html;

BioPerl: http://www.bioperl.org/wiki/Getting_BioPerl.

References

Altschul SF, Gish W, Miller W, Myers EW, Lipman DJ. 1990. Basic local alignment search tool. *Journal of Molecular Biology* **215**(3): 403-410.

Altschul SF, Madden TL, Schaffer AA, Zhang JH, Zhang Z, Miller W, Lipman DJ. 1997. Gapped BLAST and PSI-BLAST: a new generation of protein database search programs. *Nucleic Acids Research* **25**(17): 3389-3402.

Amselem J, Cuomo CA, van Kan JAL, Viaud M, Benito EP, Couloux A, Coutinho PM, de Vries RP, Dyer PS, Fillinger S et al. 2011. Genomic Analysis of the Necrotrophic Fungal Pathogens Sclerotinia sclerotiorum and Botrytis cinerea. *Plos Genetics* **7**(8).

Bao ZR, Eddy SR. 2002. Automated de novo identification of repeat sequence families in sequenced genomes. *Genome Research* **12**(8): 1269-1276.

Benson G. 1999. Tandem repeats finder: a program to analyze DNA sequences. *Nucleic Acids Research* **27**(2): 573-580.

Berbee ML, Taylor JW. 2010. Dating the molecular clock in fungi – how close are we? *Fungal Biology Reviews* **24**(1–2): 1-16.

Birney E, Clamp M, Durbin R. 2004. GeneWise and genomewise. *Genome Research* **14**(5): 988-995.

Birney E, Durbin R. 2000. Using GeneWise in the Drosophila annotation experiment. *Genome Research* **10**(4): 547-548.

Boetzer M, Henkel CV, Jansen HJ, Butler D, Pirovano W. 2011. Scaffolding pre-assembled contigs using SSPACE. *Bioinformatics* **27**(4): 578-579.

Bushley KE, Raja R, Jaiswal P, Cumbie JS, Nonogaki M, Boyd AE, Owensby CA, Knaus BJ, Elser J, Miller D et al. 2013. The Genome of Tolypocladium inflatum: Evolution, Organization, and Expression of the Cyclosporin Biosynthetic Gene Cluster. *Plos Genetics* **9**(6).

Camacho C, Coulouris G, Avagyan V, Ma N, Papadopoulos J, Bealer K, Madden TL. 2009. BLAST+: architecture and applications. *BMC Bioinformatics* **10**(1): 1-9.

Chen F, Mackey AJ, Stoeckert CJ, Jr., Roos DS. 2006. OrthoMCL-DB: querying a comprehensive multi-species collection of ortholog groups. *Nucleic Acids Research* **34**: D363-D368.

Choi J, Detry N, Kim K-T, Asiegbu FO, Valkonen JPT, Lee Y-H. 2014. fPoxDB: fungal peroxidase database for comparative genomics. *Bmc Microbiology* **14**.

Cuomo CA, Gueldener U, Xu J-R, Trail F, Turgeon BG, Di Pietro A, Walton JD, Ma L-J, Baker SE, Rep M et al. 2007. The Fusarium graminearum genome reveals a link between localized polymorphism and pathogen specialization. *Science* **317**(5843): 1400-1402.

Danecek P, Auton A, Abecasis G, Albers CA, Banks E, DePristo MA, Handsaker RE, Lunter G, Marth GT, Sherry ST et al. 2011. The variant call format and VCFtools. *Bioinformatics* **27**(15): 2156-2158.

Darriba D, Taboada GL, Doallo R, Posada D. 2012. jModelTest 2: more models, new heuristics and parallel computing. *Nature Methods* **9**(8): 772-772.

De Bie T, Cristianini N, Demuth JP, Hahn MW. 2006. CAFE: a computational tool for

the study of gene family evolution. *Bioinformatics* **22**(10): 1269-1271.

Dean RA, Talbot NJ, Ebbole DJ, Farman ML, Mitchell TK, Orbach MJ, Thon M, Kulkarni R, Xu J-R, Pan H et al. 2005. The genome sequence of the rice blast fungus Magnaporthe grisea. *Nature* **434**(7036): 980-986.

DePristo MA, Banks E, Poplin R, Garimella KV, Maguire JR, Hartl C, Philippakis AA, del Angel G, Rivas MA, Hanna M et al. 2011. A framework for variation discovery and genotyping using next-generation DNA sequencing data. *Nature Genetics* **43**(5): 491-+.

DiGuistini S, Wang Y, Liao NY, Taylor G, Tanguay P, Feau N, Henrissat B, Chan SK, Hesse-Orce U, Alamouti SM et al. 2011. Genome and transcriptome analyses of the mountain pine beetle-fungal symbiont Grosmannia clavigera, a lodgepole pine pathogen. *Proceedings of the National Academy of Sciences of the United States of America* **108**(6): 2504-2509.

Eddy SR. 2009. A new generation of homology search tools based on probabilistic inference. *Genome informatics International Conference on Genome Informatics* **23**(1): 205-211.

Edgar RC. 2004a. MUSCLE: a multiple sequence alignment method with reduced time and space complexity. *Bmc Bioinformatics* **5**: 1-19.

-. 2004b. MUSCLE: multiple sequence alignment with high accuracy and high throughput. *Nucleic Acids Research* **32**(5): 1792-1797.

Enright AJ, Van Dongen S, Ouzounis CA. 2002. An efficient algorithm for large-scale detection of protein families. *Nucleic Acids Research* **30**(7): 1575-1584.

Finn RD, Clements J, Arndt W, Miller BL, Wheeler TJ, Schreiber F, Bateman A, Eddy SR. 2015. HMMER web server: 2015 update. *Nucleic Acids Research* **43**(W1): W30-W38.

Finn RD, Clements J, Eddy SR. 2011. HMMER web server: interactive sequence similarity searching. *Nucleic Acids Research* **39**: W29-W37.

Galagan JE, Calvo SE, Borkovich KA, Selker EU, Read ND, Jaffe D, FitzHugh W, Ma L-J, Smirnov S, Purcell S et al. 2003. The genome sequence of the filamentous fungus Neurospora crassa. *Nature* **422**(6934): 859-868.

Galagan JE, Calvo SE, Cuomo C, Ma LJ, Wortman JR, Batzoglou S, Lee SI, Basturkmen M, Spevak CC, Clutterbuck J et al. 2005. Sequencing of Aspergillus nidulans and comparative analysis with A-fumigatus and A-oryzae. *Nature* **438**(7071): 1105-1115.

Gao Q, Jin K, Ying S-H, Zhang Y, Xiao G, Shang Y, Duan Z, Hu X, Xie X-Q, Zhou G et al. 2011. Genome Sequencing and Comparative Transcriptomics of the Model Entomopathogenic Fungi Metarhizium anisopliae and M. acridum. *Plos Genetics* **7**(1).

Goffeau A, Barrell B, Bussey H, Davis R, Dujon B, Feldmann H, Galibert F, Hoheisel J, Jacq C, Johnston M. 1996. Life with 6000 genes. *Science* **274**(5287): 546-567.

Grabherr MG, Haas BJ, Yassour M, Levin JZ, Thompson DA, Amit I, Adiconis X, Fan L, Raychowdhury R, Zeng Q et al. 2011. Full-length transcriptome assembly from RNA-Seq data without a reference genome. *Nature*

Biotechnology **29**(7): 644-U130.
Grigoriev IV, Nikitin R, Haridas S, Kuo A, Ohm R, Otillar R, Riley R, Salamov A, Zhao X, Korzeniewski F et al. 2014. MycoCosm portal: gearing up for 1000 fungal genomes. *Nucleic Acids Research* **42**(D1): D699-D704.
Grigoriev IV, Nordberg H, Shabalov I, Aerts A, Cantor M, Goodstein D, Kuo A, Minovitsky S, Nikitin R, Ohm RA et al. 2012. The Genome Portal of the Department of Energy Joint Genome Institute. *Nucleic Acids Research* **40**(D1): D26-D32.
Guindon S, Dufayard J-F, Lefort V, Anisimova M, Hordijk W, Gascuel O. 2010. New Algorithms and Methods to Estimate Maximum-Likelihood Phylogenies: Assessing the Performance of PhyML 3.0. *Systematic Biology* **59**(3): 307-321.
Guindon S, Gascuel O. 2003. A simple, fast, and accurate algorithm to estimate large phylogenies by maximum likelihood. *Systematic Biology* **52**(5): 696-704.
Haas BJ, Delcher AL, Mount SM, Wortman JR, Smith RK, Hannick LI, Maiti R, Ronning CM, Rusch DB, Town CD et al. 2003. Improving the Arabidopsis genome annotation using maximal transcript alignment assemblies. *Nucleic Acids Research* **31**(19): 5654-5666.
Haas BJ, Salzberg SL, Zhu W, Pertea M, Allen JE, Orvis J, White O, Buell CR, Wortman JR. 2008. Automated eukaryotic gene structure annotation using EVidenceModeler and the program to assemble spliced alignments. *Genome Biology* **9**(1).
Han MV, Thomas GWC, Lugo-Martinez J, Hahn MW. 2013. Estimating Gene Gain and Loss Rates in the Presence of Error in Genome Assembly and Annotation Using CAFE 3. *Molecular Biology and Evolution* **30**(8): 1987-1997.
Hedges SB, Dudley J, Kumar S. 2006. TimeTree: a public knowledge-base of divergence times among organisms. *Bioinformatics* **22**(23): 2971-2972.
Hedges SB, Marin J, Suleski M, Paymer M, Kumar S. 2015. Tree of Life Reveals Clock-Like Speciation and Diversification. *Molecular Biology and Evolution* **32**(4): 835-845.
Ihaka R, Gentleman R. 1996. R: a language for data analysis and graphics. *Journal of computational and graphical statistics* **5**(3): 299-314.
Jones P, Binns D, Chang H-Y, Fraser M, Li W, McAnulla C, McWilliam H, Maslen J, Mitchell A, Nuka G et al. 2014. InterProScan 5: genome-scale protein function classification. *Bioinformatics* **30**(9): 1236-1240.
Jurka J. 2000. Repbase Update - a database and an electronic journal of repetitive elements. *Trends in Genetics* **16**(9): 418-420.
Jurka J, Kapitonov VV, Pavlicek A, Klonowski P, Kohany O, Walichiewicz J. 2005. Repbase update, a database of eukaryotic repetitive elements. *Cytogenetic and Genome Research* **110**(1-4): 462-467.
Kasuga T, White TJ, Taylor JW. 2002. Estimation of Nucleotide Substitution Rates in Eurotiomycete Fungi. *Molecular Biology and Evolution* **19**(12): 2318-2324.
Klosterman SJ, Subbarao KV, Kang S, Veronese P, Gold SE, Thomma BPHJ, Chen Z, Henrissat B, Lee Y-H, Park J et al. 2011. Comparative Genomics Yields Insights into Niche Adaptation of Plant Vascular Wilt Pathogens. *Plos*

Pathogens **7**(7).
Korf I. 2004. Gene finding in novel genomes. *BMC Bioinformatics* **5**(1): 1-9.
Kumar S, Hedges SB. 2011. TimeTree2: species divergence times on the iPhone. *Bioinformatics* **27**(14): 2023-2024.
Kumar S, Tamura K, Jakobsen IB, Nei M. 2001. MEGA2: molecular evolutionary genetics analysis software. *Bioinformatics* **17**(12): 1244-1245.
Li H. 2011. A statistical framework for SNP calling, mutation discovery, association mapping and population genetical parameter estimation from sequencing data. *Bioinformatics* **27**(21): 2987-2993.
Li H, Durbin R. 2009. Fast and accurate short read alignment with Burrows–Wheeler transform. *Bioinformatics* **25**(14): 1754-1760.
Li H, Handsaker B, Wysoker A, Fennell T, Ruan J, Homer N, Marth G, Abecasis G, Durbin R, Genome Project Data P. 2009. The Sequence Alignment/Map format and SAMtools. *Bioinformatics* **25**(16): 2078-2079.
Li L, Stoeckert CJ, Roos DS. 2003. OrthoMCL: Identification of ortholog groups for eukaryotic genomes. *Genome Research* **13**(9): 2178-2189.
Li M, Tian S, Jin L, Zhou G, Li Y, Zhang Y, Wang T, Yeung CKL, Chen L, Ma J et al. 2013. Genomic analyses identify distinct patterns of selection in domesticated pigs and Tibetan wild boars. *Nature Genetics* **45**(12): 1431-U1180.
Marcais G, Kingsford C. 2011. A fast, lock-free approach for efficient parallel counting of occurrences of k-mers. *Bioinformatics* **27**(6): 764-770.
Margulies M, Egholm M, Altman WE, Attiya S, Bader JS, Bemben LA, Berka J, Braverman MS, Chen YJ, Chen ZT et al. 2005. Genome sequencing in microfabricated high-density picolitre reactors. *Nature* **437**(7057): 376-380.
McKenna A, Hanna M, Banks E, Sivachenko A, Cibulskis K, Kernytsky A, Garimella K, Altshuler D, Gabriel S, Daly M et al. 2010. The Genome Analysis Toolkit: A MapReduce framework for analyzing next-generation DNA sequencing data. *Genome Research* **20**(9): 1297-1303.
Nordberg H, Cantor M, Dusheyko S, Hua S, Poliakov A, Shabalov I, Smirnova T, Grigoriev IV, Dubchak I. 2014. The genome portal of the Department of Energy Joint Genome Institute: 2014 updates. *Nucleic Acids Research* **42**(D1): D26-D31.
Patterson N, Price AL, Reich D. 2006. Population structure and eigenanalysis. *Plos Genetics* **2**(12): 2074-2093.
Porebski S, Bailey LG, Baum BR. 1997. Modification of a CTAB DNA extraction protocol for plants containing high polysaccharide and polyphenol components. *Plant Molecular Biology Reporter* **15**(1): 8-15.
Price AL, Jones NC, Pevzner PA. 2005. De novo identification of repeat families in large genomes. *Bioinformatics* **21**(1): 351-358.
Price AL, Patterson NJ, Plenge RM, Weinblatt ME, Shadick NA, Reich D. 2006. Principal components analysis corrects for stratification in genome-wide association studies. *Nature genetics* **38**(8): 904-909.
Quevillon E, Silventoinen V, Pillai S, Harte N, Mulder N, Apweiler R, Lopez R. 2005. InterProScan: protein domains identifier. *Nucleic Acids Research* **33**:

W116-W120.
Rawlings ND, Barrett AJ, Finn R. 2016. Twenty years of the MEROPS database of proteolytic enzymes, their substrates and inhibitors. *Nucleic Acids Research* **44**(D1): D343-D350.
Rosenberg NA. 2004. DISTRUCT: a program for the graphical display of population structure. *Molecular Ecology Notes* **4**(1): 137-138.
Rouxel T, Grandaubert J, Hane JK, Hoede C, van de Wouw AP, Couloux A, Dominguez V, Anthouard V, Bally P, Bourras S et al. 2011. Effector diversification within compartments of the Leptosphaeria maculans genome affected by Repeat-Induced Point mutations. *Nature Communications* **2**.
Saier MH, Reddy VS, Tamang DG, Vastermark A. 2014. The Transporter Classification Database. *Nucleic Acids Research* **42**(D1): D251-D258.
Saier MH, Tran CV, Barabote RD. 2006. TCDB: the Transporter Classification Database for membrane transport protein analyses and information. *Nucleic Acids Research* **34**: D181-D186.
Saier MH, Yen MR, Noto K, Tamang DG, Elkan C. 2009. The Transporter Classification Database: recent advances. *Nucleic Acids Research* **37**: D274-D278.
She R, Chu JSC, Wang K, Pei J, Chen N. 2009. genBlastA: Enabling BLAST to identify homologous gene sequences. *Genome Research* **19**(1): 143-149.
Simao FA, Waterhouse RM, Ioannidis P, Kriventseva EV, Zdobnov EM. 2015. BUSCO: assessing genome assembly and annotation completeness with single-copy orthologs. *Bioinformatics* **31**(19): 3210-3212.
Slater GS, Birney E. 2005. Automated generation of heuristics for biological sequence comparison. *BMC Bioinformatics* **6**.
Stamatakis A. 2006. RAxML-VI-HPC: Maximum likelihood-based phylogenetic analyses with thousands of taxa and mixed models. *Bioinformatics* **22**(21): 2688-2690.
Stamatakis A, Hoover P, Rougemont J. 2008. A Rapid Bootstrap Algorithm for the RAxML Web Servers. *Systematic Biology* **57**(5): 758-771.
Stanke M, Keller O, Gunduz I, Hayes A, Waack S, Morgenstern B. 2006. AUGUSTUS: ab initio prediction of alternative transcripts. *Nucleic Acids Research* **34**: W435-W439.
Stanke M, Morgenstern B. 2005. AUGUSTUS: a web server for gene prediction in eukaryotes that allows user-defined constraints. *Nucleic Acids Research* **33**: W465-W467.
Stanke M, Steinkamp R, Waack S, Morgenstern B. 2004. AUGUSTUS: a web server for gene finding in eukaryotes. *Nucleic Acids Research* **32**: W309-W312.
Tamura K, Dudley J, Nei M, Kumar S. 2007. MEGA4: Molecular evolutionary genetics analysis (MEGA) software version 4.0. *Molecular Biology and Evolution* **24**(8): 1596-1599.
Tamura K, Peterson D, Peterson N, Stecher G, Nei M, Kumar S. 2011. MEGA5: Molecular Evolutionary Genetics Analysis Using Maximum Likelihood, Evolutionary Distance, and Maximum Parsimony Methods. *Molecular*

Biology and Evolution **28**(10): 2731-2739.

Tamura K, Stecher G, Peterson D, Filipski A, Kumar S. 2013. MEGA6: Molecular Evolutionary Genetics Analysis Version 6.0. *Molecular Biology and Evolution* **30**(12): 2725-2729.

Tang H, Peng J, Wang P, Risch NJ. 2005. Estimation of individual admixture: Analytical and study design considerations. *Genetic Epidemiology* **28**(4): 289-301.

Van der Auwera GA, Carneiro MO, Hartl C, Poplin R, del Angel G, Levy-Moonshine A, Jordan T, Shakir K, Roazen D, Thibault J et al. 2013. From FastQ Data to High-Confidence Variant Calls: The Genome Analysis Toolkit Best Practices Pipeline. In *Current Protocols in Bioinformatics*, Vol 43, pp. 11.10.11-11.10.33. John Wiley & Sons, Inc.

van Dongen S, Abreu-Goodger C. 2012. Using MCL to extract clusters from networks. *Methods in molecular biology (Clifton, NJ)* **804**: 281-295.

Wang Y, Tang H, DeBarry JD, Tan X, Li J, Wang X, Lee T-h, Jin H, Marler B, Guo H et al. 2012. MCScanX: a toolkit for detection and evolutionary analysis of gene synteny and collinearity. *Nucleic Acids Research* **40**(7).

Xiao G, Ying S-H, Zheng P, Wang Z-L, Zhang S, Xie X-Q, Shang Y, St Leger RJ, Zhao G-P, Wang C et al. 2012. Genomic perspectives on the evolution of fungal entomopathogenicity in Beauveria bassiana. *Scientific Reports* **2**.

Yin YB, Mao XZ, Yang JC, Chen X, Mao FL, Xu Y. 2012. dbCAN: a web resource for automated carbohydrate-active enzyme annotation. *Nucleic Acids Research* **40**(W1): W445-W451.

Zdobnov EM, Apweiler R. 2001. InterProScan - an integration platform for the signature-recognition methods in InterPro. *Bioinformatics* **17**(9): 847-848.

Zhang H, Gao S, Lercher MJ, Hu S, Chen W-H. 2012. EvolView, an online tool for visualizing, annotating and managing phylogenetic trees. *Nucleic Acids Research* **40**(W1): W569-W572.

Zhang Q-J, Zhu T, Xia E-H, Shi C, Liu Y-L, Zhang Y, Liu Y, Jiang W-K, Zhao Y-J, Mao S-Y et al. 2014. Rapid diversification of five Oryza AA genomes associated with rice adaptation. *Proceedings of the National Academy of Sciences of the United States of America* **111**(46): E4954-E4962.

Zheng P, Xia Y, Xiao G, Xiong C, Hu X, Zhang S, Zheng H, Huang Y, Zhou Y, Wang S et al. 2011. Genome sequence of the insect pathogenic fungus Cordyceps militaris, a valued traditional chinese medicine. *Genome Biology* **12**(11): R116.

Zheng W, Huang L, Huang J, Wang X, Chen X, Zhao J, Guo J, Zhuang H, Qiu C, Liu J et al. 2013. High genome heterozygosity and endemic genetic recombination in the wheat stripe rust fungus. *Nature Communications* **4**: 2673.

Supplementary Figures

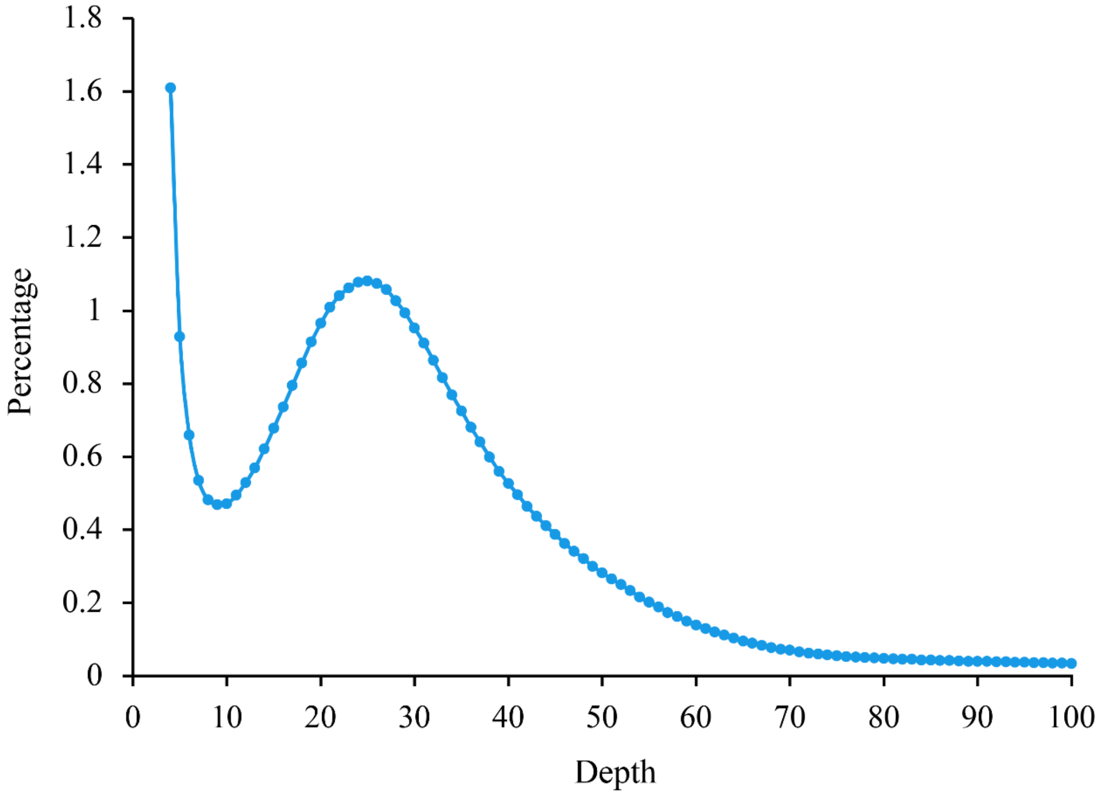

Fig. S1. K-mer analysis for estimating the genome size of *Ophiocordyceps sinensis*. The figure shows 17-mer depth distribution of the *O. sinensis* sequencing reads. The left, truncated peak at low frequency and high volume represents *K-mer*s containing essentially random sequencing errors, whereas the right distribution shows proper (putatively error-free) data. A total of 2,994,486,107 17-mers are obtained, and the volume peak depth is 25. The genome size was estimated based on the formula: Genome size = (Total K-mer number) / (Position of peak depth), which is 2,994,486,107 / 25 = 119.8 Mb.

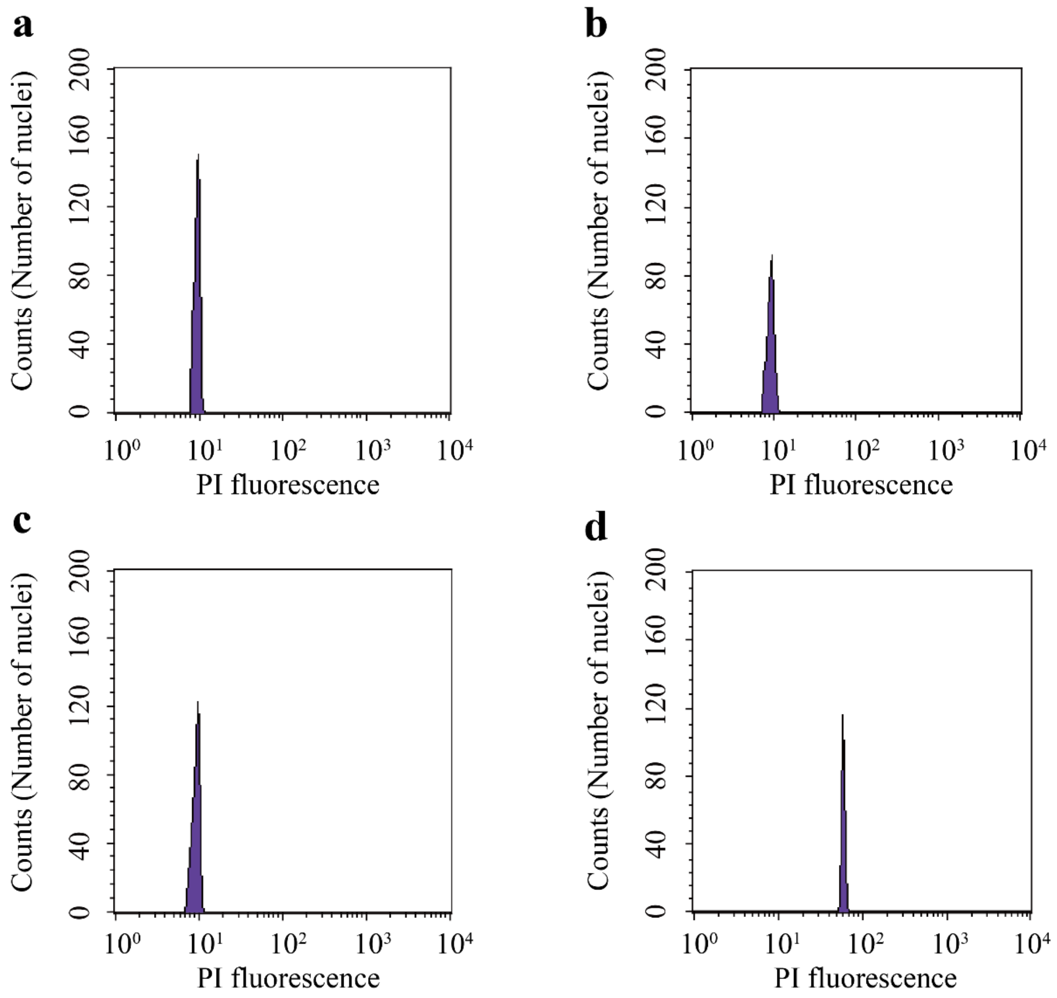

Fig. S2. Genome size estimation using flow cytometry with an improved Otto buffer. To accurately estimate the genome size of *O. sinensis*, three replications were separately performed. Coefficient of variation values (CVs) for these three experiments were: **a**) 4.39%; **b**) 5.02%; and **c**) 4.80%. **d**) The rice (*Oryza sativa* ssp. *japonica* cv. Nipponbare) was selected as inner standard, and the CV for which was 2.75. The genome size of *O. sinensis* was finally estimated to be 124.08 Mb.

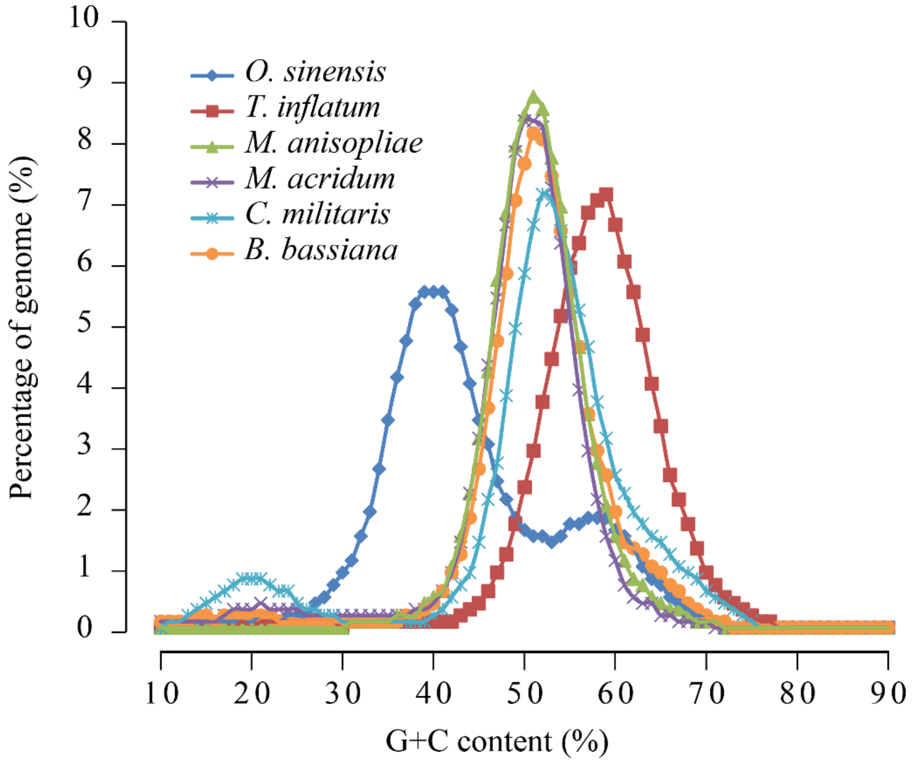

Fig. S3. Distribution of GC content across *O. sinensis* and other five entomopathogenic fungi. GC contents for each species were calculated based on the 500 bp, non-overlapping sliding windows across the *O. sinensis* and five other entomopathogenic fungal genomes.

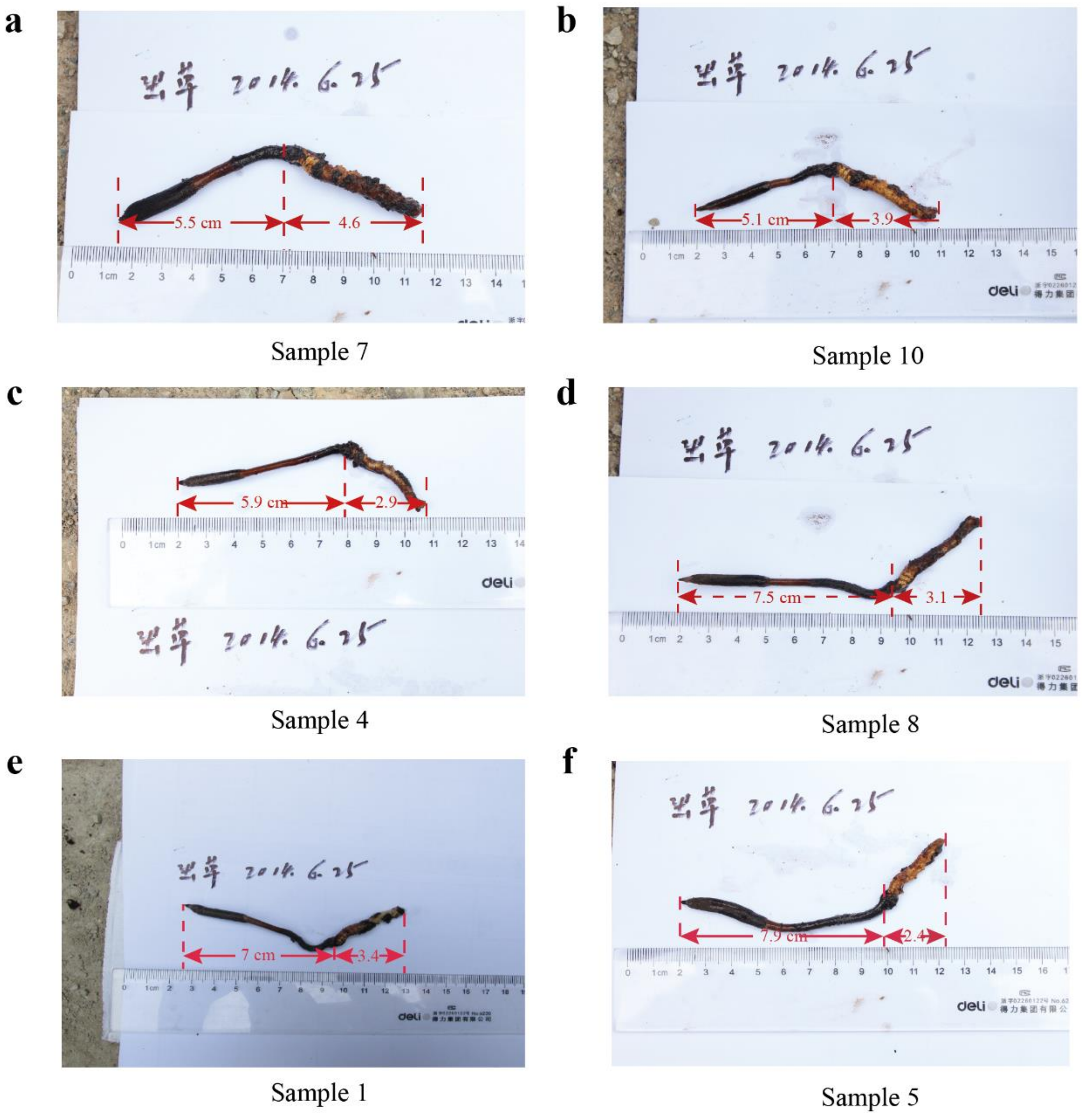

Fig. S4. Sample collection for RNA sequencing (RNA-Seq). Six individuals with the length ratios of fungi / insect reaching ~ 1.20 x (**a-b**), ~ 1.75 x (**c-d**) and ~ 2.20 x (**e-f**) were collected from the Deqin County of Yunnan Province, China to represent different developmental stages.

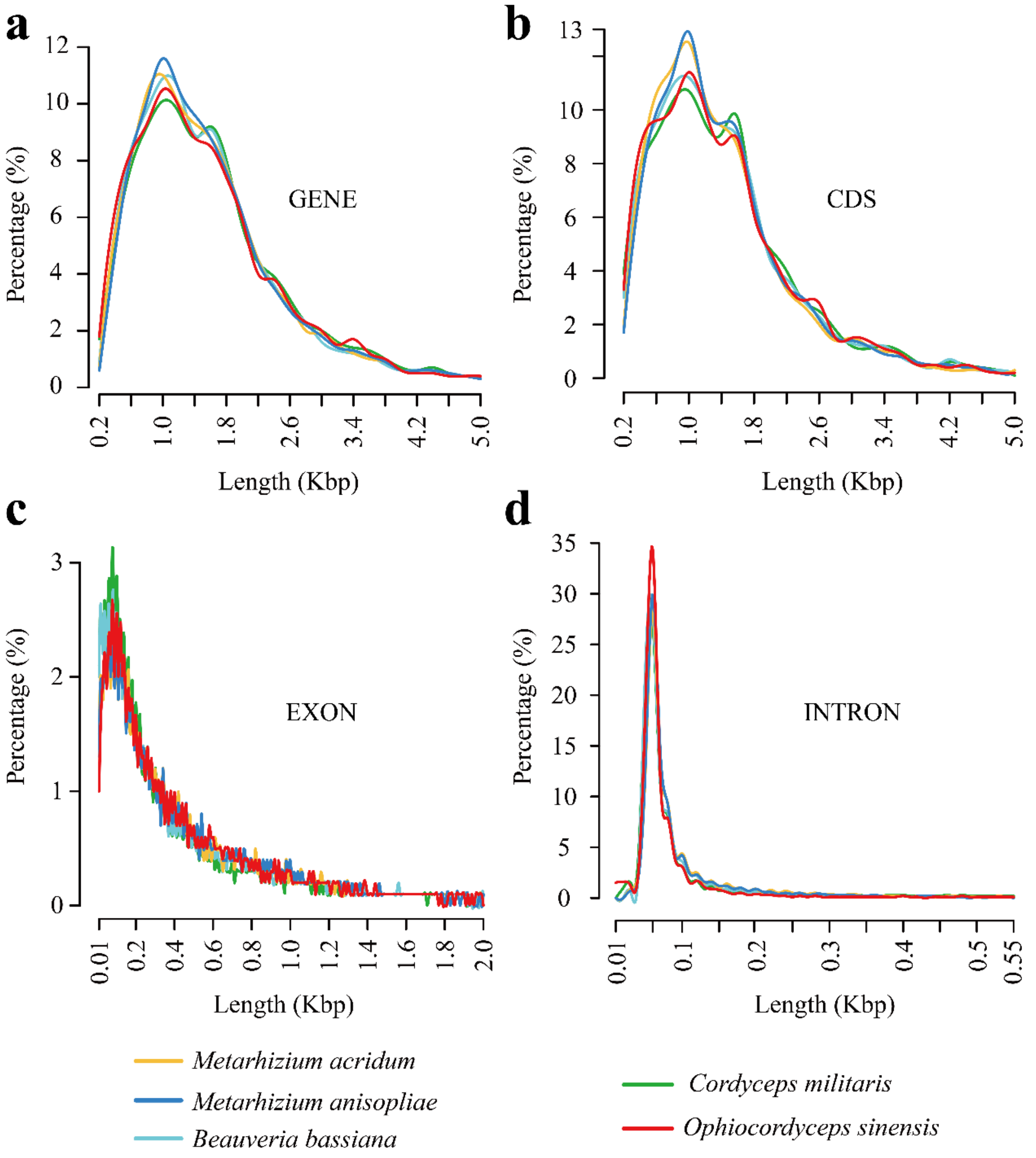

Fig. S5. Comparison of gene parameters among the sequenced insect-pathogenic fungal genomes. No obvious unexpected length differences of (**a**) gene, (**b**) CDS, (**c**) exon and (**d**) intron were observed for *O. sinensis*, indicating high quality of gene structure annotation.

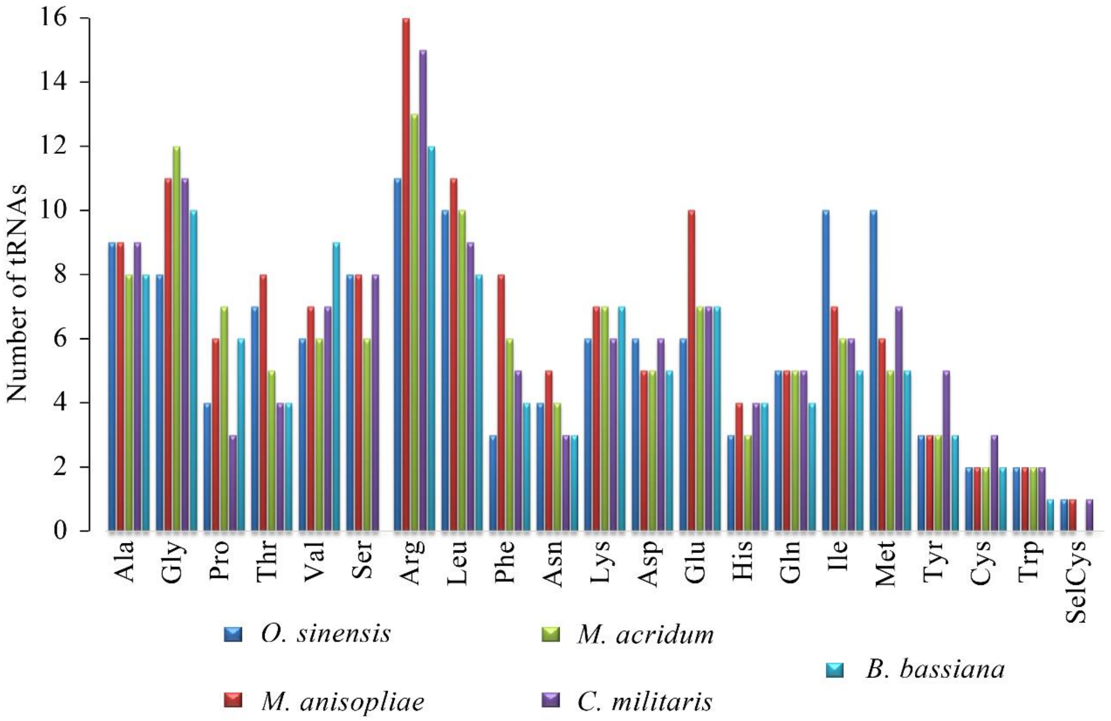

Fig. S6. Distribution of tRNAs among *O.sinensis* and other four entomopathogenic fungi. The x-axis represents 21 amino acids (AAs), including 20 standard AAs and one selenocysteine (SelCys), while the y-axis indicates the number of tRNAs decoding each type of AAs.

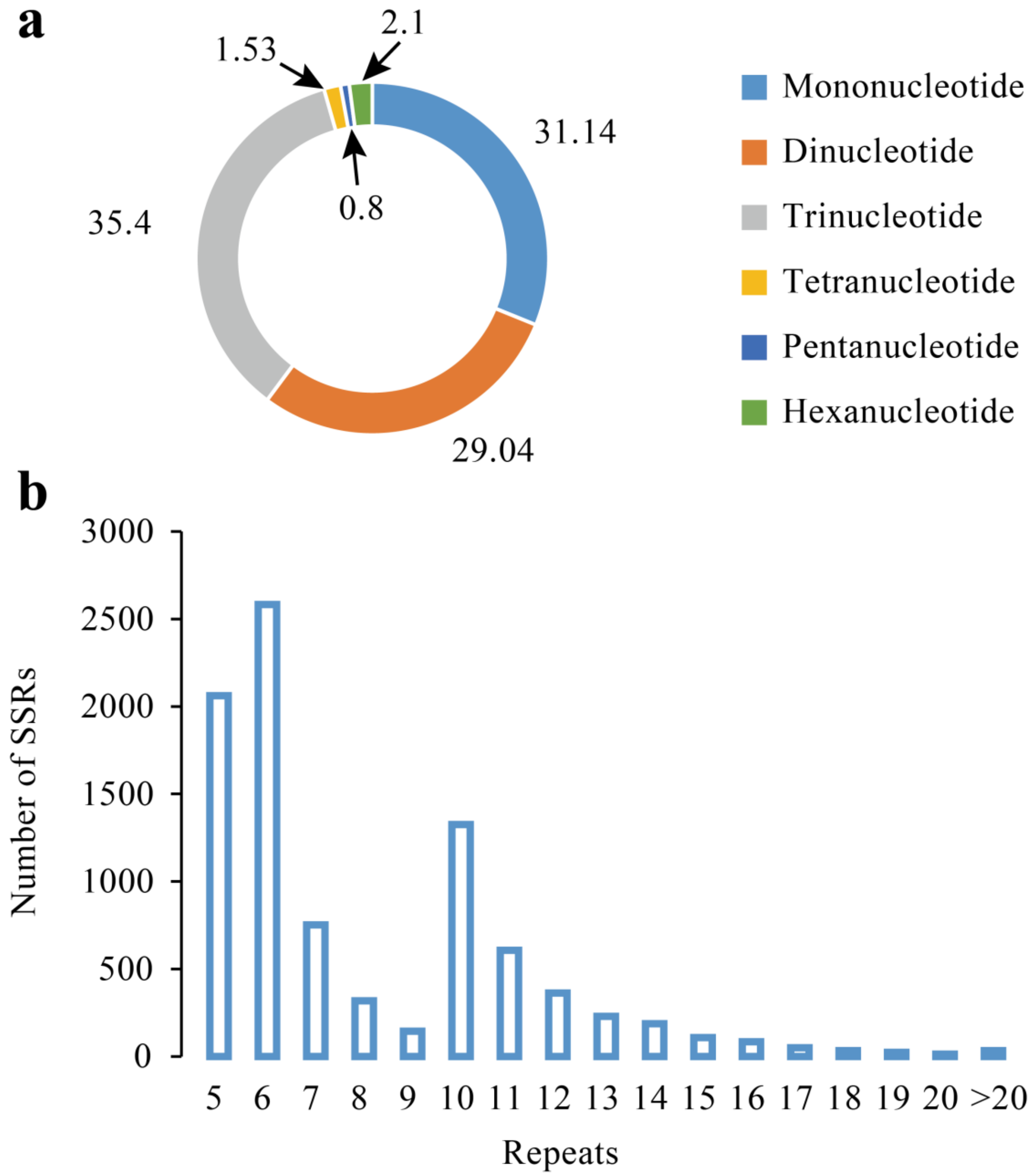

Fig. S7. Overview of SSRs detected in *O. sinensis* genome. **a**) Proportion of different types of SSRs; **b**) Repeat distribution of SSRs. Six types of SSRs from mono-nucleotides to hexa-nucleotides were identified using the MISA Perl script (http://pgrc.ipk-gatersleben.de/misa/). The minimum repeat unit size for mono-nucleotides was set at ten and six for di-, respectively, and at five for tri- to hexa-nucleotides.

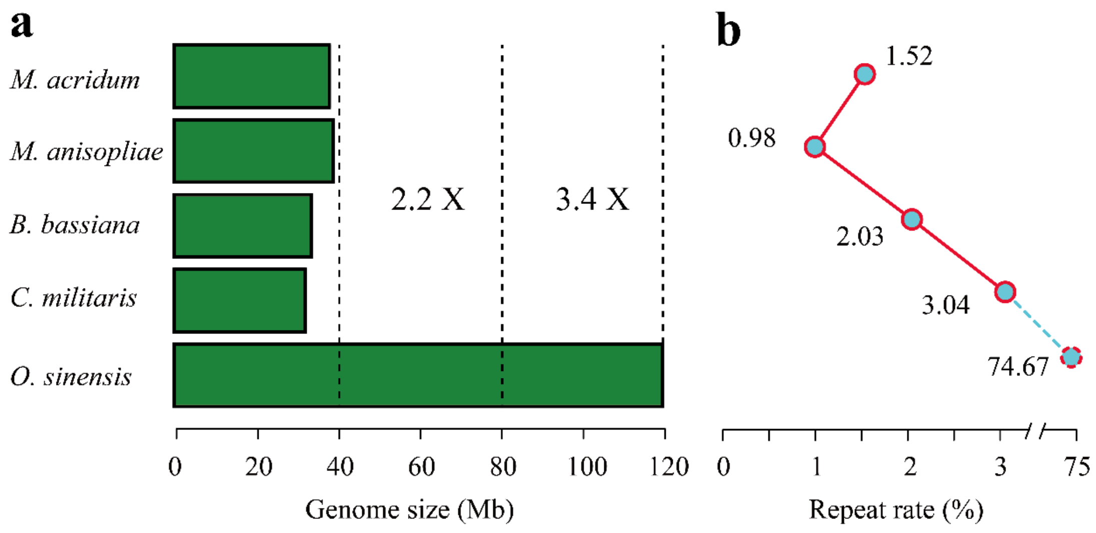

Fig. S8. Genome size variation and repeat content among *O. sinensis* and other five sequenced insect-pathogenic fungal genomes. **a**) Genome size variation; **b**) Content of repeat sequences. The estimated genome size of *O. sinensis* was 120 Mb, which is 3.4-fold larger than the average size of its closely species (37.73 Mb) including *M. acridum*, *M. anisopliae*, *B. bassiana* and *C. militaris*.

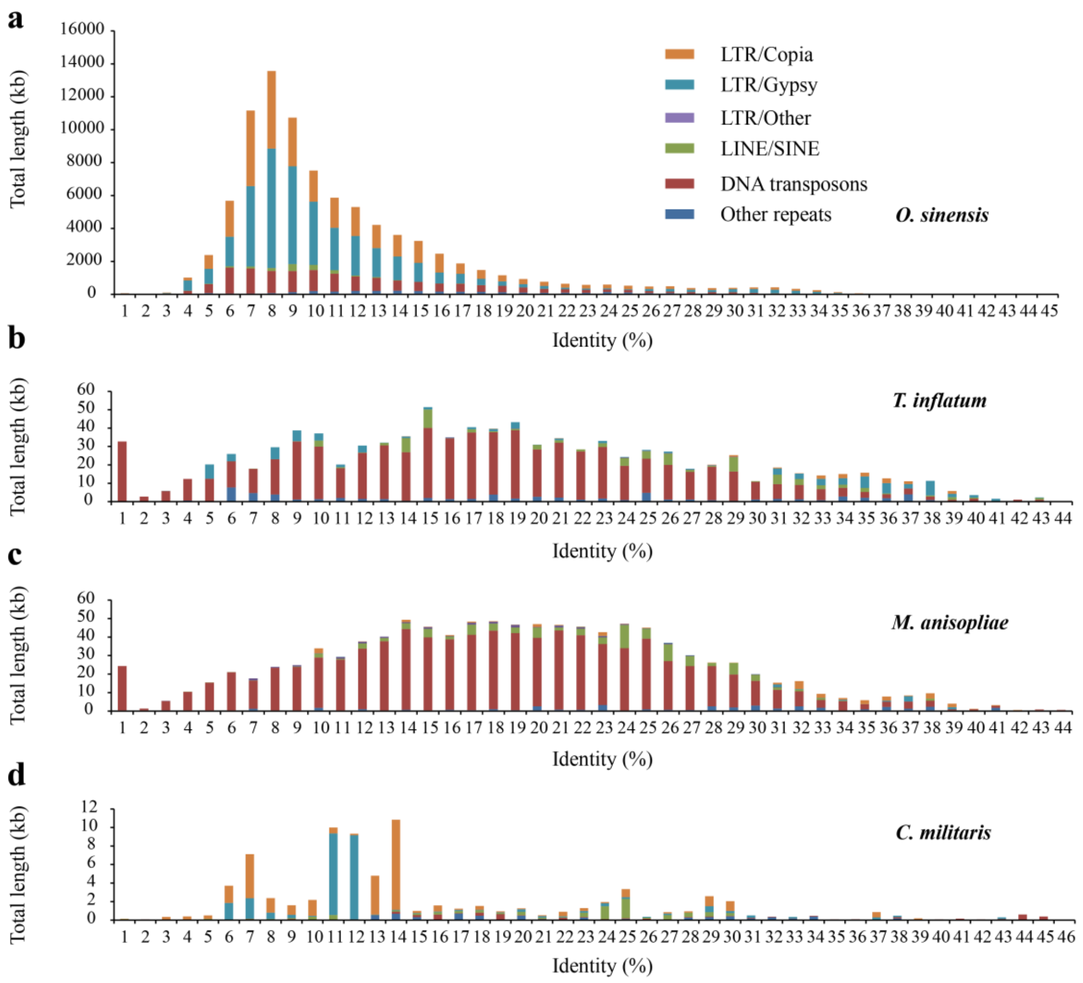

Fig. S9. Distribution of divergence rate for different types of repeat sequences among *O. sinensis* and other three fungal species. a) *O. sinensis*; **b**) *T. inflatum*; **c**) *M. anisopliae*; **d**) *C. militaris*. The divergence rate was calculated between the identified repeat sequences in the genome and consensus sequence in the library (Repbase or RepeatModeler). For the conversion of nucleotide sequence distance to putative genome insertion age, a substitution rate of 1.05×10^{-9} mutations per site per year was used. The insertion time was counted by T = K/2r. T: element insertion time; r: synonymous mutation/site/year; K: the divergence between the LTRs and consensus sequence in the TE library.

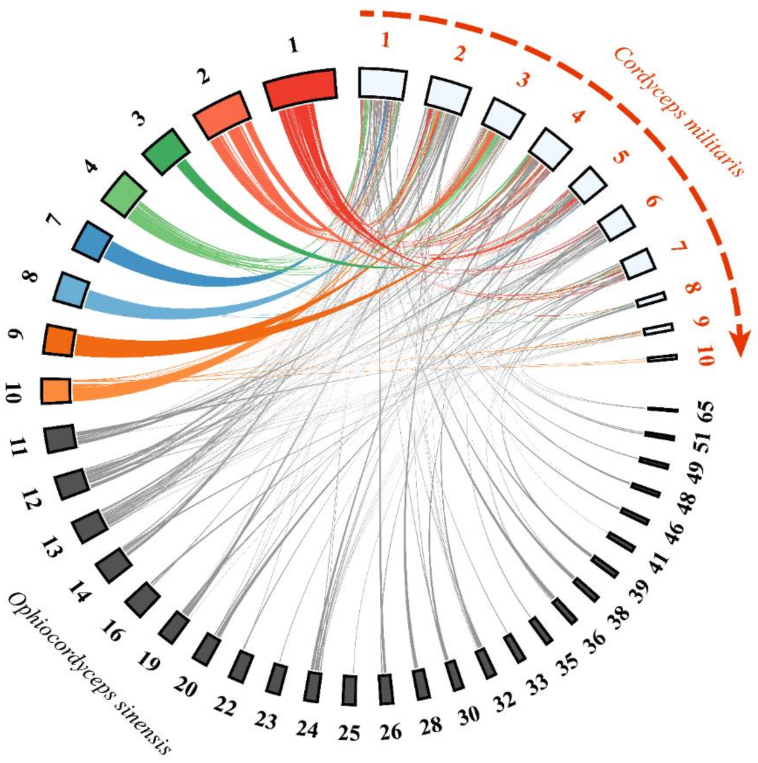

Fig. S10. Macro-synteny between *O. sinensis* and *C. militaris* genomes. Only the largest ten scaffolds of *C. militaris* colored in light grey were compared with corresponding scaffolds *O. sinensis*. Syntenic genomic regions larger than 10 Kb are connected and shown in corresponding colors. Comparisons between these two genome sequences were performed with Blast program and MCScanX package.

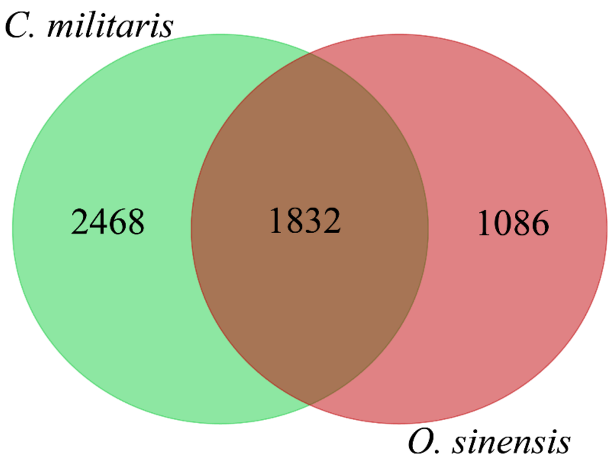

Fig. S11. Venn diagram shows the shared and lineage-specific gene families for non-collinear genes of *O. sinensis* and *C. militaris*. The shared 1,832 genes include those 1,041 from *O. sinensis* and 791 from *C. militaris*, respectively.

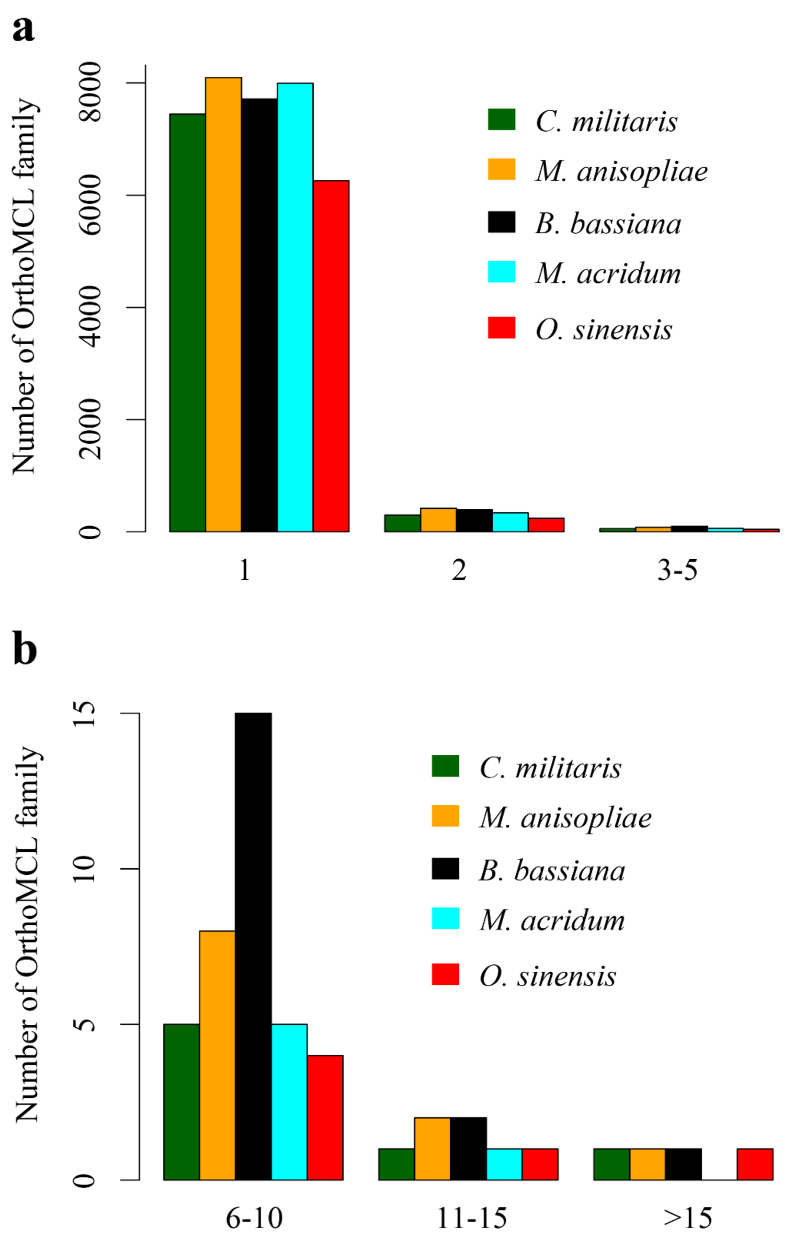

Fig. S12. Family size distribution among *O. sinensis* and other four entomopathogenic fungi. a) Family size ≤ 5; **b**) Family size ≥ 6. The gene families (clusters) were identified using OrthoMCL package with the default parameters. The x-axis represents the family size, while y-axis indicates the number of OrthoMCL gene clusters for each family size.

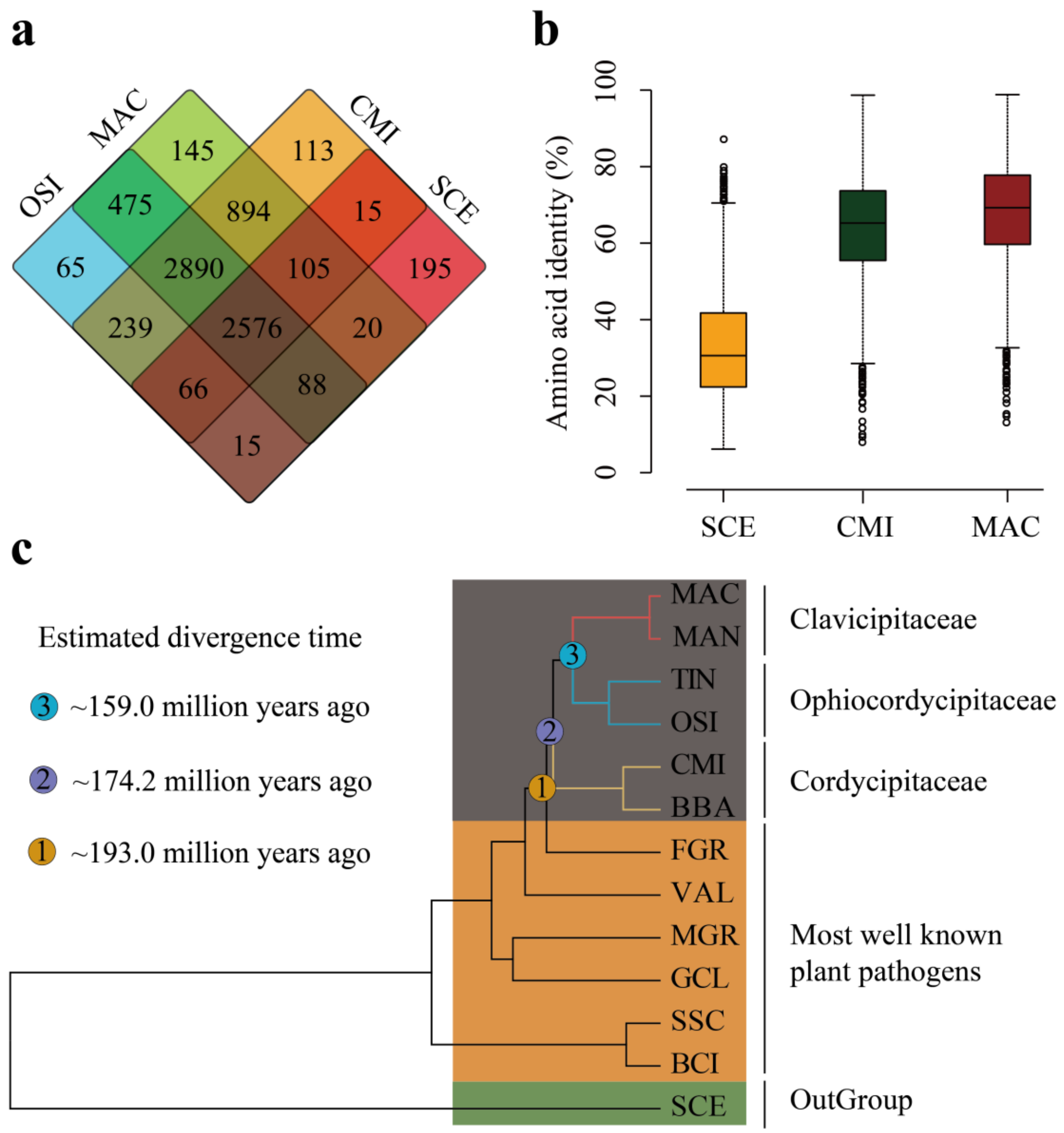

Fig. S13. Comparative genomic analyses of 13 fungal species and their phylogeny. **a**) Venn diagram shows the gene families among and within the three insect-pathogenic fungal genomes and yeast. **b**) The amino acid similarity of 1:1-orthologues between *O. sinensis* and *C. militaris*, *O. sinensis* and *M. acridum*, *O. sinensis* and *S. cerevisiae*, respectively. **c**) Genome-based phylogenetic tree of 13 Ascomycota species constructed by using 1,499 single-copy orthologoues. Grey box indicates entomopathogenic fungi, orange box depicts most of the well-known plant pathogens, and green box represents the used out-group.

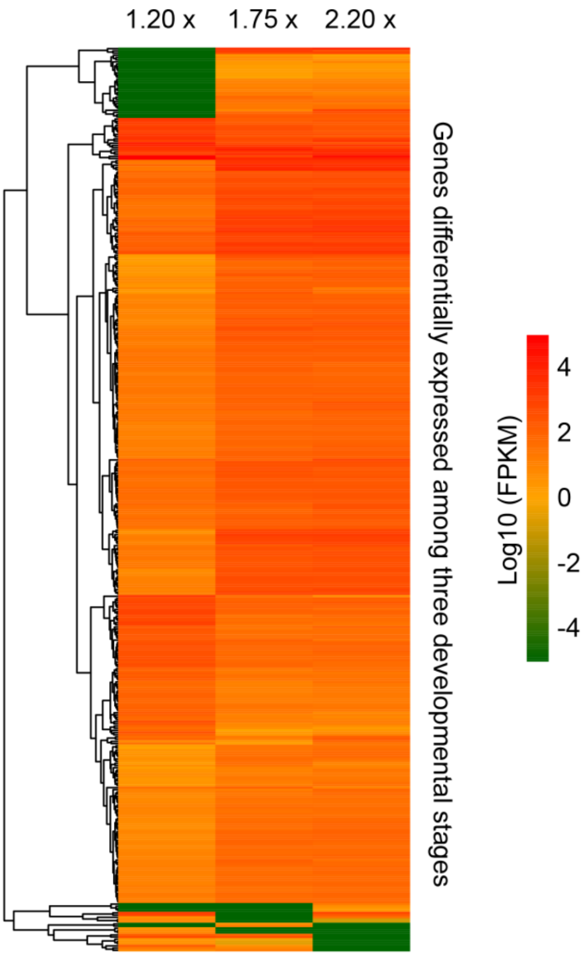

Fig. S14. The 411 genes differentially expressed among the three developmental stages of *O. sinensis*. Six samples with length ratios of fungus *vs*. insect reaching ~1.20 ×, ~1.75 × and ~2.20 × were collected from the Deqin County of Yunnan Province, China, to represent different developmental stages. The gene expression levels were calculated using Cufflinks (version 2.2.1) based on the mapping results from Tophat (version 2.1.0).

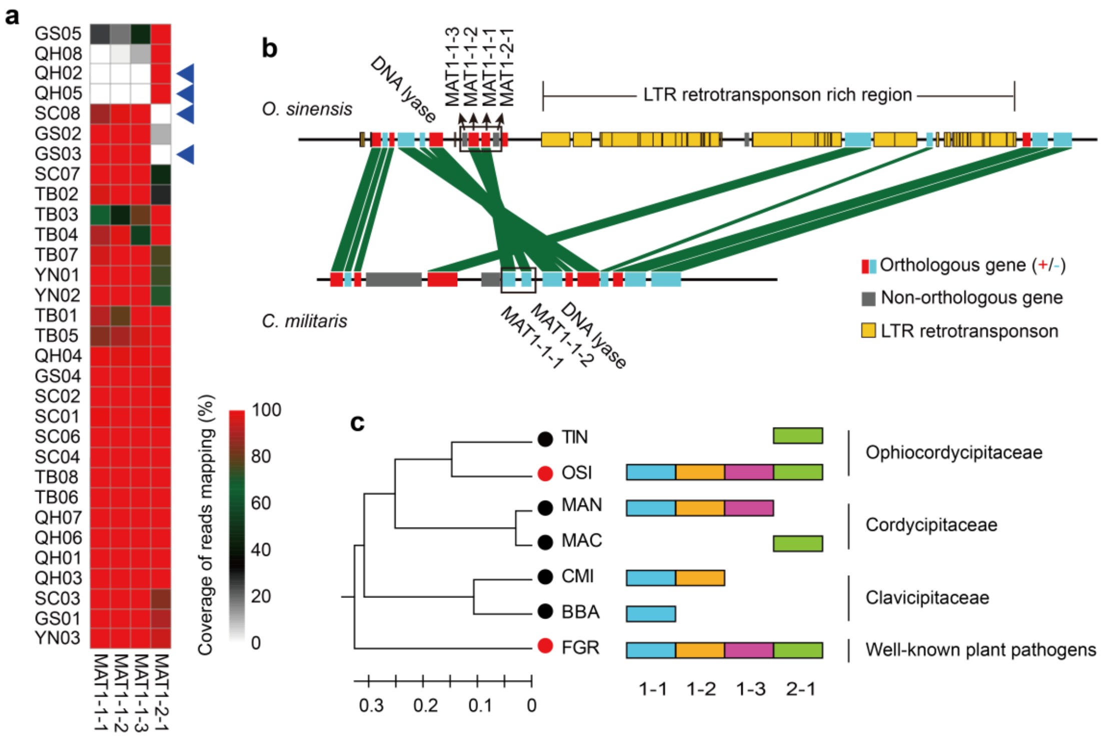

Fig. S15. Comparative analysis of mating-type (MAT) locus in *O. sinensis*. a) Reads were mapped to the *O. sinensis* idiomorphic region with the coverage represented as a heat map. Mapped reads verify the presence of both *MAT1-1* (i.e., *MAT1-1-1*, *MAT1-1-2*, and *MAT1-1-3*) and *MAT1-2* (*MAT1-2-1*) mating-type locus in 31 individuals of *O. sinensis* with the exception of QH02, QH05, SC08 and GS03 accessions. The absence of *MAT1-1* or *MAT1-2* idiomorph may indicate that the regions are lost or high sequence heterogeneity of the locus. **b)** Synteny of the MAT loci and their flanking regions between *O. sinensis* and *C. militaris* (as determined by SVG bioperl). Mating-type genes are framed and the orthologous genes are connected with green joining lines band. **c)** Evolution of mating system in *O. sinensis*. The heterothallic and homothallic fungi species are separately colored in black and red solid circles.

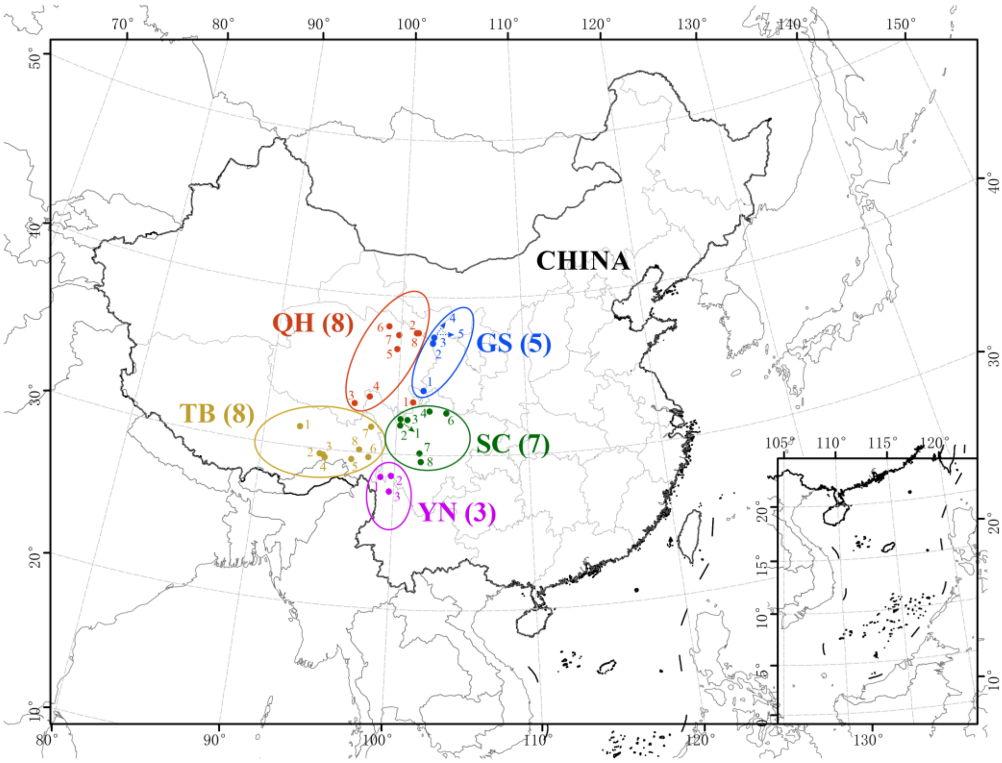

Fig. S16. Geographical distribution of the 31 *O. sinensis* populations for genome re-sequencing. These samples were collected during harvesting seasons (May to June) between 2007 and 2010. They spanned the known distribution range of *O. sinensis*, including Tibet Autonomous Region (TB), Qinghai (QH), Sichuan (SC), Yunnan (YN), and Gansu (GS) provinces. The map was generated using ArcGIS software (version 10.1; https://www.arcgis.com/features/index.html).

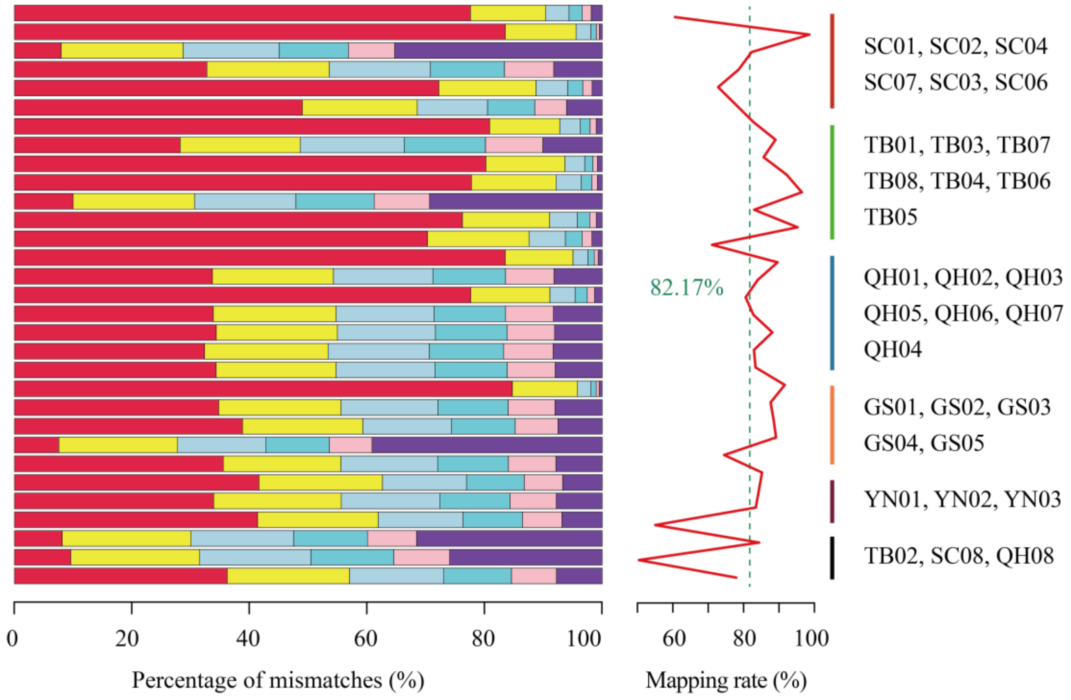

Fig. S17. Statistic of mapping rate for 31 resequencing genomes against the *O. sinensis* genome assembly. We performed the reads mapping using BWA (version 0.7.8) with default parameters. The average mapping rate of 82.17% was colored in green.

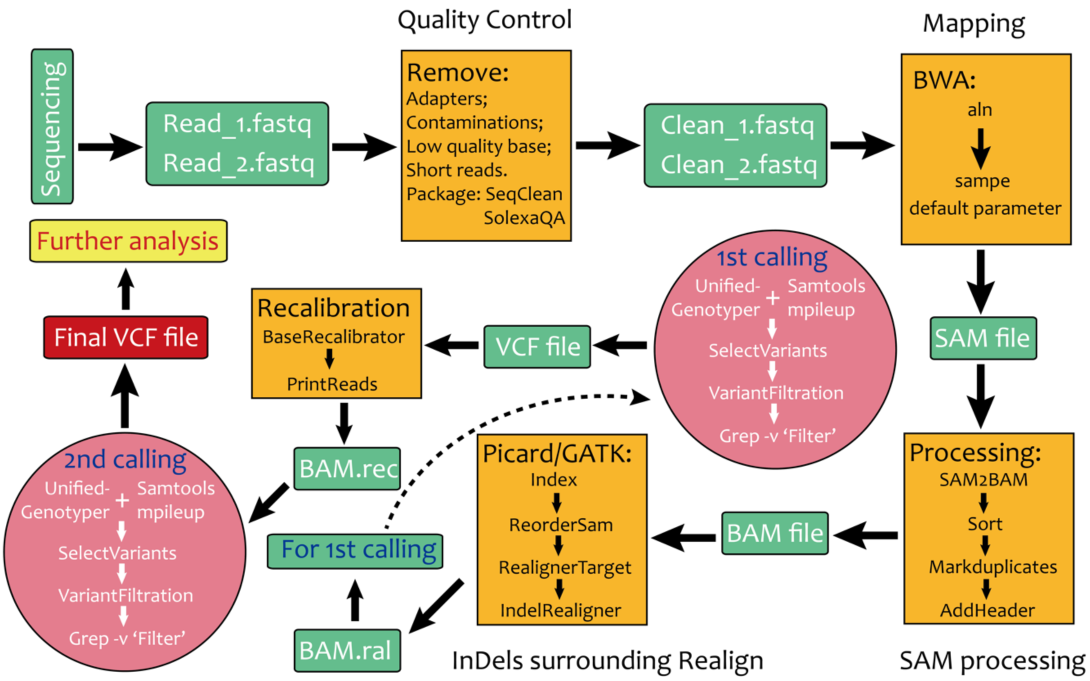

Fig. S18. Pipeline used for variation calling. The SNP, insertion and deletion were called using an integrated approach of GATK and SAMtools pipeline.

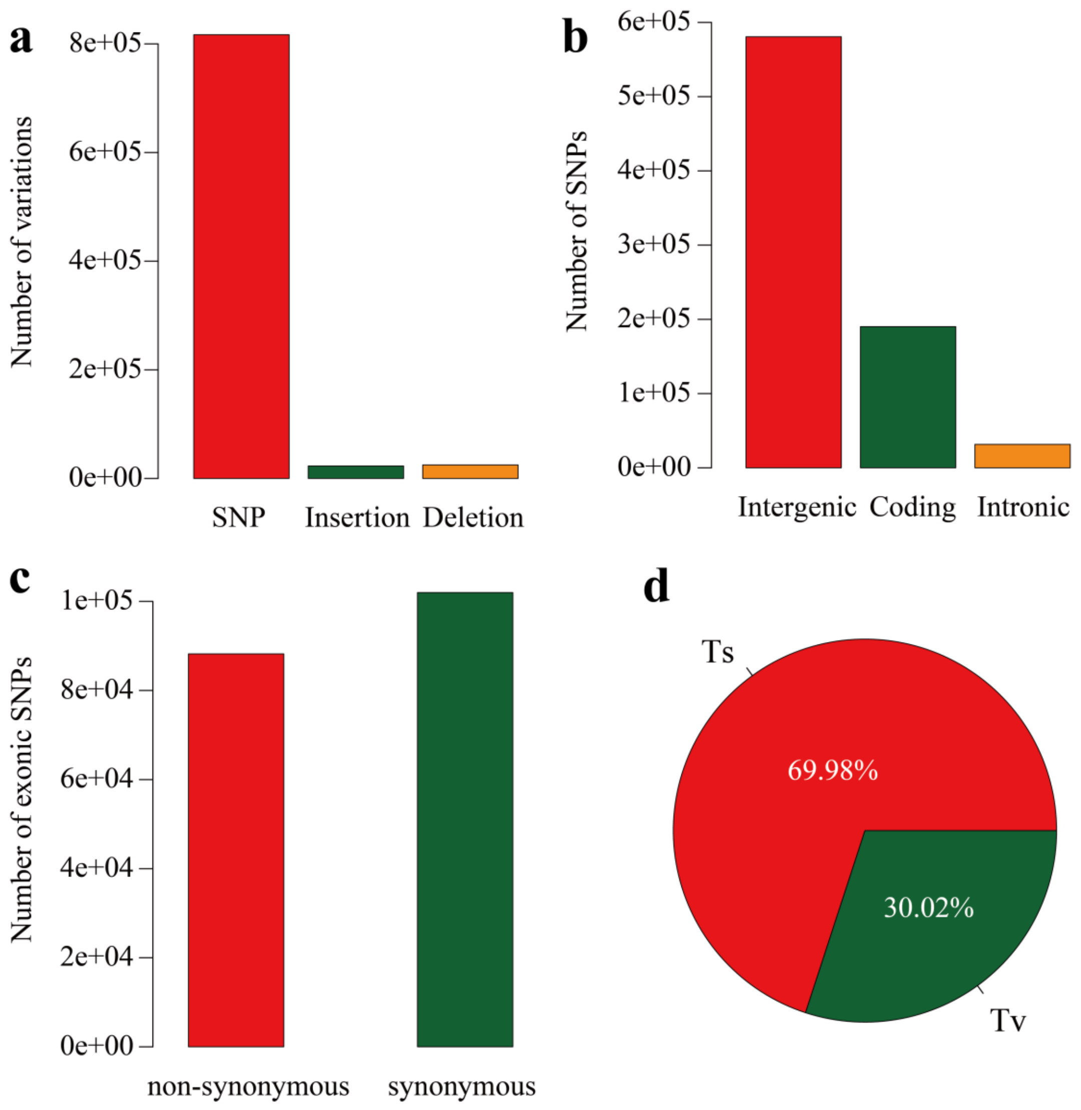

Fig. S19. Overview of the variation calling using samtools and GATK pipeline. a) Basic information of variation calling; **b**) Genomic distribution of SNPs; **c**) Effects of SNPs regarding the altering of amino acids; **d**) Ratio of transition (Ts) against transversion (Tv).

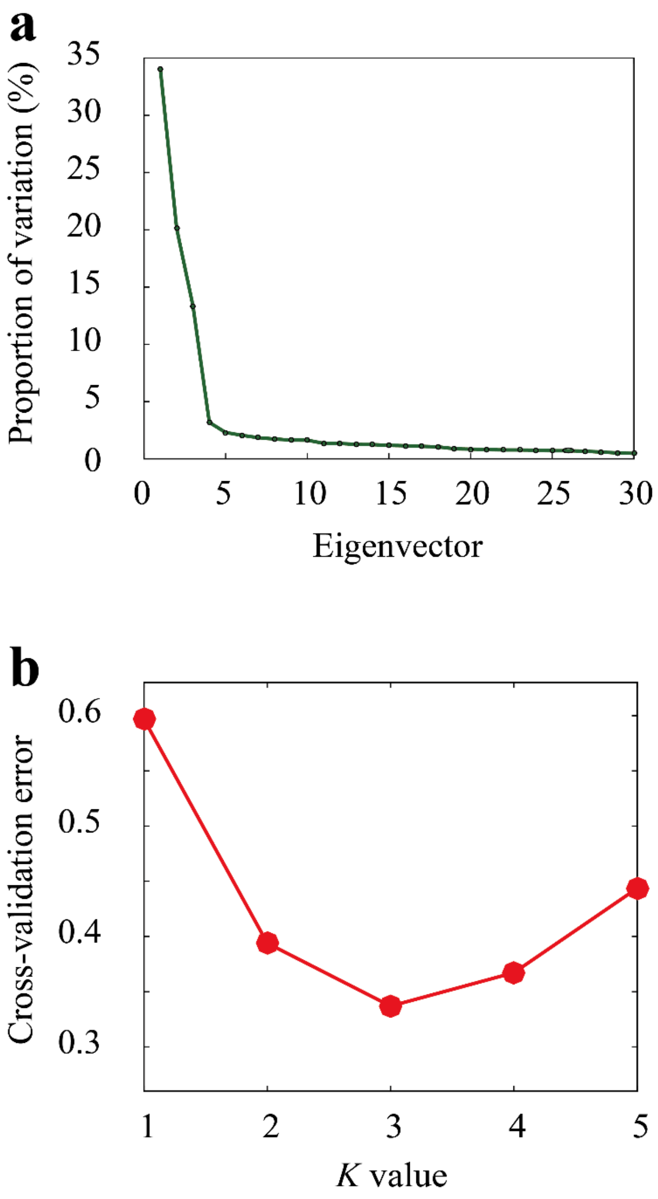

Fig. S20. Population structure analysis for 31 accessions of *O. sinensis*. a) Proportion of variations explained by eigenvectors used for PCA analyses. The leading 5 eigenvectors represent 71.4% variance as 34.5% for eigenvector 1 and 20.1% for eigenvector 2; **b**) Cross-validation error for different population classified. *K* = 3 shows the lowest cross-validation error.

Supplementary Tables

Table S1. Summary of sequencing data of *Ophiocordyceps sinensis* genome.

**Library**	**Platform**	**Insert size (bp)**	**Read length (bp)**	**Raw data (Mb)**	**Coverage (×)***	**Clean data (Mb)**	**Coverage (×)***
F9-1-1	454 FLX	N/A	679	~421	3.5	~372	3.1
F9-1-2	454 FLX	N/A	701	~427	3.6	~381	3.2
F9-1-3	454 FLX	N/A	689	~506	4.2	~451	3.8
F9-1-4	454 FLX	N/A	703	~488	4.1	~437	3.6
F9-3k	Hiseq2000	3,000	101	~3,471	28.9	~1,858	15.5
F9-8k	Hiseq2000	8,000	101	~3,647	30.4	~1,906	15.9
**Total**				~8,961	74.7	~5,406	45.1

* The estimated genome size: ~120 Mb.

Table S2. Estimation of *O. sinensis* genome size using flow cytometry and *K*-mer analysis.

**Flow cytometry**				
Round	Mean	CV (%) [a]	Genome size (Mb)	Average (Mb)
1st	18.323	4.39	126.96	
2nd	18.200	5.02	126.10	124.08
3rd	17.200	4.80	119.18	
Inner Standard[b]	57.730	2.75	400.00	400.00
***K*-mer analysis**				
Reads	*K*-mer	*K*-mer number	*K*-mer depth	Genome size (Mb)
454	17	2,994,486,107	25	119.80

[a] Coefficient of variation values;

[b] *Oryza sativa* ssp. *japonica* cv. Nipponbare was selected as inner standard.

Table S3. Statistic of the *O. sinensis* genome assembly.

**Sequence type**	***O. sinensis***	***O. sinensis*** [a]
**Contig**		
Number (#)	9,141	26,584
Total length (bp)	112,055,914	73,983,396
N50 (bp) [b]	21,423	5,253
Largest (bp)	120,072	87,316
**Scaffold**		
Number (#)	1,141	10,603
Number (> 2Kb)	156	8,346
Total length (bp)	116,423,023	78,515,811
N50 (bp) [b]	2,999,605	11,986
N90 (bp) [b]	679,076	3,431
Largest (bp)	9,561,753	130,882
Gap length (bp)	4,370,108	4,532,415
GC content (%)	43.09	46.19
Completeness (%) [c]	97.02	65.43

[a] Previously assembled by Hu *et al*. Chinese Science Bulletin 2013, 58: 2846-2854;

[b] The N50 and N90 values of the genome assembly were calculated using the fragment with its length ≥ 1 kb;

[c] The estimated genome size of 120 Mb was used.

Table S4. Statistic of DNA base composition of the *O. sinensis* genome.

**Base**	**Number (bp)**	**% of genome**
A	30,975,250	26.61
T	30,907,632	26.55
C	25,071,671	21.53
G	25,098,328	21.56
N	4,370,138	3.75
GC*	50,169,999	43.09
**Total**	116,423,023	100%

* GC content of the genome without N.

Table S5. Validation of genome assembly using reads mapping, sequence alignments and BUSCO method. All the DNA and EST sequences of *O. sinensis* were retrieved from the NCBI public database (http://www.ncbi.nlm.nih.gov/) using the key words of “txid72228 [Organism: exp]”.

	**Total**	**Mapped**	**Rate (%)**
**Reads mapping**			
454	2,648,101	2,621,902	99.01
**Sequences available in public databases**			
DNA	10,603	1,0481*/9,014**/6,703***	98.85*/85.01**/63.95***
EST	16,676	15,897*/15,298**/15,068***	95.33*/91.74**/90.36***
**Transcripts assembled in this study**			
Unigenes	11,742	11,064*/10,719**/10,449***	94.23*/91.29**/88.99***
**BUSCOs from Ascomycota lineage********			
Complete	1,315	1,236	94.0
Duplicated	1,315	2	0.2
Fragmented	1,315	52	4.0
Missing	1,315	25	1.8

* Hits with Coverage >= 50% and Identity >= 90%;

** Hits with Coverage >= 80% and Identity >= 90%;

*** Hits with Coverage >= 90% and Identity >= 90%;

**** The 1,315 BUSCO conserved genes used were collected from Ascomycota lineage.

Table S6. Sample collection and RNA sequencing (RNA-Seq) of *O. sinensis* transcriptome. A total of six individuals representing three developmental stages (average length ratio for fungus/insect ranged from ~ 1.2× to 2.2×) of *O. sinensis* were harvested from Deqin County of Yunnan Province, China. The raw sequencing data were trimmed, and only those high-quality ones (shadowed; phred quality score ≥ 20 and length ≥ 25 bp) were retained for subsequent analyses.

**Collection**						
Sample name	S7	S10	S4	S8	S1	S5
Insect (cm)	5	4.3	3.5	4	3.5	3.4
Fungus (cm)	6	5.2	6	7.5	7.4	8.1
Total (cm)	11	9.5	9.5	11.5	10.9	11.5
Fold (x)*	1.20	1.21	1.71	1.88	2.11	2.38
**Classification**						
Type	1.20	1.20	1.75	1.75	2.20	2.20
**Sequencing**						
Read number (million)	24.78	26.27	24.98	25.32	23.42	24.29
Total length (Gb)	2.50	2.65	2.52	2.56	2.36	2.45
Read length (bp)	101	101	101	101	101	101
GC content (%)	57.98	58.03	58.17	58.05	58.13	58.00
Total length (Gb)	2.45	2.59	2.47	2.50	2.31	2.40
Read length (bp)	99	99	99	99	99	99
GC content (%)	57.92	57.96	58.11	57.98	58.08	57.93

Note: Rows colored in gray represent clean data.

Table S7. Summary of the *O. sinensis* transcriptome assembly.

Transcriptome Features	Value
Total number of unigenes (#)	11,742
Total length (bp)	21,687,501
N10 (bp)	8,411
N20 (bp)	6,530
N30 (bp)	5,353
N40 (bp)	4,485
N50 (bp)	3,793
Median length (bp)	910
Average length (bp)	1,847
Percent GC	59.52

Note: RNA-Seq data were assembled using Trinity (version v2.0.6) with default parameters except for "--*jaccard_clip*", which was normally used for dealing with fungal species that harbors high gene density.

Table S8. Statistic of the predicted protein-coding genes in *O. sinensis* genome.

	O. sinensis	***O. sinensis*** [a]	***C. militaris***
Total number of genes (#)	7,939	6,972	9,684
Gene length in genome (%)	12.56	14.06	52.12
Average gene length (bp)	1,693	1,351	1,743
Gene density (gene per Mb)	68	87	299
Average CDS length (bp)	1,504	1,193	1,517
Average CDS GC ratio (%)	61.49	60.32	58.58
Average exon length (bp)	532	483	507
Average exons GC ratio (%)	61.49	60.31	58.58
Average exon per gene	2.8	2.6	3
Average intron length (bp)	103	107	113
Intron GC content (%)	56.76	53.73	48.25
Putative PHI genes (#) [b]	2,166	998	1,547

[a] Previously assembled by Hu *et al*. (2013), Chinese Science Bulletin 58: 2846-2854;

[b] Genes putatively involved in pathogen-host interactions (PHI).

Table S9. Functional annotation of protein-coding genes of *O. sinensis*.

	Number	% Percentage
Total	7,939	100.00
Swissprot	5,372	67.67
NR*	7,242	91.22
InterPro	5,886	74.14
KEGG**	2,961	37.30
GO**	3,864	48.67
Annotated	7,697	96.95
Unannotated	242	3.05

* Blast hits with e-value ≤ 1e-5 and coverage ≥ 80% were retained;

** were directly retrieved from InterPro entry.

Table S11. Validation of gene models of *O. sinensis* by using homologous proteins, RNA-Seq datasets and BUSCO method.

**Evidence**	**Resources**	**Number**	**% Percent**
Homologous protein supported*	*Cordyceps militaris*	5,351	67.40
	Metarhizium anisopliae	5,586	70.36
	Metarhizium acridum	5,493	69.19
	Beauveria bassiana	5,528	69.63
	Total	6,441	81.13
RNA-Seq supported**	This study	5,677	71.51
Homology protein or RNA-Seq supported		7,173	90.35
**BUSCO groups from Ascomycota lineage *****			
Total BUSCO groups		1,315	100
Complete single-copy BUSCOs		1,241	94.4
Complete duplicated BUSCOs		2	0.2
Fragmented BUSCOs		47	3.6
Missing BUSCOs		25	1.8

Note: Blast with e-value < $1e^{-5}$ was used for searching against database;

* Identity >= 30% and Coverage >= 80%;

** Identity >= 90% and Coverage >= 80%.

*** The 1,315 BUSCO conserved genes used were collected from Ascomycota lineage.

Table S12. Annotation of conserved non-coding RNA genes in the *O. sinensis* genome.

ncRNA Type	# Loci	Average length (bp)	Total length (bp)	% of genome
tRNA	146	83.9	12,252	0.01%
rRNA (8S)	27	115	3,104	0.00%
rRNA (18S)	4	1774.3	7,097	0.01%
rRNA (28S)	2	3744.5	7,489	0.01%
SnoRNA	70	101.4	7,098	0.01%
snRNA	15	155	2,325	0.00%

Table S13. Summary of repeat sequences detected in *O. sinensis*. Detailed comparisons of TE contents between *O. sinensis* and the other three closely related species including *M. anisopliae*, *T. inflatum* and *C. militaris* were given in **Table S14**.

	Number	Length (bp)	Percent (%)*
**DNA transposons**	**8,256**	**3,175,406**	**2.73**
MULE	4,217	1,869,419	1.61
TcMar	2,752	880,805	0.76
CMC-EnSpm	921	233,927	0.20
PIF-Harbinger	366	191,255	0.16
**Retrotransposons**	**50,846**	**69,579,416**	**59.76**
LINE	1432	1,613,118	1.39
SINE	3	173	0.00
LTR-*Gypsy*	25,664	38,291,534	32.89
LTR-*Copia*	23,745	29,674,451	25.49
LTR-other	2	140	0.00
**Other repeats**	**43272**	**14180947**	**12.18**
Helitron	6	558	0.00
Low complexity	755	39,098	0.03
rRNA	13	7,655	0.01
Simple repeat	9,901	505,631	0.43
Unknown	32,597	13,628,005	11.71
**Total**	**102,374**	**86,935,769**	**74.67**

* Genome size of 116,423,023 bp was used to calculate the percentage for each type of repeats.

Table S15. Occurrence of simple sequence repeats (SSRs) in *O. sinensis* genome. Detailed information of SSRs identified in *O. sinensis* genome were list in **Table SS16.**

**Repeat type**	**Number**	**Proportion (%)**	**Total length (Kb)**	**Average length (bp)**
Mononucleotide	2,777	31.14	32.11	12
Dinucleotide	2,590	29.04	34.25	13
Trinucleotide	3,157	35.4	56.72	18
Tetranucleotide	71	0.8	2.40	34
Pentanucleotide	136	1.53	3.42	25
Hexanucleotide	187	2.1	8.03	43
**Total**	8,918	100	136.94	15

Table S17. Selected fungal genomes used for comparative analyses in this study.

Species	Genome size (Mb)	Number of Genes (#)	References
**Insect-pathogenic fungi**			
Ophiocordyceps sinensis	116.4	7,939	This study
Tolypocladium inflatum	30.3	9,998	Bushley et al. *PLoS Genetics*, 2014
Metarhizium anisopliae	39.0	10,582	Gao et al. *PLoS Genetics*, 2011
Metarhizium acridum	38.0	9,849	Gao et al. *PLoS Genetics*, 2011
Cordyceps militaris	32.2	9,684	Zheng et al. *Genome Biology*, 2011
Beauveria bassiana	33.7	10,366	Xiao et al. *Scientific Reports*, 2012
**Plant-pathogenic fungi**			
Fusarium graminearum	36.1	13,321	Cuomo et al. *Science*, 2007
Verticillium alfalfa	30.3	10,221	Klosterman et al. *PLoS Pathogens*, 2011
Magnaporthe grisea	37.9	11,054	Dean et al. *Nature*, 2005
Grosmannia clavigera	29.8	8,314	DiGuistini et al. *PNAS*, 2011
Sclerotinia sclerotiorum	38.3	14,503	Amselem et al. *PLoS Genetics*, 2011
Botrytis cinerea	42.3	16,448	Amselem et al. *PLoS Genetics*, 2011
**Outgroup**			
Saccharomyces cerevisiae	12.0	5,381	Goffeau et al. *Science*, 1997

Table S19. Gene number and repeat content in the 308 collinear blocks detected between *O. sinensis* and *C. militaris* genomes. More details about the 308 collinear blocks were shown in **Table S18**.

Level	Features	*C. militaris*	*O. sinensis*
**SEQ**	Total length (bp)	23,443,662	43,457,217
	Percentage [a]	72.7	37.3
**GENE**	**With collinearity**		
	Number (#)	3,992	3,992
	Percentage [a]	41.4	50.3
	Total length (bp)	7,790,657	7,468,635
	Ratio (%) [b]	33.2	17.2
	**Without collinearity**		
	Number (#)	3,259	2,127
	Percentage	33.8	26.8
	Total length (bp)	4,855,593	3,018,601
	Ratio (%) [b]	20.7	6.9
**REPEAT**	Total length (bp)	404,860	23,794,044
	Ratio (%) [c]	1.7	54.8
	LTR/*Gypsy* (%) [c]	4.8	40.4
	LTR/*Copia* (%) [c]	1.8	28.9
	LTR unclassified (%) [c]	0.1	11.5
	DNA (%) [c]	0.2	2.6

[a] represents percentage of genome;

[b] indicates ratio of gene length against total length of collinear blocks;

[c] refers to proportion of repeat length against total length of collinear blocks.

Table S20. Functional annotation of the 2,468 none-collinear genes lost in *O. sinensis* genome in comparison with *C. militaris*. The top 20 involved KEGG pathways were shown.

KEGG pathway	Description	Number
ko01230	Biosynthesis of amino acids	23
ko00330	Arginine and proline metabolism	15
ko01200	Carbon metabolism	14
ko00350	Tyrosine metabolism	13
ko04142	Lysosome	13
ko00360	Phenylalanine metabolism	11
ko04113	Meiosis – yeast	10
ko00380	Tryptophan metabolism	10
ko04111	Cell cycle – yeast	10
ko00230	Purine metabolism	9
ko00564	Glycerophospholipid metabolism	9
ko00520	Amino sugar and nucleotide sugar metabolism	8
ko05166	HTLV-I infection	8
ko00010	Glycolysis / Gluconeogenesis	8
ko00627	Aminobenzoate degradation	8
ko03010	Ribosome	8
ko01210	2-Oxocarboxylic acid metabolism	8
ko00643	Styrene degradation	8
ko00260	Glycine, serine and threonine metabolism	7
ko04114	Oocyte meiosis	7

Table S22. Overview of the gene families (clusters) identified among *O. sinensis* and other 12 fungal species. Species name were abbreviated as below: OSI, *O. sinensis*; MAN, *Metarhizium anisopliae*; MAC, *M. acridum*; CMI, *Cordyceps militaris*; BBA, *Beauveria bassiana*; FGR, *Fusarium graminearum*; MGR, *Magnaporthe grisea*; GCL, *Grosmannia clavigera*; SSC, *Sclerotinia sclerotiorum*; BCI, *Botrytis cinerea*; SCE, *Saccharomyces cerevisiae*.

Species	Gene number	Genes in families	Family number	Un-clustered	Average genes per family
OSI	7,939	7,198	6,754	741	1.10
TIN	9,881	8,206	7,531	1,675	1.20
MAC	9,780	9,103	8,434	677	1.07
MAN	10,385	9,634	8,755	751	1.08
BBA	10,224	9,236	8,357	988	1.11
CMI	9,601	8,513	7,911	1,088	1.13
FGR	13,260	10,144	8,877	3,116	1.31
GCL	8,267	7,023	6,612	1,244	1.18
MGR	10,983	8,619	7,748	2,364	1.27
BCI	16,244	9,709	8,888	6,535	1.67
SSC	13,983	9,272	8,669	4,711	1.51
VAL	10,179	8,119	7,462	2,060	1.25
SCE	6,476	4,151	3,287	2,325	1.56

Table S23. PFAM functional enrichment of the gene families that expanded in *O. sinensis*. PFAM domains for each gene were directly retrieved from InterPro entries. The statistical significances (*P*-values) were calculated using a Benjamini-corrected modified Fisher's exact test.

Pfam ID	Function description	#Gene	*P*-value
PF01328	Peroxidase, family 2	5	2.10E-07
PF03959	Serine hydrolase (FSH1)	4	6.00E-06
PF02102	Deuterolysin metalloprotease (M35) family	3	6.50E-05
PF00686	Starch binding domain	3	6.50E-05
PF00732	Glucose-Methanol-Choline (GMC) oxidoreductase	4	7.75E-05
PF05199	GMC oxidoreductase	4	7.75E-05
PF00067	Cytochrome P450	8	1.14E-04
PF05057	Putative serine esterase (DUF676)	3	1.59E-04
PF13520	Amino acid permease	4	3.41E-04
PF00135	Carboxylesterase family	3	5.37E-04
PF08787	Alginate lyase	2	6.54E-04
PF01743	Poly A polymerase head domain	2	6.54E-04
PF08618	Transcription factor Opi1	2	6.54E-04
PF03707	Bacterial signalling protein N terminal repeat	2	6.54E-04
PF01483	Proprotein convertase P-domain	2	6.54E-04
PF01370	NAD dependent epimerase/dehydratase family	3	8.43E-04
PF01485	IBR domain	3	8.43E-04
PF06772	Low temperature requirement A protein (LtrA)	2	1.93E-03
PF11954	Domain of unknown function (DUF3471)	2	1.93E-03
PF13402	Peptidase M60-like family	2	1.93E-03
PF05729	NACHT domain	3	2.35E-03
PF10282	Lactonase, 7-bladed beta-propeller	2	3.79E-03
PF11815	Domain of unknown function (DUF3336)	2	3.79E-03
PF11885	Protein of unknown function (DUF3405)	2	6.21E-03
PF12296	Hydrophobic surface binding protein A	2	6.21E-03
PF02133	Permease for cytosine/purines, uracil, thiamine	2	6.21E-03

PF01793	Glycolipid 2-alpha-mannosyltransferase	2	9.16E-03
PF01063	Aminotransferase class IV	2	9.16E-03
PF03198	Glucanosyltransferase	2	9.16E-03
PF00743	Flavin-binding monooxygenase-like	2	9.16E-03
PF01734	Patatin-like phospholipase	2	9.16E-03
PF13738	Pyridine nucleotide-disulphide oxidoreductase	2	1.26E-02
PF03151	Triose-phosphate Transporter family	2	1.26E-02
PF08031	Berberine and berberine like	2	1.26E-02
PF00188	Cysteine-rich secretory protein family	2	1.26E-02
PF04828	Glutathione-dependent formaldehyde-activating enzyme	2	1.26E-02
PF04488	Glycosyltransferase sugar-binding region containing DXD motif	2	1.26E-02
PF02668	Taurine catabolism dioxygenase TauD, TfdA family	2	1.65E-02
PF01048	Phosphorylase superfamily	2	1.65E-02
PF01753	MYND finger	2	1.65E-02
PF00144	Beta-lactamase	2	1.65E-02
PF01636	Phosphotransferase enzyme family	4	2.02E-02
PF08022	FAD-binding domain	2	2.09E-02
PF04389	Peptidase family M28	2	2.09E-02
PF08030	Ferric reductase NAD binding domain	2	2.09E-02
PF01794	Ferric reductase like transmembrane component	2	2.09E-02
PF06687	SUR7/PalI family	2	2.09E-02
PF01490	Transmembrane amino acid transporter protein	2	2.09E-02
PF00450	Serine carboxypeptidase	2	2.09E-02
PF00069	Protein kinase domain	7	4.26E-02
PF11807	Domain of unknown function (DUF3328)	2	4.86E-02
PF13489	Methyltransferase domain	2	4.86E-02
PF00651	BTB/POZ domain	2	4.86E-02
PF00082	Subtilase family	2	5.52E-02

Table S24. PFAM functional enrichment of gene families that contracted in *O. sinensis*. PFAM domains for each gene were directly retrieved from InterPro entries. The statistical significances (*P*-values) were calculated using a Benjamini-corrected modified Fisher's exact test.

Pfam ID	Function description	# Gene	*P*-value
PF00005	ATP-binding domain of ABC transporters	16	0.00E+00
PF00664	ABC transporter transmembrane region	12	0.00E+00
PF06422	CDR ABC transporter	4	1.77E-10
PF01061	ABC-2 type transporter	4	8.84E-10
PF14510	ABC-transporter extracellular N-terminal	3	5.21E-08
PF00324	Amino acid permease	3	1.45E-05
PF00306	ATP synthase alpha/beta chain, C terminal domain	2	1.55E-02

Table S25. Gene Ontology (GO) functional enrichment of *O. sinensis*-specific gene families. GO terms for each gene were directly retrieved from InterPro entries. The statistical significances (*P*-values) were calculated using a Benjamini-corrected modified Fisher's exact test.

GO term	Description	#Gene	*P*-value	FDR
GO:0005576	Extracellular region	5	2.36E-04	9.01E-03
GO:2001070	Starch binding	3	4.31E-04	9.01E-03
GO:0016491	Oxidoreductase activity	25	4.74E-04	9.01E-03
GO:0009405	Pathogenesis	4	1.34E-03	1.91E-02
GO:0016998	Cell wall macromolecule catabolic process	4	1.94E-03	2.21E-02
GO:0004222	Metalloendopeptidase activity	4	6.11E-03	3.86E-02
GO:0015297	Antiporter activity	2	6.77E-03	3.86E-02
GO:0015238	Drug transmembrane transporter activity	2	6.77E-03	3.86E-02
GO:0005615	Extracellular space	2	6.77E-03	3.86E-02
GO:0006855	Drug transmembrane transport	2	6.77E-03	3.86E-02

Table S26. **Number of peroxidase genes identified in seven fungal genomes.** Species were abbreviated as below: OSI, *O. sinensis*; MAN, *Metarhizium anisopliae*; MAC, *M. acridum*; CMI, *Cordyceps militaris*; BBA, *Beauveria bassiana*; SCE, *Saccharomyces cerevisiae*.

Species	Total peroxidase gene	Total protein-coding gene	Ratio (%)
OSI	42	7,939	0.53
TIN	36	9,998	0.36
CMI	28	9,684	0.29
BBA	36	10,366	0.35
MAN	42	10,582	0.40
MAC	41	9,849	0.42
SCE	21	5,381	0.39

Table S29. The total number of genes encoding protein kinases detected among *O. sinensis* and other nine fungal genomes. Species names were abbreviated as below: OSI, *O. sinensis*; MAN, *Metarhizium anisopliae*; MAC, *M. acridum*; CMI, *Cordyceps militaris*; BBA, *Beauveria bassiana*; Plant pathogens (shadowed): FGR, *Fusarium graminearum*; MGR, *Magnaporthe grisea*; GCL, *Grosmannia clavigera*; SSC, *Sclerotinia sclerotiorum*; BCI, *Botrytis cinerea*.

**Kinase group**	**OSI**	**MAN**	**MAC**	**CMI**	**BBA**	**FGR**	**MGR**	**GCL**	**BCI**	**SSC**
AGC (protein kinase A, G and C group)	8	13	15	14	15	12	13	12	13	10
Atypical kinases (Hisk, BRD, PDHK)	21	20	20	22	22	24	23	19	23	22
CMGC (CDK, MAPK, GSK3 and CLK kinases)	27	33	34	39	32	21	22	19	17	20
CAMK(Calcium/Calmodulin regulated kinases)	17	13	13	24	16	14	14	14	12	14
CK1 (Cell Kinase 1 group)	3	3	3	3	5	2	2	3	1	2
PKL (Protein Kinase-Like)	14	15	19	19	16	9	4	5	5	10
STE (MAP kinase cascade kinases)	11	15	14	19	16	11	13	13	9	12
TK(Tyrosine kinase)	0	1	2	2	0	1	2	0	0	0
TKL (Tyrosine kinase-like group)	1	0	1	0	1	3	0	1	0	0
Other	31	31	31	30	30	31	29	24	30	35
**Total**	**133**	**144**	**152**	**172**	**153**	**128**	**122**	**110**	**110**	**125**

Table S30. Distribution of Carbohydrate-degrading enzymes among *O. sinensis* and other nine fungal genomes arranged by Glycoside Hydrolases (GH) gene family. Species names were abbreviated as below: OSI, *O. sinensis*; MAN, *Metarhizium anisopliae*; MAC, *M. acridum*; CMI, *Cordyceps militaris*; BBA, *Beauveria bassiana*; Plant pathogens (shadowed): FGR, *Fusarium graminearum*; MGR, *Magnaporthe grisea*; GCL, *Grosmannia clavigera*; SSC, *Sclerotinia sclerotiorum*; BCI, *Botrytis cinerea*.

GH family	OSI	MAN	MAC	CMI	BBA	FGR	MGR	GCL	BCI	SSC
GH1	1	3	3	1	1	3	2	1	4	3
GH2	3	6	6	6	7	10	8	3	2	2
GH3	6	9	7	9	11	22	18	15	16	13
GH5	3	8	8	7	7	14	13	8	16	14
GH6	1	0	0	0	0	1	3	0	1	2
GH7	0	0	0	0	0	2	7	4	3	3
GH10	0	0	0	0	1	5	6	0	2	2
GH11	0	0	0	0	0	2	5	1	2	3
GH12	1	1	1	1	1	4	3	5	3	4
GH13	3	5	4	4	4	7	10	4	8	9
GH15	1	2	2	2	2	3	2	2	3	4
GH16	13	19	18	20	24	23	18	12	21	19
GH17	5	5	4	4	4	4	7	3	5	6
GH18	15	27	18	22	21	18	15	11	10	14
GH20	2	2	2	3	5	3	3	2	1	1
GH23	0	0	0	1	0	0	0	0	0	1
GH24	0	2	1	0	0	0	1	0	0	0
GH25	0	2	2	1	1	0	0	0	1	1
GH26	0	0	0	0	0	0	0	0	2	1

GH27	1	2	2	0	2	2	3	0	4	3
GH28	1	1	1	1	1	6	3	6	21	17
GH29	0	1	2	2	2	1	4	1	0	0
GH30	0	0	0	0	0	0	1	1	0	0
GH31	2	6	6	4	6	8	6	1	5	6
GH32	1	2	2	1	1	6	4	0	3	1
GH33	0	1	1	1	0	1	1	0	0	0
GH35	1	3	3	3	3	3	0	2	4	4
GH36	1	1	1	1	1	2	0	0	0	0
GH37	1	2	2	2	2	3	2	2	1	1
GH38	1	1	1	1	1	1	2	1	1	1
GH39	0	0	0	0	0	0	1	0	0	0
GH43	1	1	3	2	3	18	20	8	6	5
GH45	0	0	0	0	1	1	1	1	2	2
GH47	7	8	9	7	7	10	9	6	10	8
GH51	0	0	0	0	0	2	3	2	2	2
GH53	0	0	0	0	0	1	1	1	2	2
GH54	0	1	0	1	1	1	1	0	1	1
GH55	2	5	4	8	8	3	4	3	4	4
GH62	0	0	0	0	0	1	4	0	1	0
GH63	1	1	1	2	2	1	1	2	1	1
GH64	3	1	1	2	1	2	2	2	2	2
GH65	0	1	1	1	0	0	0	0	2	1
GH67	0	0	0	0	0	1	1	0	0	0
GH71	0	1	1	0	0	0	1	0	8	8
GH72	6	5	5	5	6	3	5	5	6	6
GH74	1	2	1	0	1	4	6	2	1	2
GH75	1	3	3	2	2	1	1	0	0	0
GH76	8	14	13	10	10	8	9	9	10	12

GH78	0	0	0	0	0	7	3	5	7	4
GH79	0	1	1	1	2	1	2	1	2	2
GH81	1	1	2	1	1	1	2	1	1	1
GH84	0	1	1	1	1	0	0	0	0	0
GH88	0	1	0	1	1	1	1	0	1	0
GH89	0	2	1	2	2	0	0	0	2	1
GH92	3	5	4	4	4	0	6	3	5	5
GH93	0	0	0	0	0	2	1	0	1	1
GH94	0	0	0	0	0	0	1	0	0	0
GH95	0	0	0	0	1	3	2	0	3	2
GH99	0	1	0	0	0	0	0	0	0	0
GH105	1	3	1	0	0	3	3	1	2	1
GH106	0	0	0	0	0	1	1	1	1	1
GH109	3	5	7	6	9	15	7	5	5	5
GH114	0	1	1	2	1	2	1	0	1	1
GH115	0	1	1	0	0	3	4	0	2	1
GH117	0	1	0	0	0	0	0	0	0	0
GH121	0	0	0	1	1	1	0	0	0	0
GH125	2	3	3	2	3	3	4	3	4	3
GH127	1	1	1	1	1	2	2	2	1	0
GH128	2	4	3	2	3	4	4	3	4	3
GH131	0	0	0	0	0	1	8	1	2	2
GH132	2	2	2	2	2	2	2	2	2	0
**Total**	**108**	**187**	**167**	**163**	**182**	**263**	**271**	**154**	**243**	**224**

Table S31. Distribution of Carbohydrate-degrading enzymes among *O. sinensis* and other nine fungal genomes arranged by Carbohydrate-Binding Modules (CBM) gene family. Species names were abbreviated as below: OSI, *O. sinensis*; MAN, *Metarhizium anisopliae*; MAC, *M. acridum*; CMI, *Cordyceps militaris*; BBA, *Beauveria bassiana*; Plant pathogens (shadowed): FGR, *Fusarium graminearum*; MGR, *Magnaporthe grisea*; GCL, *Grosmannia clavigera*; SSC, *Sclerotinia sclerotiorum*; BCI, *Botrytis cinerea*.

CBM family	OSI	MAN	MAC	CMI	BBA	FGR	MGR	GCL	BCI	SSC
CBM1	0	4	2	1	3	11	21	2	15	18
CBM6	0	0	0	0	0	1	2	0	0	0
CBM9	0	0	1	0	0	0	0	0	0	0
CBM13	1	1	2	4	6	2	2	1	2	3
CBM16	0	0	0	0	0	4	0	0	0	0
CBM18	4	7	2	9	8	17	50	4	10	18
CBM19	0	0	0	0	1	0	0	0	0	0
CBM20	4	1	1	1	1	2	3	2	3	4
CBM21	1	2	2	1	2	2	1	1	1	1
CBM22	0	0	0	0	0	3	0	0	0	0
CBM24	1	2	1	0	0	2	0	0	11	9
CBM32	0	1	1	1	2	2	1	1	2	0
CBM35	0	0	0	0	0	2	2	0	2	1
CBM40	0	0	0	0	0	0	1	0	0	0
CBM42	0	1	0	1	1	1	2	0	1	1
CBM43	2	2	2	2	3	1	2	1	1	1
CBM46	0	0	0	0	0	0	0	0	1	1
CBM48	1	1	1	1	1	1	1	1	0	1
CBM50	11	12	1	10	10	7	7	8	1	0
CBM52	0	0	0	0	1	0	1	1	0	0
CBM61	0	0	0	0	0	1	0	0	0	2
CBM63	0	0	0	0	0	3	1	1	0	1
CBM66	0	1	2	4	5	0	1	0	1	0
CBM67	0	0	0	0	0	4	1	1	3	1
**Total**	**25**	**35**	**18**	**35**	**44**	**66**	**99**	**24**	**54**	**62**

Table S32. Distribution of Carbohydrate-degrading enzymes among *O. sinensis* and other nine fungal genomes arranged by Carbohydrate Esterases (CE) gene family. Species names were abbreviated as below: OSI, *O. sinensis*; MAN, *Metarhizium anisopliae*; MAC, *M. acridum*; CMI, *Cordyceps militaris*; BBA, *Beauveria bassiana*; Plant pathogens (shadowed): FGR, *Fusarium graminearum*; MGR, *Magnaporthe grisea*; GCL, *Grosmannia clavigera*; SSC, *Sclerotinia sclerotiorum*; BCI, *Botrytis cinerea*.

CE family	OSI	MAN	MAC	CMI	BBA	FGR	MGR	GCL	BCI	SSC
CE1	11	19	12	17	16	20	28	15	14	13
CE2	0	0	0	0	0	1	2	0	2	1
CE3	3	2	2	2	2	7	7	2	2	3
CE4	6	4	3	4	3	9	12	6	6	5
CE5	2	2	2	4	5	12	18	1	11	8
CE7	0	1	0	0	0	0	0	1	0	1
CE8	0	0	0	0	0	7	1	1	5	5
CE9	1	1	1	1	2	1	1	2	2	2
CE10	25	38	33	33	39	62	47	32	54	42
CE12	0	0	2	2	1	5	3	1	4	5
CE14	1	2	2	1	1	0	1	1	1	1
CE15	0	0	0	0	1	0	1	0	0	1
CE16	1	2	1	1	1	5	2	0	6	6
**Total**	**50**	**71**	**58**	**65**	**71**	**129**	**123**	**62**	**107**	**93**

Table S33. Functional enrichment analysis of the 411 differentially expressed genes among the three developmental stages of *O. sinensis*.

PFAM	Description	#Gene	*P*-value	FDR
PF00722	Glycosyl hydrolases family 16	6	6.59E-06	3.30E-04
PF00702	Haloacid dehalogenase-like hydrolase	6	7.26E-05	1.81E-03
PF00122	E1-E2 ATPase	6	2.02E-04	3.08E-03
PF01370	NAD dependent epimerase / dehydratase family	4	2.66E-04	3.08E-03
PF00067	Cytochrome P450	10	3.08E-04	3.08E-03
PF08740	BCS1 N terminal	3	3.73E-04	3.11E-03
PF00690	Cation transporter/ATPase	4	1.12E-03	7.01E-03
PF00860	Permease family	2	6.14E-03	2.79E-02
PF00246	Zinc carboxypeptidase	2	6.14E-03	2.79E-02
PF12224	Putative amidoligase enzyme	2	6.14E-03	2.79E-02
PF07690	Major Facilitator Superfamily	10	1.15E-02	4.26E-02
PF00806	Pumilio-family RNA binding repeat	2	1.19E-02	4.26E-02
PF11700	Vacuole effluxer Atg22 like	2	1.19E-02	4.26E-02

Table S36. **Validation of the occurrences of two types of mating-type genes (*MAT1-1* and *MAT1-2*) in 31 populations of *O. sinensis* using reads mapping method.** Sequence coverage is shown for each mating-type gene. Of the 31 examined individuals, we found that 27 could be well confirmed to harbor those two types of mating-type genes. The absence of *MAT1-1* and/or *MAT1-2* idiomorph for QH02, QH05, GS03 and SC08 (filled in gray) may be caused by the heterogeneity and/or insufficient sequencing in those regions.

**Populations**	***MAT1-1-1***	***MAT1-1-2***	***MAT1-1-3***	***MAT1-2-1***
GS01	99.92	100.00	100.00	90.08
GS02	99.92	100.00	100.00	10.74
GS03	99.92	100.00	100.00	0.00
GS04	99.92	100.00	100.00	97.20
GS05	25.08	18.72	47.51	100.00
QH01	99.92	100.00	100.00	100.00
QH02	0.00	0.00	0.00	100.00
QH03	99.92	100.00	100.00	100.00
QH04	98.03	97.93	98.29	100.00
QH05	0.00	0.00	0.00	100.00
QH06	99.92	100.00	100.00	100.00
QH07	99.92	100.00	100.00	100.00
QH08	0.00	2.27	10.67	100.00
SC01	98.85	99.80	100.00	98.72
SC02	99.92	100.00	100.00	98.13
SC03	99.92	100.00	100.00	84.60
SC04	100.00	100.00	100.00	100.00
SC06	99.92	100.00	100.00	99.07
SC07	99.92	100.00	100.00	47.96
SC08	88.03	97.73	100.00	0.00

TB01	91.72	79.15	100.00	100.00
TB02	96.72	99.00	100.00	28.35
TB03	65.74	45.44	80.51	100.00
TB04	90.98	97.40	54.05	100.00
TB05	84.26	89.74	96.59	100.00
TB06	99.92	100.00	100.00	100.00
TB07	95.82	99.07	100.00	76.20
TB08	99.92	100.00	100.00	100.00
YN01	99.92	100.00	100.00	74.80
YN02	99.92	99.40	100.00	72.00
YN03	99.84	100.00	100.00	94.75

Table S37. **Geographical information of the 31 populations of *O. sinensis* for genome re-sequencing collected from nearly all its known distribution range, including Qinghai (QH), Sichuan (SC), Yunnan (YN) and Gansu (GS) provinces and Tibet Autonomous Region (TB).**

Populations	Locality	Date	Longitude	Latitude	Altitude (m)
SC01	SC-SD	2007.6.16	100°15.823′	32°14.521′	N/A
SC02	SC-GZ	2007.6.17	100°16.069′	31°49.890′	N/A
SC03	SC-RT	2007.6.15	100°48.464′	32°11.488′	4,407
SC04	SC-HY	2007.6.13	102°29.852′	32°46.837′	N/A
SC06	SC-SF	2007.4.28	103°48.143′	32°40.588′	4,041
SC07	SC-KD	2007.6.11	101°48.335′	30°06.555′	4,474
SC08	SC-KD	2007.6.11	101°56.256′	29°33.556′	4,416
TB01	TB-NQ	2007.6.22	92°40.254′	31°15.581′	4,897
TB02	TB-LZ	N/A	94°22.205'	29°40.811'	N/A
TB03	TB-LZ	2007.6.26	94°46.088′	29°28.035′	4,260
TB04	TB-LZ	2009.6.18	94°38.557′	29°37.034′	N/A
TB05	TB-RW	2009.6.13	96°45.310′	29°30.045′	N/A
TB06	TB-DDL	2009.6.12	98°00.114′	29°42.623′	N/A
TB07	TB-JD	2007.6.17	98°04.384′	31°39.273′	4,336
TB08	TB-BM	2009.6.17	97°17.617′	30°09.008′	N/A
QH01	QH-JZ	2007.6.15	101°11.364′	33°19.344′	4,170
QH02	QH-MY	2008.6.24	101°17.788′	37°42.239′	3,457
QH03	QH-YS	2008.6.29	96°39.881′	33°04.412′	4,417
QH04	QH-CD	2008.6.29	97°47.170′	33°33.369′	4,400
QH05	QH-HM	2008.6.26	99°44.347′	36°39.469′	3,955
QH06	QH-QLS	2008.6.23	98°59.590′	38°04.007′	4,357
QH07	QH-THE	2008.6.28	99°50.590′	37°32.059′	3,848
QH08	QH-MYG	2008.6.24	101°26.097′	37°43.219′	3,822

GS01	GS-MQ	2007.6.13	101°58.572′	34°04.381′	4,121
GS02	GS-TZ	2007.7	102°38.431′	37°05.449′	3,809
GS03	GS-TZ	2008.6.20	102°38.431′	37°04.381′	4,121
GS04	GS-GL	2008.6.21	102°44.047′	37°27.324′	2,892
GS05	GS-GL	2008.6.19	102°44.047′	37°27.324′	2,892
YN01	YN-ZD	2009.5	98°59.993′	28°29.492′	N/A
YN02	YN-DQ	2010.5.22	99°46.248′	28°35.960′	4,535
YN03	YN-XZD	2008.5.27	99°40.307′	27°35.632′	3,860

Table S38. **Statistics of genome re-sequencing data for 31 populations of *O. sinensis*.** DNA libraries for each sample were constructed and sequenced using Illumina HiSeq 2000 platform. Approximate 10-fold coverage for each individual was generated.

**Sample**	**Read counts**	**Read bases (bp)**	**Read base (Gb)**	**Coverage (×)***
SC01	8,982,901	1,796,580,200	1.80	14.98
SC02	5,619,567	1,123,913,400	1.12	9.37
SC03	6,160,582	1,232,116,400	1.23	10.27
SC04	6,373,435	1,274,687,000	1.28	10.63
SC06	6,400,919	1,280,183,800	1.28	10.67
SC07	5,936,436	1,187,287,200	1.19	9.89
SC08	5,983,056	1,196,611,200	1.20	9.98
TB01	4,942,342	988,468,400	0.99	8.23
TB02	2,898,863	579,772,600	0.58	4.83
TB03	5,416,428	1,083,285,600	1.08	9.03
TB04	5,965,370	1,193,074,000	1.19	9.94
TB05	5,299,402	1,059,880,400	1.06	8.83
TB06	5,655,134	1,131,026,800	1.13	9.43
TB07	5,083,908	1,016,781,600	1.02	8.48
TB08	8,054,627	1,610,925,400	1.61	13.43
QH01	5,300,440	1,060,088,000	1.06	8.83
QH02	5,715,330	1,143,066,000	1.14	9.53
QH03	5,951,975	1,190,395,000	1.19	9.92
QH04	7,390,304	1,478,060,800	1.48	12.32
QH05	6,630,037	1,326,007,400	1.33	11.05
QH06	6,743,654	1,348,730,800	1.35	11.24
QH07	5,626,471	1,125,294,200	1.13	9.38

QH08	5,302,718	1,060,543,600	1.06	8.84
GS01	5,476,224	1,095,244,800	1.10	9.13
GS02	5,939,892	1,187,978,400	1.19	9.90
GS03	5,455,141	1,091,028,200	1.09	9.09
GS04	6,307,752	1,261,550,400	1.26	10.52
GS05	8,863,865	1,772,773,000	1.77	14.78
YN01	6,221,786	1,244,357,200	1.24	10.37
YN02	3,099,847	619,969,400	0.62	5.17
YN03	4,586,553	917,310,600	0.92	7.64
**Total**	183,384,959	36,676,991,800	36.68	305.64

* The estimated genome size of 120 Mb was used.

Table S39. **Summary of variations (SNP and Indel) calling.** The SNPs and Indels were called using a combination of samtools (version 0.1.19) and GATK pipeline (https://www.broadinstitute.org/gatk/). The synonymous and non-synonymous SNPs were identified using snpEff (version 4.1f).

**Basic information**	
Total number of SNPs	816,960
Total number of insertions	23,079
Total number of deletions	25,013
**Genomic distribution**	
Intergenic	580,685
Intronic	46,054
Coding	190,221
**Effects for amino acids**	
non-synonymous	88,224
synonymous	101,997
non-synonymous / synonymous	0.86

Table S43. Statistic of genome assembly of the GS05 strain of *O. sinensis*. The GS05 strain that is one of our re-sequenced samples and resides at the lowest altitude (2,892 m above sea level) was assembled using SOAPdenovo (version 2.04).

Sequence type	GS05 strain
**Contig**	
Number (#)	137,622
Total length (bp)	64,328,842
N50 (bp)	1,348
Largest (bp)	37,515
**Scaffold**	
Number (#)	101,641
Number (> 2Kb)	7,991
Total length (bp)	65,986,092
N50 (bp) [a]	4,292
N90 (bp) [a]	1,461
Largest (bp)	40,777
Gap length (bp)	1,657,250
GC content (%)	43.14
Completeness (%) [b]	54.99

[a] The N50 and N90 values of the genome assembly were calculated using the fragment with its length ≥ 1 kb;

[b] The estimated genome size of 120 Mb was used.

Table S44. Summary of repeat sequences detected in GS05 strain of *O. sinensis*. The repeat sequences of GS05 genome were annotated using the same method as described in **Supplementary section 2.2**.

	Number	Length (bp)	Percent (%)*
**DNA transposons**	**5,675**	**1,850,492**	**2.80**
MULE	3,466	1,114,753	1.69
TcMar	1,457	506,478	0.77
CMC-EnSpm	416	86,414	0.13
PIF-Harbinger	336	142,847	0.22
**Retrotransposons**	**105,570**	**42,099,819**	**63.80**
LINE	1,599	537,640	0.81
SINE	4	315	0.00
LTR-*Gypsy*	57,814	21,997,393	33.34
LTR-*Copia*	46,151	19,564,331	29.65
LTR-other	2	140	0.00
**Other repeats**	**28,074**	**7,808,221**	**11.83**
Helitron	5	597	0.00
Low complexity	315	15,944	0.02
rRNA	21	5,381	0.01
Simple repeat	4,154	226,845	0.34
Unknown	23,579	7,559,454	11.46
**Total**	**139,319**	**51,758,532**	**78.44**

* Genome size of 65,986,092 bp was used to calculate the percentage for each type of repeats.
